# RET signalling provides tumorigenic mechanism and tissue specificity for AIP-related somatotrophinomas

**DOI:** 10.1038/s41388-021-02009-8

**Published:** 2021-09-29

**Authors:** Angela R. Garcia-Rendueles, Miguel Chenlo, Fernando Oroz-Gonjar, Antonia Solomou, Anisha Mistry, Sayka Barry, Carles Gaston-Massuet, Montserrat Garcia-Lavandeira, Sihara Perez-Romero, Maria Suarez-Fariña, Alberto Pradilla-Dieste, Carlos Dieguez, Patrick Mehlen, Márta Korbonits, Clara V. Alvarez

**Affiliations:** 1 Neoplasia & Endocrine Differentiation P0L5, Centro de Investigación en Medicina Molecular y Enfermedades Crónicas (CIMUS), University of Santiago de Compostela (USC), Santiago de Compostela, Spain; 2 Department of Endocrinology, William Harvey Research Institute, Barts and London School of Medicine and Dentistry, Queen Mary University of London, London, UK; 3 Patrick Mehlen, Apoptosis, Cancer and Development Laboratory- Equipe labellisée ‘La Ligue’, LabEx DEVweCAN, Institut PLAsCAN, Centre de Recherche en Cancérologie de Lyon, INSERM U1052-CNRS UMR5286, Université de Lyon, Centre Léon Bérard, 69008 Lyon, France

**Keywords:** Pituitary tumours, Apoptosis

## Abstract

It is unclear how loss-of-function germline mutations in the widely-expressed co-chaperone *AIP*, result in young-onset growth hormone secreting pituitary tumours. The RET receptor, uniquely co-expressed in somatotrophs with PIT1, induces apoptosis when unliganded, while RET supports cell survival when it is bound to its ligand. We demonstrate that at the plasma membrane, AIP is required to form a complex with monomeric-intracellular-RET, caspase-3 and PKCδ resulting in PIT1/CDKN2A-ARF/p53-apoptosis pathway activation. AIP-deficiency blocks RET/caspase-3/PKCδ activation preventing PIT1 accumulation and apoptosis. The presence or lack of the inhibitory effect on RET-induced apoptosis separated pathogenic *AIP* variants from non-pathogenic ones. We used virogenomics in neonatal rats to demonstrate the effect of mutant AIP protein on the RET apoptotic pathway in vivo. In adult male rats altered AIP induces elevated IGF-1 and gigantism, with pituitary hyperplasia through blocking the RET-apoptotic pathway. In females, pituitary hyperplasia is induced but IGF-1 rise and gigantism are blunted by puberty. Somatotroph adenomas from pituitary-specific *Aip*-knockout mice overexpress the RET-ligand GDNF, therefore, upregulating the survival pathway. Somatotroph adenomas from patients with or without *AIP* mutation abundantly express GDNF, but *AIP*-mutated tissues have less *CDKN2A-ARF* expression. Our findings explain the tissue-specific mechanism of AIP-induced somatotrophinomas and provide a previously unknown tumorigenic mechanism, opening treatment avenues for *AIP*-related tumours.

## Introduction

The pituitary gland contains five different endocrine cell types with growth hormone (GH)-secreting cells, so-called somatotrophs, contributing to ~50% of the gland. The transcription factor PIT1 is required for the expression and secretion of GH. PIT1 is also essential for somatotroph differentiation during embryonic development and for proliferative and secretory response to the hypothalamic factor GH-releasing hormone (GHRH) [1]. However, when PIT1 is excessively accumulated in a somatotroph cell, apoptosis is initiated resulting in the elimination of the cell [2, 3].

Somatotroph tumours secrete excess GH leading to acromegaly or gigantism if started before epiphyseal closure and need to be treated with surgery and pharmacological treatment [4–6]. Excess GH and its target hormone IGF-1 causes enlarged organs, bone and joint deformation, hypertension, diabetes and significantly reduced life expectancy if left untreated [4, 5].

Germline mutations in the *AIP* gene is a known cause of familial isolated pituitary adenoma (FIPA), mostly somatotrophinomas [7–11]. Pituitary tumours associated with *AIP* mutations (*AIP*-FIPA) are often invasive macroadenomas presenting in the second decade. They are prone to pituitary apoplexy and respond poorly to first-generation somatostatin analogue therapy. Genetic screening and clinical follow-up of carriers lead to earlier disease detection and better outcomes [12].

AIP is a widely expressed co-chaperone and there is no clear explanation why it predisposes to specifically pituitary somatotroph or lactotroph tumorigenesis and no other tumours. It is quite clear that the cAMP pathway is key in somatotroph tumorigenesis and several lines of experimental results suggested upregulated cAMP pathway, reduced G_alphai-2_ expression and abnormal phosphodiesterase interactions, but none represent pituitary-specific mechanisms [13–16]. Being a chaperone, not surprisingly several novel interacting proteins have been described for AIP [17] and miR34 has also emerged in two independent studies as a related key microRNA [18, 19]. Knowledge derived from cell-based experiments considers *AIP* as a tumour suppressor in some cell types [7, 8, 15], but not all [20]. Global knockout in mice, *Drosophila* and *C. elegans* shows embryonic lethality [21–23]. The global *Aip* heterozygous knockout mouse or conditional knockout restricted to *Gh*- (somatotrophs) or *Hex1*-(pituitary) expressing cells present a phenotype with pituitary tumours [24–27].

The RET receptor is a tyrosine kinase with four ligands (GDNF, NRTN, ARTM, PSPN) and four respective co-receptors (GFRα1-4). RET is implicated in several diseases from neuroendocrine cancer, such as medullary thyroid carcinomas and pheochromocytomas, to developmental defects in parasympathetic neurons and the urinary tract. Normal and tumorous somatotroph cells express RET, GDNF and GFRα1 [28–30]. RET is active both in the absence and presence of its ligands, therefore giving rise to two different pathways. Both can be activated in somatotroph cells. In the absence of the ligand GDNF, RET is intracellularly processed by caspase-3 and induces the *PIT1* promoter with massive PIT1 accumulation, resulting in the upregulation of the RET/PIT1/ARF/p53-apoptotic pathway [2, 3, 30, 31]. At the *PIT1* promoter, activation is mediated by conserved CRE and c/EBP binding sites is mediated by conserved CRE and c/EBP binding sites, following activation of phospho-JNK [2]. Similarly, induction of the *CDKN2A-ARF* promoter is mediated by a conserved PIT1 binding site [3]. On the other hand, when RET is stimulated by its ligand GDNF, RET dimerises and upregulates the GDNF/RET/AKT survival pathway via its tyrosine-kinase activity, while maintaining PIT1 expression at basal levels [2, 30, 31]. The two pathways are independent, as kinase-dead RET point mutants in the presence of GDNF are unable to activate the AKT pathway, but keep stimulating the apoptotic pathway [2]. Thus, RET is a dependence receptor [32, 33] for somatotroph cells, as they need the constant presence of GDNF to survive. Both the RET-related survival (presence of RET ligand) and apoptosis (lack of RET ligand) pathways are conserved in sporadic somatotroph tumours [31] and recent results demonstrated that i) overexpression of GDNF is a hallmark of somatotroph tumours, and ii) lower level ARF expression is a prognostic marker for resistance to therapy [30].

Although it was shown that RET can bind AIP in rat whole pituitary extracts, molecular studies performed in HEK293 cells with RET or AIP were unable to demonstrate any functionality of this interaction, as it was not affected by any *RET* or *AIP* point mutations [34]. Previously we had demonstrated that proliferative responses to GHRH varied depending on the cell phenotype, and this was specifically caused by differences in PIT1 expression [1].

We hypothesise that AIP has a role in RET-related survival and apoptotic pathways specifically in somatotroph cells. In the current study, we have explored the action of wild-type human AIP (wtAIP), missense *AIP* variants identified in pituitary adenoma patients and AIP repression (siRNA or knockout) on the RET/PIT1/ARF/p53-apoptosis pathway, using well-characterized somatotroph cell lines, primary pituitary cultures, a new rat model of gigantism/acromegaly and a novel pituitary-specific *Aip* knockout mice.

## Results

### Functional classification of *AIP* mutations into two groups: protein present or absent

**Fig. 1:**
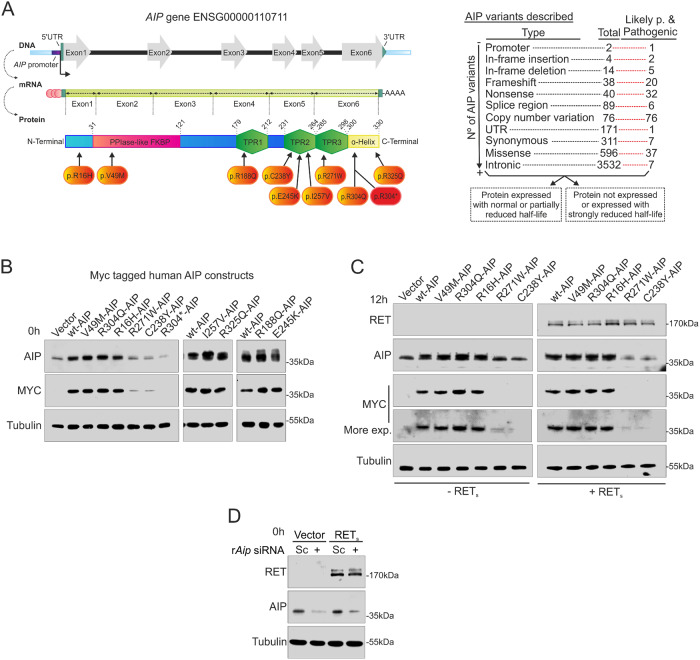
Strategy to study the two groups of AIP variants: shorter and normal half-life. **A** Left: Cartoon showing the human *AIP* gene, mRNA and protein. The studied variants are indicated. **Right:** *AIP* gene variants described in Ensembl (data retrieved at 06/20/2020), indicating how many of each type of variants is considered as pathogenic, likely pathogenic (Likely p.) as opposed to Variants of Uncertain Significance, likely non-benign or benign. Variants could be grouped based on their protein half-life. **B** Wild-type human AIP (wtAIP) or variant AIP were expressed in the pituitary somatotroph rat cell line GH4C1 with an N-terminal MYC-tag. Western blot against AIP detected endogenous rat AIP together with exogenous human AIP. MYC western blot exclusively detected transfected human AIP. R304* AIP is undetectable under our experimental conditions presenting a very-short half-life, R271W and C238Y present short half-life in comparison to wtAIP, while R16H, V49M, R188Q, E245K, I257V, R304Q and R325Q AIP variants present normal half-life compared to wtAIP. **C** Co-transfection of RET receptor does not alter the half-life distribution of wtAIP or AIP variants. **D** *Aip* siRNA is able to downregulate endogenous rat AIP protein expression as a model for AIP deficiency in GH4C1 cells.

AIP is coded by a gene on 11q13 spanning six exons (Fig. 1A). Various genetic alterations have been found in patients with pituitary adenomas, such as whole gene or exon deletions, promoter mutations, insertions/deletions, splicing mutations as well as nonsense and missense mutations. While loss-of-function truncating mutations are listed as pathogenic in databases, many missense variants are listed as Variants of Uncertain Significance due to lack of convincing clinical data and shortage of functional data (Fig. 1B). We have selected nine *AIP* variants identified in patients (Supplementary Table 1), some with previous attempts at functional characterization [8, 9, 14, 22, 35]. *In silico* analysis with 17 available algorithms showed lack of consensus regarding the pathogenicity of these variants (Supplementary Table 2).

The selected human *AIP* variants, as well as wtAIP, were cloned in a vector including an N-terminal MYC-tag. Variants were transfected into rat GH4C1 somatotroph cells, which endogenously express PIT1, GH and RET pathway members GDNF and GFRα1 at similar levels to normal rat pituitary tissue (Supplementary Figure 1B–D). However, GH4C1 cells poorly express RET receptor at both mRNA and protein levels (Supplementary Figure 1B, C). These data are in keeping with our previous studies using Northern blots, and our previous repeatedly failed attempts to stably express RET in GH4C1, leading to the discovery of the RET-apoptotic pathway in somatotroph cells [2]. When cells were deprived of serum (0.1% FBS), intracellular sorting of GDNF and GFRα1 proteins was arrested, and GH expression was downregulated (Supplementary Figure 1C, D).

Anti-AIP antibody detected both endogenous rat and exogenous human AIP protein, while transfected AIP protein could be detected through the MYC-tag antibody as well. Half-life of the nine selected *AIP* variants in pituitary cells did not change in presence of human RET (Fig. 1B, C), similarly to data previously studied in HEK293 cells for some of the variants [17, 36]. Rat *Aip* siRNA silencing was chosen as a strategy to explore the activity of those unstable AIP variants (Fig. 1D).

### Pathogenic *AIP* variants or *Aip* repression block the RET/PIT1/*CDKN2A*-ARF/p53 apoptotic pathway specifically in pituitary somatotrophs

Our previous results have characterized precisely that GH4C1 cells transfected with RET, either the short (RET_S_, 1072 amino acids) or the long (RET_L,_ 1116 amino acids) isoforms, start to die 48 h after deprivation of RET ligand GDNF, and this was prevented by addition of exogenous recombinant GDNF.

**Fig. 2:**
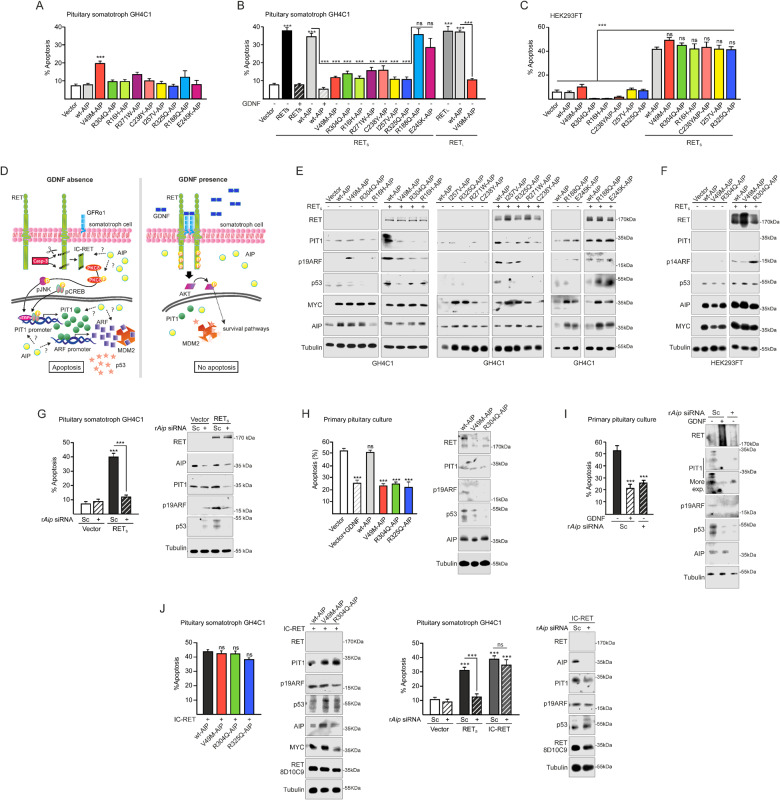
Variant *AIP* or *Aip* downregulation blocks the physiological RET/PIT1/*CDKN2A*-ARF/p53 apoptosis pathway. **A** Expression of wt or variant *AIP* does not affect basal rate of apoptosis when GH4C1 cells are deprived of GDNF (0.5% FBS). V49M is the only variant causing some apoptosis. **B** When RET_S_ is transfected in GH4C1, serum deprivation induces the RET-apoptotic pathway (black bar) that is blocked by the RET ligand GDNF (black hatched). Expression of human wtAIP together with RET maintain the apoptotic pathway which is also blocked by GDNF (grey colour, black hatched). Expression of variant AIP (R16H, V49M, R271W, C238Y, I257V, R325Q, coded coloured bars) blocks RET-induced apoptosis in the absence of GDNF. RET_L_ expression follows the same pattern with strong apoptosis in the presence of wtAIP (grey bar) blocked by variant AIP in the absence of GDNF. Expression of R188Q or E245K (coded coloured bars), does not block RET-induced apoptosis. **C** A similar experiment in the embryonic kidney cell line HEK293T cells have different results. RET induces apoptosis in the presence of wtAIP but also in the presence of any *AIP* variant. **D** Cartoon showing the known RET pathways in pituitary somatotroph cells: left, RET/PIT1/*CDKN2A*-ARF/p53 apoptosis pathway; right, RET/GDNF-AKT survival pathway. AIP alteration could affect any step in the apoptotic pathway or directly induce the survival pathway. **E** AIP variants that block apoptosis also prevent RET-induced *Pit1* overexpression, p19Arf increase and p53 accumulation. The two AIP variants that did not block apoptosis did not alter RET-induced *Pit1* overexpression, p19Arf increase and p53 accumulation. **F** HEK293T does not express PIT1 and express large T-antigen. However, RET expression induces ARF and p53 accumulation in the presence of WT or variant AIP. **G** In GH4C1 repression of endogenous *Aip* blocks RET-induced apoptosis in parallel to PIT1, ARF and p53 repression. **H**, **I** Similar results were obtained in rat male primary pituitary culture with 45%-50% somatotroph cells expressing endogenous rat RET receptor. **H** When cultures are serum-deprived (no GDNF) apoptosis is induced, and this is prevented by the addition of rGDNF. Transfection of wtAIP does not alter apoptosis, nor the apoptotic pathway through PIT1/ARF/p53 accumulation. Transfection of variant *AIP* blocks PIT1/ARF/P53 increase and apoptosis. **I** Repressing *Aip* with siRNA blocks PIT1/ARF/p53 increase and apoptosis as well as GDNF addition. **J** In GH4C1, direct transfection of the short caspase-processed intracellular RET fragment, IC-RET, induces the RET/PIT1/*CDKN2A*-ARF/p53 apoptosis pathway that cannot be blocked by AIP mutants or r*Aip* siRNA. (One-way ANOVA with Tukey’s multiple comparison test correction A, J left; Two-ways ANOVA with Sidak’s multiple comparison test correction B-C-G-H-I-J right. **, *p* < 0.01; ***, *p* < 0.001; ns non-significant).

We transfected wt or variant *AIP* plasmids into GH4C1 cells in the absence or presence of RET_S_ and evaluated apoptosis by Hoechst 33258 staining, a system model in which we had extensive previous experience as an indicator of the activity of the RET-associated apoptotic pathway [2, 3] (Fig. 2A, B and Supplementary Figure 2A). Apoptosis was confirmed by a caspase-based fluorescent cellular assay and cell numbers (Supplementary Figure 2A–E).

The basal apoptosis rate in the absence of RET was not affected by any of the AIP variants, except the V49M variant that doubled the basal apoptosis rate (Fig. 2A). This germline variant was identified in a patient with gigantism but no loss-of-heterozygosity was detected in the somatotrophinoma tissue [37].

RET_S_ transfection enhanced more than four times the rate of apoptosis in GH4C1 cells with or without wtAIP co-transfection (Fig. 2B). Apoptosis was prevented by GDNF, indicating that expression of wtAIP did not alter GDNF-induced RET activation and survival.

However, when some of the variant *AIP* plasmids were co-transfected with RET_S_, apoptosis was strongly and significantly reduced in the absence of GDNF (Fig. 2B). Similar results were obtained for RET_L_ (Fig. 2B).

*AIP* variants R16H, V49M, C238Y, I257V, R271W, R304Q, R325Q prevented RET-dependent apoptosis, while R188Q and E245K did not alter RET-induced apoptosis (Fig. 2B). These two variants were actually found in patients with sporadic microprolactinoma and sporadic macroprolactinoma, therefore not the most typical characteristics associated with *AIP* mutations (Supplementary Table 1), had normal half-life and were considered non-pathogenic in the majority of algorithms in our *in silico* assessment (Supplementary Table 2). Of all the *in silico* algorithms, the most recent CADD 1.6 classification of the *AIP* variants coincided with the results from the apoptosis assay in pituitary cells (Fig. 2B and Supplementary Table 2). Based on these data, we divided the variants we tested into ‘our pathogenic’ (R16H, V49M, C238Y, I257V, R271W, R304Q, R325Q) and ‘our benign’ variants (R188Q and E245K) for further experiments.

Our results in somatotrophs show inhibition of RET-induced apoptosis by mutated AIP, while a previous study in HEK293 found no effect [34]. Therefore, we repeated our experiments in HEK293 and found no inhibition on RET-induced apoptosis in our pathogenic variants (Fig. 2C), confirming the previous data. These results indicate that the effect of variant *AIP* was specific for pituitary cells expressing *Pit1*. While PIT1 plays a key role in the RET-related apoptotic pathway (Fig. 2D) [2, 3, 30], we considered that in somatotroph cells, wtAIP could be either implicated in promoting the RET-apoptotic pathway or in blocking the RET-survival pathway. Pathogenic *AIP* variants could either lack apoptosis-supporting activity of wtAIP or promote the RET-survival pathway even in the absence of GDNF.

To explore these questions, we studied PIT1/ARF/p53 expression (Fig. 2E). Cells co-transfected with RET and wtAIP increased PIT1, ARF and p53 expression. RET co-transfected with any *AIP* variant that blocked apoptosis (R16H, V49M, C238Y, I257V, R271W, R304Q, R325Q) did not present stimulated PIT1 expression and consequently had no ARF or p53 increase. *AIP* variants that did not block RET-induced apoptosis (R188Q, E245K) showed increased PIT1, ARF and p53 expression, similar to wtAIP (Fig. 2E). To confirm again the importance of the cell type, we repeated these experiments in HEK293 cells: here RET expression induces ARF and p53 expression and the presence of wtAIP or variant AIP does not change this induction (Fig. 2F).

Next we studied the functional effect of endogenous *Aip* downregulation in GH4C1 cells. After GDNF deprivation, RET_S_ induced apoptosis in the presence of scrambled (Sc) siRNA corresponding to increase in the expression of PIT1, ARF and p53 (Fig. 2G). *Aip* siRNA blocked RET-induced apoptosis in correlation with repressed PIT1, ARF and p53 levels.

A more physiological model was tested for the pituitary-specific effect of AIP. Primary pituitary cultures are a mix of cells with around 50% somatotroph cells. We transfected empty vector, wtAIP or three AIP variants (V49M, R304Q, R325Q) and tested the endogenous RET-apoptotic pathway (Fig. 2H). After GDNF deprivation, 50% of the cells had died and this was not altered by exogenous human wtAIP since PIT1, ARF and p53 were induced. AIP variants blocked apoptosis as efficiently as exogenous GDNF, since they blocked PIT1 induction and consequently, ARF and p53 protein levels were low (Fig. 2H). In primary pituitary cultures, repression of endogenous *Aip* with siRNA blocked the endogenous RET-apoptotic pathway similar to GDNF, with reduced expression of PIT1, ARF and p53 levels (Fig. 2I).

Finally, to test which stage of the RET pathway is influenced by AIP, we transfected GH4C1 with intracellular (IC)-RET instead of full-length RET_S_ together with wtAIP, variant AIP (V49M, R304Q) or *Aip* siRNA (Fig. 2J). In the presence of the 707-1017 intracellular portion of monomeric RET (IC-RET), AIP variants or *Aip* siRNA did not present any anti-apoptotic effect and PIT1/ARF/p53 were fully induced as in wtAIP. This suggests that AIP could be involved in the generation of the intracellular RET fragment. The RET-induced mRNA expression of the two key regulated genes *Pit1* and *Arf* is stimulated by wtAIP and inhibited by *AIP* variants or *Aip* siRNA (Supplementary Figure 3A–G). This indicated that AIP acts on the RET-apoptotic pathway through a mechanism upstream of *Pit1* induction. We investigated the first step in the pathway. In cells transfected with RET_S_ + wtAIP, 1 hour after GDNF deprivation RET could be detected as a full-length double band of 170 kDa plus a strong band of around 42 kDa corresponding to one C-terminal tail cut by caspase-3 at position 707 (707-1072) and a smaller and weaker band ≤40 kDa corresponding to IC-RET, cut twice by caspase-3 (positions 707-1017) (Supplementary Figure 3H). These data identified a novel mechanism and provide an explanation for the observed reduced apoptosis: *AIP* variants prevented RET processing and IC-RET generation (Supplementary Figure 3H). On the other hand, *AIP* variants did not activate the survival pathway since no differences were found in AKT phosphorylation (Supplementary Figure 3H).

### AIP is essential for the initiation of the RET-apoptotic pathway: effect on caspase-3 and PKCδ

To investigate the role of AIP in the early initiation of the RET-apoptotic pathway, we standardized subcellular extracts characterized in Supplementary Figure 4.

**Fig. 3:**
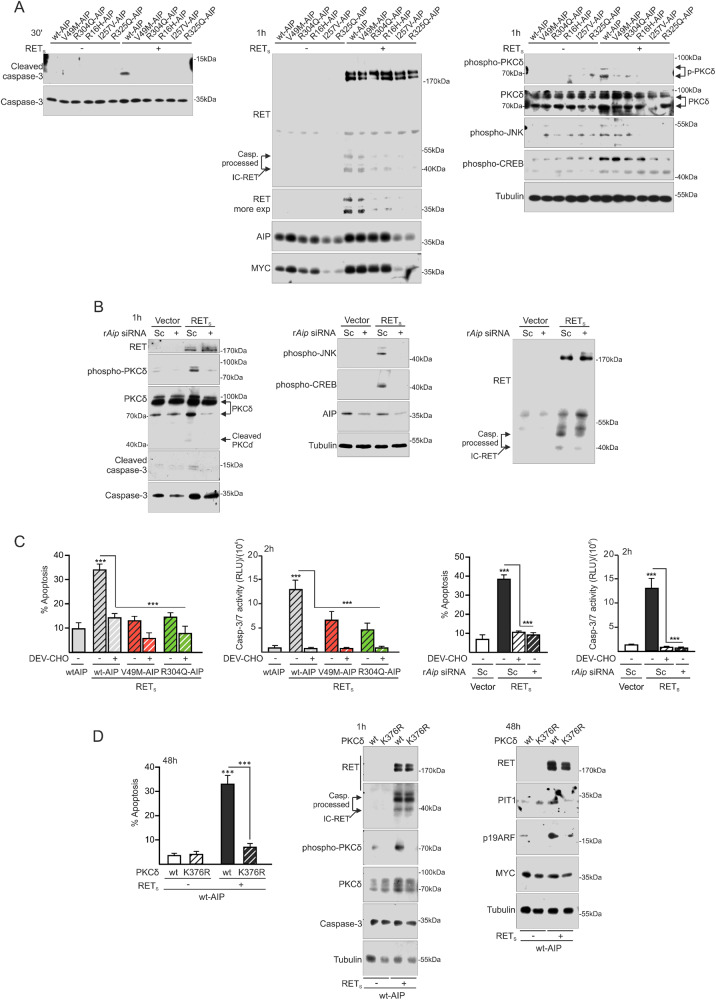
*AIP* variant or *Aip* repression alter the RET-apoptotic pathway at an early step: inhibition of caspase-3 and PKCδ activation. **A** Plasma membrane fractioning extracts 30 min after low serum conditions show that variant AIP block early caspase-3 cleavage and activation. At 1 h, intracellular IC-RET processing is downregulated in the presence of variant AIP. p-PKCδ is concordantly downregulated. p-JNK is also blocked. This time is too early to see any consistent change in p-CREB, previously described after 24 hours of GDNF deprivation. **B** r*Aip* siRNA blocks caspase-3 cleavage and activation together with p-PKCδ induction. In this case, p-JNK and p-CREB increases are also blocked by *rAip* siRNA, indicating different time-course than with variant AIP transfection. Caspase-3 processed IC-RET bands are downregulated in the presence of r*Aip* siRNA. **C** Quantification of caspase activity shows that caspase-3 activation is an early event after GDNF deprivation that can be blocked by the caspase-3 peptide inhibitor DEV-CHO or variant AIP or r*Aip* siRNA in a similar fashion. This corresponds well with the later detected apoptosis. **D** Apoptosis is absolutely dependent on PKCδ activity since the kinase-dead mutant K376R PKCδ blocks RET with wt*AIP* dependent apoptosis. Kinase-dead PKCδ does not alter IC-RET processing by caspase-3. (Two-ways ANOVA with Sidak’s multiple comparison test correction C-D. ***, *p* < 0.01).

We selected fraction 1, enriched in plasma membrane proteins, for further experiments comparing all *AIP* variants (Fig. 3). Cleaved caspase-3 was undetectable in the presence of RET_S_ with any variant in comparison to RET_S_ with wt*AIP* (Fig. 3A). Similar results were obtained for IC-RET or p-PKCδ, detected only in RETs with wt*AIP* (Fig. 3A). Total PKCδ presented two bands since it is also a substrate of caspase-3. The lower band (processed PKCδ) was high in RET_S_ with wt*AIP* and reduced with all *AIP* (pathogenic) variants. p-JNK or p-CREB did not present a consistent pattern at this time, but they are late proteins in the pathway and needed a longer time course. Cells transfected with RET_S_ and *Aip* siRNA presented no evidence of cleaved caspase-3, IC-RET, p-PKCδ, p-JNK or p-CREB compared to RET_S_ with control Sc siRNA (Fig. 3B).

Measuring caspase-3 activity at 1 hour showed that variant *AIP* or *Aip* siRNA blocked RET-induced caspase-3 activity corroborating the consequent effects on apoptosis (Fig. 3C).

To demonstrate that PKCδ activation was essential in the pathway, we transfected a PKCδ kinase-dead point mutant, K376R (Fig. 3D). This mutant was able to block PIT1/ARF increase and apoptosis in cells transfected with RET_S_ and wtAIP. One hour after deprivation, PKCδ-K736R did not present RET-induced PKCδ phosphorylation, but did not affect IC-RET processing (Fig. 3D).

### Interactions between RET, caspase-3, PKCδ and AIP: effects of AIP variants

**Fig. 4:**
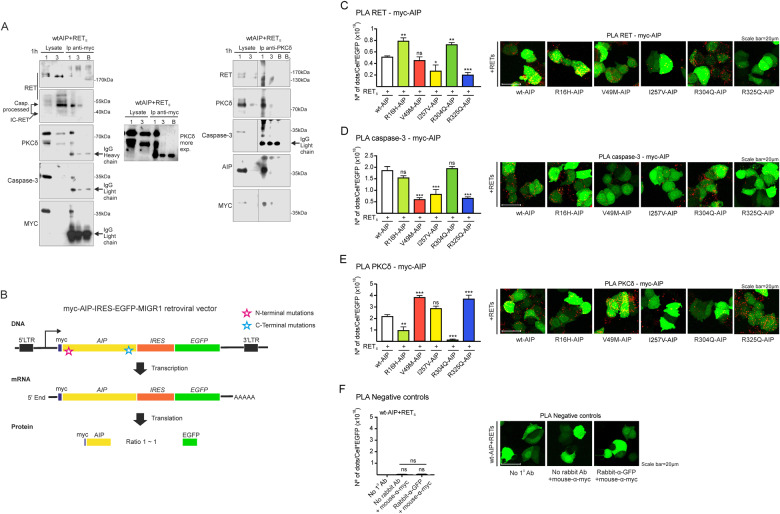
Molecular interactions between AIP, RET, PKCδ and caspase-3 are altered by either N-term or C-term AIP point mutations. **A** wtAIP (MYC-tagged) binds with RET, PKCδ and caspase-3 at the cytoplasm as shown by anti-MYC immunoprecipitation of plasma membrane/cytoplasm extracts (1) and nuclear extracts (3), obtained 1 hour after serum deprivation. As control, a blank (B) immunoprecipitation with antibody and beads without cell extracts. Left: RET, IC-RET, caspase-3, PKCδ and AIP are co-immunoprecipitated with anti-MYC-tag antibody. Right: RET, caspase-3, AIP and PKCδ are co-immunoprecipitated with PKCδ antibody. In both types of immunoprecipitations, a very slight band of PKCδ or MYC-Aip can be detected in the nuclear fraction. **B** Cartoon showing the constructs designed for the quantitative study of the interaction of wtAIP or each AIP variant with RET, caspase-3 and PKCδ through PLA assay. Each construct expressed similar amounts of AIP and EGFP, detected with GFP antibody and used as reference value in each cell. **C**-**F** PLA assays 1 hour after deprivation. **C** PLA assay between RET and AIP (MYC-AIP). I257V and C-terminal R325Q variant significantly reduces the interaction: representative confocal microphotographs and quantification (*n* = 6 per AIP variant). **D** PLA assay between caspase-3 and AIP. Both N-terminal V49M and C-terminal R325Q variants, together with I257V, significantly reduce the interaction. **E** PLA assay between PKCδ and AIP. Both N-term R16H and R304A variants significantly reduce the interaction, while V49M and R325Q enhanced the interaction, these are the residues that interact with caspase-3. I257V does not alter the interaction. (One-way ANOVA with Holm-Sidak’s multiple comparison test correction C; Kruskal-Wallis with Holm–Sidak’s D, Dunn’s multiple comparison test correction (E, F). **, *p* < 0.01; ***, *p* < 0.001; ns non-significant).

RET (and IC-RET), caspase-3 and PKCδ form a complex [2]. AIP has been demonstrated to bind to RET in HEK293 cells and in whole pituitary extracts [34]. To determine if AIP was able to bind all the proteins in this complex in somatotrophs, we transfected cells with the MYC-tagged wtAIP and RET and immunoprecipitated AIP with anti-MYC tag antibody (Fig. 4A left). Full-length caspase-3, both bands of PKCδ, and RET were easily detected in these pulldowns. For RET, the major band was IC-RET, although full-length RET could be also detected. When anti-PKCδ was used for immunoprecipitation, AIP (both with anti-MYC and anti-AIP), caspase-3 and RET were also detected (Fig. 4A right).

To dissect the molecular interactions between the four proteins through the AIP variants, we cloned all MYC-tagged AIPs in the MIGR1 vector containing the IRES motive followed by EGFP (Fig. 4B). In this way, for each AIP protein molecule translated, there will be approximately one molecule of EGFP. We used this system to equalize for protein expression of each variant in transfected cells. Following this, we performed a Proximity Ligation Assay (PLA) to explore direct interaction (<40 nm) of AIP with these three proteins [38, 39]. Using anti-MYC antibody (mouse origin) and anti-RET, anti-caspase-3 or anti-PKCδ antibodies (rabbit origin) we quantified the spots after normalization by EGFP expression. The RET-AIP PLA assay determined that R325Q *AIP* variant abolished, and I257V reduced, the interaction while R16H and R304Q enhanced the interaction (Fig. 4C). The caspase-3-AIP PLA assay determined that V49M, I257V and R325Q *AIP* variants abolished interaction (Fig. 4D). The PKCδ-AIP PLA assay determined that R16H and R304Q variants abolished the interaction while V49M and R325Q enhanced the interaction (Fig. 4E). The GFP-AIP PLA assay as well as the absence of primary antibody did not show any interaction (Fig. 4F).

A 3D model [40, 41] based on a monomer [42, 43] explained our PLA assay results (Extended text and Supplementary Figure 5). Based on these data, lack of AIP or presence of our pathogenic AIP variants may disrupt the RET/caspase-3/PKCδ/AIP complex resulting in reduced JNK activation and reduced apoptosis.

### AIP variants expressed in the pituitary affects postnatal growth in vivo

We wanted to test *AIP* point mutations individually in relation to the RET pathway in a high-throughput way without having to generate a new genetically modified mouse for each mutation. In the first week of life, rodent pituitary undergoes somatotroph cell expansion with active cell proliferation of the stem population and quick differentiation into somatotroph cells [44, 45]. Retroviruses specifically infect actively proliferating cells [46]. We incorporated into the viral injection the two key neuroendocrine hormones driving somatotroph proliferation, GHRH and ghrelin, to reinforce somatotroph-specific infection (Supplementary Methods). Following optimalisation, we developed a new model to inject the pituitary through the ear canal immediately after birth, allowing early testing of gene variants.

**Fig. 5:**
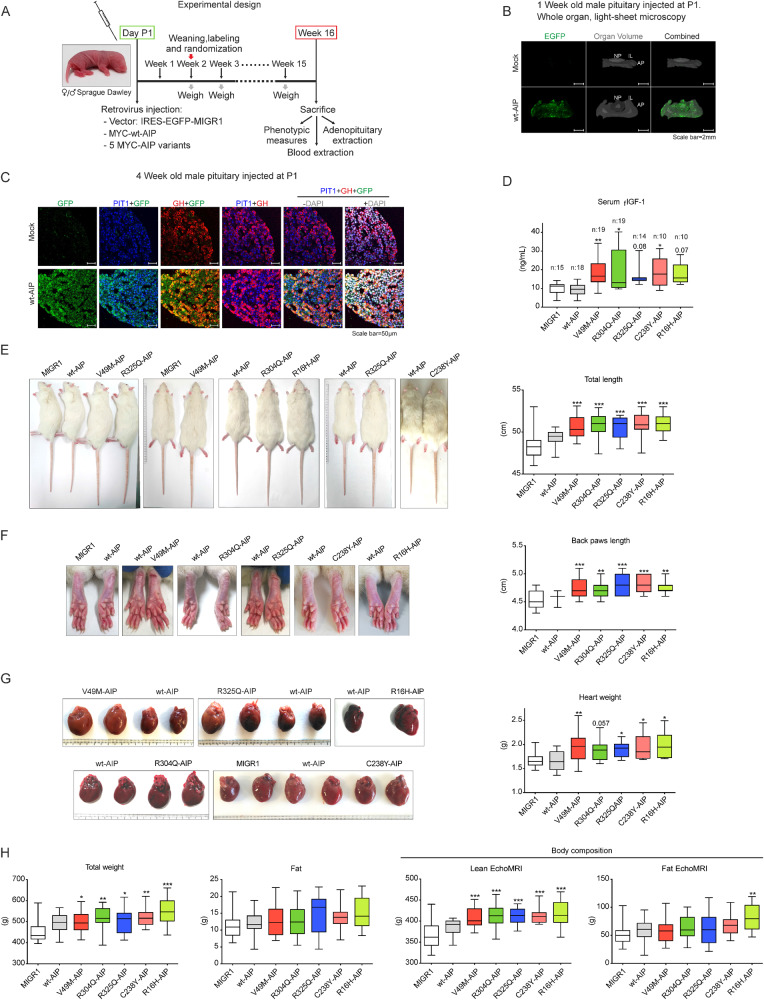
AIP pathogenic mutations injected in the pituitary at birth and expressed in somatotrophs cause acro-gigantism in male rats. **A** Chronogram of the model used to test the AIP variants. Retrovirus bearing the empty vector, or expressing wtAIP, or five different AIP variants (V49M, R304Q, R325Q, C238Y, R16H), followed by an IRES-EGFP expressing element were injected at the pituitary in postnatal day P1. Two weeks later, animals were randomized. Every week animals were weighed. Some animals were sacrificed 1 or 4 weeks after injection to study the pituitary gland, while others at week16, when biometric data, together with serum and organ weights, were collected. **B** 3D whole organ pituitaries clarified with X-Clarity and studied with light-sheet microscopy one week after the infection of mock virus or wtAIP-IRES-EGFP virus. Grey: whole organ volume (mock: coronal position respect to light-sheet; wtAIP: axial position respect to light-sheet). EGFP fluorescence is observed exclusively in the pituitary injected with wtAIP retrovirus throughout the whole organ. AP adenopituitary or adenohypophysis, IL intermediate lobe, N neuropituitary. **C** Somatotroph specificity was promoted by the use of retrovirus infecting the most proliferating cells at this period, that are the somatotrophs, and reinforced by combining the virus with GHRH and ghrelin neuropeptides inducing somatotroph proliferation. Paraffin sections of mock or wtAIP retrovirus infected four weeks old pituitaries showing confocal GFP (green) colocalization with PIT1 (nuclear, blue) and GH (red) in somatotroph cells. **D** Serum Free IGF-1 (_F_IGF-1) levels in the different groups of male rats showing that AIP variants but not wtAIP increased significantly and consistently with GH action at its primary target, the liver that secretes serum IGF-1. **E** Male rats infected with variant AIP were significantly longer, with longer tail (and longer body, shown in Supplementary Figure 7). **F** Soft tissues were enlarged in male animals bearing AIP variants in the pituitary. Significant differences were found for back and front paws and ears in rats injected with AIP variants. **G** AIP variants induced cardiomegaly. **H** Left: Total body weight was increased but total fat mass presented no differences. Right: Body composition was assessed by EchoMRI. AIP variants significantly increased lean mass, while fat mass was not affected except for R16H. (One-way ANOVA with Dunnett’s test multiple comparison test correction D-E-F-G-H. *, *p* < 0.05; **, *p* < 0.01; ***, *p* < 0.001; numbers, when *p* is non-significant but is small).

We prepared MIGR1 retrovirus bearing vector alone, wt*AIP*, all of our four stable (R16H, V49M, R304Q and R325Q) and one of our unstable (C238Y) pathogenic *AIP* variants, and validated them in vitro (Supplementary Figure 6A–C). The possibility that an unstable AIP variant had a functional effect had mixed odds. On the one hand, the used MIGR retroviral vector has a strong promoter. On the other hand, the rat pituitary cells conserved their own two endogenous normal *Aip* alleles. Based on CADD v1.6 scores (C238Y 26.9, R271W 24.7 (see Supplementary Table 2)) and our previous functional data [8, 14, 17], we selected this variant for in vivo study. Retroviruses were injected into male *Sprague-Dawley* rats at P1 (Fig. 5A). EGFP fluorescence was detected in the three pituitary lobes using 3D light-sheet microscopy one week after injection of wt*AIP* retrovirus (Fig. 5B). Somatotroph specificity of the infection was tested in through immunofluorescence colocalization with PIT1 and GH. Four weeks after injection of wtAIP retrovirus, triple immunofluorescence demonstrated massive co-localization of GH and EGFP (cytoplasmic) together with PIT1 (nuclear) (Fig. 5C).

The litter number was adjusted for each mother. Two weeks after injection, animals were weaned, identified, weighted, and randomized. Animals were weighed weekly and at week 16, animals were anesthetized, measured, and serum and tissue samples were taken (Fig. 5A). Measures have been previously demonstrated as bona fide markers of rodent growth and GH action [47–50]. At 16 weeks male animals injected with *AIP* variants showed significantly higher IGF-1 serum levels compared to wt*AIP* or MIGR1 vector alone injected animals (Fig. 5D). Variant *AIP*-injected male rats were longer in total length, tail length and body length (Fig. 5E and Supplementary Figure 6D), and showed significantly longer back paws, front paws, ears, head and incisors (Fig. 5F and Supplementary Figure 6D).

Excess GH results in cardiac hypertrophy in humans. Variant *AIP*-injected male rats presented significantly larger hearts (Fig. 5G). Pituitary weights had a tendency to be larger in variant *AIP* pituitaries reaching significance in two groups (Supplementary Figure 6D). Other organs, like the liver, did not show significant size change as expected (Supplementary Figure 6D). Patients with acromegaly show increased weight and lean body mass. Variant AIP- injected male rats presented significantly higher weights (Fig. 5H). When body composition was assessed using MRI, a significant increase in lean mass was observed in the male rats injected with *AIP* variants in comparison with wt*AIP* or MIGR1 vector (Fig. 5H).

It is known that each endocrine cell-type in the pituitary have a particular geographical distribution. Somatotrophs are concentrated in the lateral parts of the gland [51, 52]. In pituitaries of adult rats injected at birth, GFP cells were concentrated at the periphery of the pituitary lobes where somatotroph cells reside and co-expressed PIT1 (Supplementary Figure 6E). Next, we studied the pituitaries to determine if the phenotype induced by the variant *AIP* could be related in vivo to the RET-apoptotic pathway.

**Fig. 6:**
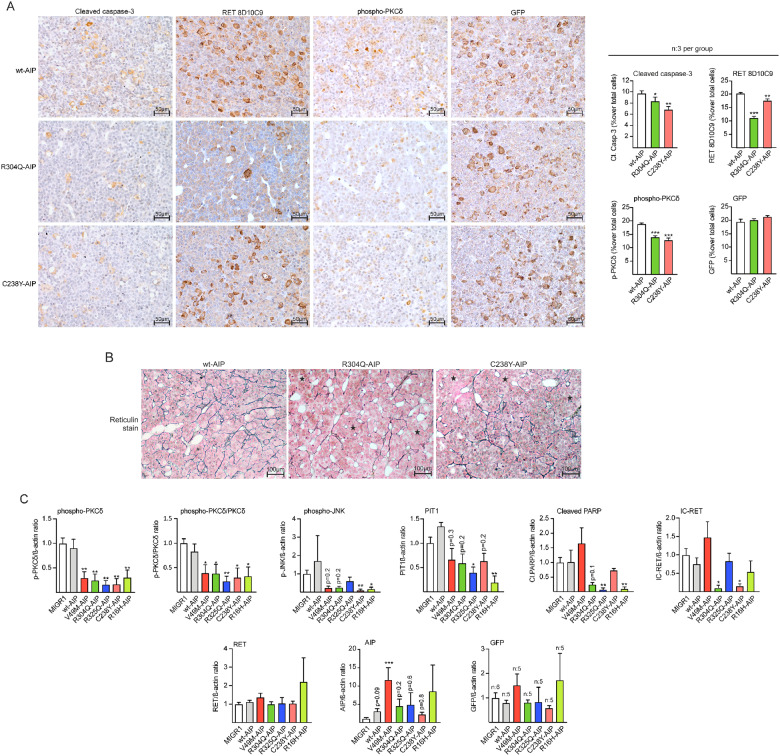
AIP pathogenic mutations injected in the pituitary at birth block the RET/caspase-3/PKCδ/PIT1 apoptosis pathway in male rats inducing hyperplasia. **A** Left: Microphotographs of immunohistochemistry for cleaved caspase-3, IC-RET (monoclonal RET8D10C9 antibody), p-PKCδ, and GFP (for EGFP detection) in sections of wt or variant *AIP* injected pituitaries. Nine sections from three independent animals per group were mounted in the same slide (20x magnification, bar = 50 μm). Right: Immunohistochemistry was quantified compared to total nuclei. While there were no significant differences in the % of GFP cells, the % of cleaved caspase-3, IC-RET and p-PKCδ stained cells was significantly reduced in variant AIP pituitaries respect to wtAIP pituitaries. **B** Reticulin staining in the same pituitaries as in (**A**). Partial disruption of the reticulin network with extended acini is observed (asterisks) in the variant AIP pituitaries. Pituitaries were hyperplastic (Supplementary Figure 7H). **C** Protein quantification respect to controls (loading control: beta-actin) of the western blot performed in groups (*n* = 4) of pituitary extracts from vector (MIGR1), wtAIP or variant AIP. High throughput gels for 22 samples were used to run extracts in parallel (Supplementary Figure 6F). Significant reductions in p-PKCδ and p-JNK were found. Cleaved PARP was also reduced in variant AIP pituitaries except for V49M. PIT1 and IC-RET were significantly reduced in some variants but in other results did not reach significance. Levels of RET, AIP and exogenous GFP were non-significant between groups. (Kruskall–Wallis with Dunn’s multiple comparison test correction, (**A**); One-way ANOVA with Dunnett’s multiple comparison test correction C. *, *p* < 0.05; **, *p* < 0.01; numbers, when *p* is non-significant but is small).

Male rats injected with variant *AIP* had a significantly lower percentage of cells positive for cleaved caspase-3, IC-RET and p-PKCδ (Fig. 6A) compared to wt*AIP*, while there was no difference in the percentage of GFP cells (EGFP expression) between the groups. The adenohypophysis area in the sections of the variant *AIP* injected male pituitaries was significantly larger than wt*AIP* (see below). Haematoxylin-eosin staining did not detect any adenomas, but reticulin staining was disrupted in *AIP* variant-injected pituitaries that showed pituitary hyperplasia with larger acini (Fig. 6B).

We prepared protein extracts from frozen pituitaries and run 20 parallel samples, four-five per condition (Supplementary Figure 6F). Global levels of p-PKCδ and relative p-PKCδ/total PKCδ were significantly reduced in variant *AIP*-injected animals in comparison with wt*AIP* or MIGR1 vector (Fig. 6C). p-JNK levels were also significantly reduced although some variant AIP groups did not reach significance (Fig. 6C). IC-RET levels were found to be significantly reduced in some variants although not in all (Fig. 6C). Cleaved PARP, a marker of active apoptosis in pituitary tissue [2] was significantly reduced in several groups of variant *AIP*-injected animals although not in all (Fig. 6C). PIT1 is a transcription factor common to somatotroph, lactotroph and thyrotroph cells. Although PIT1 protein detection by western blot was not specific for the somatotroph fraction, PIT1 protein levels were significantly reduced in two variants (R16H and R325Q) and reached *p* = 0.2 in the other three (V49M, R304Q and C238Y) (Data not shown). AIP protein expression had a tendency to be higher in pituitaries expressing human exogenous AIP (including wtAIP), but only reached significance in the V49M group. MYC expression, on the other hand, was only present in the rats infected with (MYC-)AIP-expressing virus at around 35 kDa (Data not shown). This MYC band was abundant in variant AIP pituitaries compared to wtAIP. No significant differences were found for full-length RET or GFP.

The results gathered with our in vivo model in male rats confirm the data found in vitro in GH4C1 cells and in primary pituitary cultures, demonstrating the role of AIP in the RET-apoptotic pathway. Moreover, the data in vivo confirmed the role of *AIP* variants implicated in *AIP*-FIPA blocking this pathway.

Results in female rats showed an attenuated phenotype (Extended text and Supplementary Fig. 7), although significant hyperplasia was present in both sexes in variant AIP pituitaries (Supplementary Figure 7H).

**Fig. 7:**
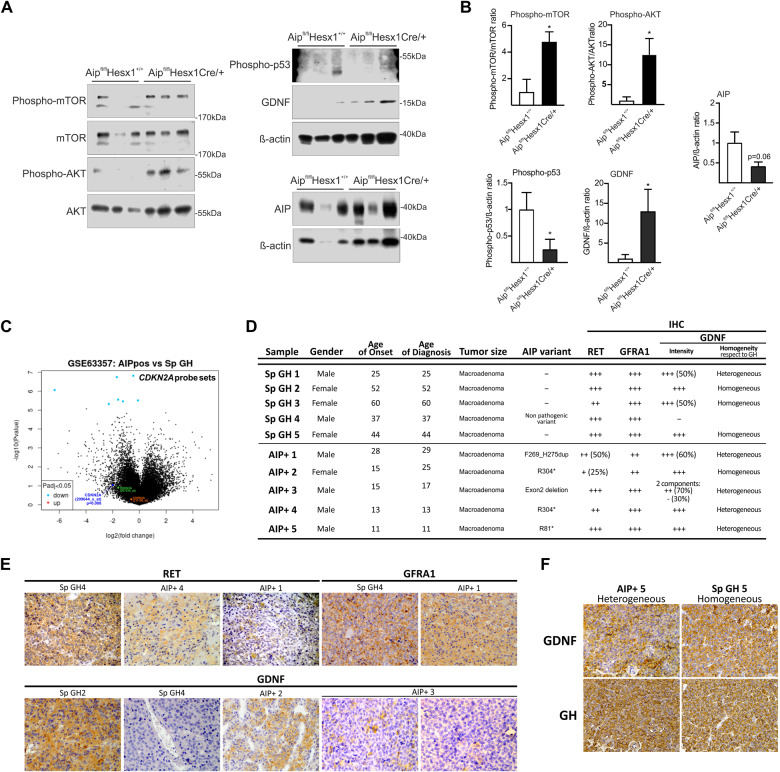
Pituitary adenomas from pituitary-specific *Aip*-knockout mice or from human patients have overexpression of the RET/GDNF survival pathway. **A** Pituitary extracts from15-month old *Aip*^*Flox/Flox*^;*Hesx1*^*Cre/+*^ knockout mice compared to *Aip*^*Flox/Flox*^*;Hesx1*^*+/+*^ control male mice. **B** Increased levels of GDNF, phospho-AKT and phospho-mTOR and reduced expression of phospho-p53 and AIP are found in mutant animals. **C** Expression of the *CDKN2A* probe set, including three probes, in *AIP*-FIPA pituitary adenomas compared to sporadic pituitary adenomas, retrieved from ReGEO GSE63357, a study using the Affymetrix HG-U133 Plus 2.0 array. Only 209644_x_at includes oligos specific for the *CDKN2A-ARF* specific Exon 1, while the other probes contain oligos specific for the other protein isoform *CDKN2A-p16* specific Exon 1 (21156_at), or the common Exon 3 (207039_at). **D** Immunohistochemistry comparing somatotroph macroadenomas from sporadic (Sp GH) or *AIP*-FIPA tumours (AIP+). **E** Representative stainings for RET (total RET HPA008356), GDNF and GFRα1. Sections were counterstained with haematoxylin. **F** Corresponding fields within the same sample, showing heterogeneity of GDNF staining compared to GH. (*N* = 3; Mann–Whitney, *p < 0.05; numbers, when p is non-significant but approximates to significant levels).

Recently, we have shown that human somatotroph sporadic adenomas without germline *AIP* mutation have an activated RET-GDNF survival pathway, with high GDNF levels [30]. We studied a second in vivo model, a pituitary-specific homozygous *Aip* knockout and explored the RET pathways in the pituitary tissues. 15 months old *Aip*^*Flox/Flox*^*;Hesx1*^*Cre/+*^ animals have a homozygous *Aip* deletion in the pituitary-derived epithelium and develop GH-secreting pituitary tumours [27]. Protein extracts from male *Aip-*knockout pituitaries compared to *Aip*^*Flox/Flox*^*;Hesx1*^*+/+*^ show a significant increase in GDNF and phospho-AKT/total AKT and phospho-mTOR/total mTOR ratios and reduced phospho-p53 expression (Fig. 7A, B). Total AIP protein levels were reduced but not totally absent, since only the endocrine epithelium expressing *Hesx1* is affected (Fig. 7A (right)-B) [53, 54]. These results indicate that *Aip*-knockout pituitary adenomas in mice have an upregulated RET-GDNF/survival pathway, similar to human sporadic somatotroph adenomas, and also decreased apoptosis.

To explore if human *AIP*-FIPA somatotroph adenomas also conserved the RET-GDNF pathway, we performed studies in two directions. Results based on Affymetrix probes from GSE63357 [55] did not found differences in *RET*, *GDNF*, *GFRA1*, *PIT1* or *TP53* RNA expression between sporadic and *AIP*-FIPA pituitary adenomas (Extended text and Supplementary Fig. 8A–D and E). The *CDKN2A* gene (coding for p19ARF and for p16) was represented by three probes. We found reduction of expression in *AIP*-FIPA tumours compared to sporadic adenomas for probe 209644_x_at directed to the Exon 1 of the p19ARF isoform (Fig. 7C and Supplementary Fig. 8F, G), while no difference was found for probe 211156_at directed to the Exon 1 of the p16 isoform or for probe 207039_at directed to the common Exon 3 for both isoforms. Furthermore, recently we have generated novel unpublished data using RT-qPCR with TaqMan probes where ARF expression is low in AIP+ human tumour vs controls (Alvarez and co-workers, unpublished). Our previous data showed that high ARF mRNA level (relative expression >0.1) in the tumour corresponds to a good response to somatostatin analogues, while low ARF expression (<0.1) indicated resistance to somatostatin analogues [30]. The AIP+ tumour present ARF levels around 0.01 compared to 44 sporadic somatotrophinomas responsive to somatostatin analogues, grouping with 13 unresponsive sporadic somatotrophinomas (Alvarez and co-workers, unpublished). At the protein level, no suitable antibody for immunohistochemistry specific for ARF is available. We performed immunohistochemistry for RET (total RET, HPA008356), GDNF and GFRα1 comparing five sporadic (Sp GH) and five AIP+ somatotroph adenomas (Fig. 7D, E). No difference was seen between sporadic and AIP+ samples for RET, GFRα1 and GDNF regarding intensity of the staining. However, comparing corresponding areas on consecutive sections stained for GH and for GDNF, we found more heterogeneous staining of GDNF in AIP+ tumours with negative populations interspersed with the stained cells in some samples (Fig. 7D, F). The relatively lower GDNF would argue that a less stimulatory pathway is needed in these samples to compensate for the already downregulated apoptotic pathway, and GDNF would be useful exclusively to promote growth. This could be explored in future studies.

## Discussion

We identified that AIP is needed for the normal functioning of the RET/Pit1/ARF/p53-apoptosis pathway (Graphical Abstract, Supplementary Fig. 9). This is disrupted by *Aip* knockdown, or with overexpression of deleterious *AIP* variants. While the pathogenic role of truncating *AIP* variants is established, there are controversies with some missense variants, similar to all other fields of clinical genetics. The data from our *in silico* assessment of *AIP* variants using CADD v1.6 corresponded to the blocking effect on the RET-apoptotic pathway (R16H, V49M, C238Y, I257V, R271W, R304Q and R325Q). Two other missense AIP variants, R188Q and E245K (considered as benign in CADD v1.6) do not affect the RET-apoptotic pathway, suggesting that these are non-pathogenic variants [56, 57]. We note that three from the variants showing disruption of the RET apoptosis pathway in our studies here (R16H, R304Q and R325Q) have been suggested in other studies (for example, [58], and including our own), to be Variants of Uncertain Significance or possibly likely benign variants, while data here suggest some functional shortcomings. Many other studies suggest a clinical effect (revised in Supplementary Table 1 for each variant). This is a controversy occurring increasingly in clinical and experimental genetic studies probably due to differing experimental models and genetic background of patients. Further clinical and experimental experience may help to resolve these issues in the future. In our in vitro and in vivo models, deleterious *AIP* variants behave as dominant-positive, contravening the RET pathway despite the two normal endogenous *rAip* alleles. We might speculate that *AIP*-FIPA could be along the spectrum of gene–dosage mechanism of tumour suppressors, as has been suggested for other tumour suppressors [59]. In carrier subjects, mechanisms of regulation of the expression of the deleterious inherited variant (methylation, lncRNA, microRNA or other) dependent on each individual could modulate the penetrance of the disease, i.e. the appearance of the pituitary tumour.

RET had initially been proposed as a ‘dependence receptor’ in peripheral autonomic neurons as a mechanism explaining Hirschprung’s disease [32]. Apoptotic pathways associated with dependence receptors are often tissue-specific and frequently implicate enzymatic intracellular cleavage of the monomeric receptor by caspases or other enzymes, with different downstream apoptotic pathways [33]. Why do tissues need a dependence pathway? Dependence receptors have been referred to as “guardians of tissue homeostasis and cancer prevention” [33], since their pro-death activity observed upon ligand limitation is implicated during embryonic development. Abnormal inhibition of the pro-death activity of these receptors is associated with tumour progression.

The discovery of the postnatal pituitary stem cell niche expressing RET and GFRα co-receptors [44] led to the knowledge that new progenitors are constantly being recruited and differentiated into secretory endocrine cells in the pituitary [60, 61]. The mechanism of postnatal differentiation of somatotroph cells is not fully understood yet [62]. There is a slow cell turnover from a PIT-negative progenitor to a PIT1 positive somatotroph. Both too much PIT1 or too little would lead to abnormalities. The *Ret*-knockout mouse presents a larger pituitary gland with enhanced number of PIT1+ and GH+ cells [2]. This mouse model dies postnatally due to the absence of parasympathetic innervation in the bowels [63], so the postnatal endocrine phenotype cannot be elucidated.

While the RET-related pituitary studies were concentrating on somatotrophs [2, 3], RET expression has been demonstrated in rat lactotroph cells in lactating dams, when weaning is finished and there is a need for reduction of the lactotroph population through apoptosis [64]. Moreover, an in vivo model of lactotroph hyperplasia expressing RET showed a similar dependence-receptor pathway as in the somatotrophs [2]. This suggests that, although somatotrophs are the main population related to RET functionality, lactotrophs also rely on this pathway for apoptosis, therefore disruption may lead to lactotroph tumours; indeed, the first pure prolactinoma *AIP*-FIPA family has been recently described [65].

From the molecular point of view, AIP´s role is at the initiation of the RET-apoptotic pathway. AIP is directly associated with the quadrimeric complex formed by the intracellular portion of monomeric RET, caspase-3 and PKCδ, as shown by our PLA data. Functional PLA assays also helped us to assess the effect of missense *AIP* variants in the formation of this complex. The I257V and R325Q variant impinged on RET binding, three variants blocked caspase-3 binding (V49M, I257V, R325Q) and two impinged on PKCδ binding (R16H, R304Q). Variants that alter caspase-3 binding increased association to PKCδ (V49M, I257V, R325Q); variants that alter PKCδ binding increased association to RET (R16H, R304Q). In the absence of AIP, the complex could not be formed at all. The exploratory *in silico* molecular model suggests AIP acting as a monomer, as have been previously suggested [43]. This three-dimensional model fits with the functional data and the PLA assay. Our data also suggest that caspase-3 activation and IC-RET processing is preceding PKCδ activation, since a kinase-dead PKCδ also blocks the pathway but does not prevent IC-RET processing. These positions AIP at the cytoplasmic aspect of the plasma membrane, representing a link between RET and the caspase-3-PKCδ complex, as the key link between these enzymes and RET. PKCδ is an enzyme strongly implicated in differentiation/apoptosis in various human cell types [66, 67]. PKCδ is first phosphorylated followed by proteolytic cleavage by caspases to obtain the pro-apoptotic catalytic fragment [66]. The main caspase implicated in this action is caspase-3 [66].

Two models are proposed for apoptotic caspase-3 activity initiation, either an auto-regulatory cis/trans activation induced by aggregation / co-localization of protomers; or that once aggregation / co-localization of protomers is initiated the already present low amount of non-apoptotic active caspase-3 present in the cell is enough to initiate the cascade of auto-activation [68]. For caspase-3 activation in pituitary somatotrophs two conditions are required: presence of RET and absence of GDNF (i.e. low serum). RET transfection or empty vector transfection per se do not activate caspase-3 activity in somatotrophs in either of the two models we used ((i) GH4C1 cell line with exogenous RET transfection or (ii) somatotroph primary cultures having endogenous RET expression) (Fig. 2B, G, H, I). Our previous work has demonstrated that the RET point-mutant at D707N binds to caspase-3 but is unable to induce caspase-3 activity after deprivation of GDNF; correspondingly, caspase-3 is unable to process RET D707N [2]. Moreover, RET D707N is unable to bind PKCδ. On the other hand, the C-terminal RET 707-1017 fragment (IC-RET), contained between the two caspase-3 consensus sites in the cytoplasmic RET tail, induces apoptosis independently of the presence of any AIP mutant or rat Aip siRNA (Fig. 2J). This leads to the conclusion that RET processing by caspase-3 is the key AIP-related step in the RET-apoptosis pathway. We note that a kinase-domain point-mutant inactive PKCδ also prevents RET-induced apoptosis (Fig. 3A).

Our data here regarding the essential role of AIP, as a key member of the RET-caspase-3 complex, indicates a model where a full-length RET monomer+ caspase-3+ AIP+ PKCδ complex assembles which would initiate (i) caspase-3 auto-activation followed by (ii) RET monomer processing into a shorter fragment and (iii) activation of PKCδ resulting in activation of the apoptotic PIT1/p53 pathway. The interaction of this pathway with the previously described pathways involving cAMP, G_alphai-2,_ phosphodiaesterases, mir34 as well as the interactions with multiple proteins related to the cellular skeleton are currently unclear.

The results in the male rats show the action of deleterious *AIP* variants inducing GH excess and resulting gigantism by alteration of the RET-apoptotic pathway. The phenotype was homogenous for all deleterious variants with high IGF-1 (the main GH target hormone in serum), enhanced length, weight, increase in muscle mass, and bigger hearts. At the molecular level in the pituitary, pathogenic AIP reduced caspase-3, IC-RET, PKCδ, and PARP cleavage and activation. Adenohypophysis areas and reticulin staining in pituitary tissue showed that altering the RET/apoptosis pathway induces somatotroph hyperplasia and this is enough for GH/ IGF-1 excess and gigantism, but not for generating an adenoma within our study timeframe. This is a key result that could be also important for pathogenic process in the patients. We could speculate that pathogenic *AIP* variants will induce a model of step-by-step tumorigenesis with the first hyperplasia and later adenomas, and indeed this is seen both in the animal model and humans. In all three published mouse models, such as the global *Aip* heterozygous or the conditional *Gh*-specific or *Hesx1*-specific *Aip*-knockouts, pituitary hyperplasia is present many weeks before the appearance of adenomas [24–27, 69, 70]. Pituitary hyperplasia in humans is not commonly found, as by the time of clinical presentation tumour development has usually already occurred. However, there are a few cases of pituitary hyperplasia described in humans [8] and it also has been demonstrated around the tumour [71, 72]. How germline pathogenic *AIP* variants lead to invasive somatotroph adenomas in patients is not known and we can only speculate. The RET/GDNF tyrosine-kinase survival pathway could generate tumour promoting proliferative events. A subsequent independent genetic event adding aggressiveness would fit the animal models’ and patients’ data.

The data in female rats show quantitative differences to males. Females also had a growth phenotype since they are bigger, and had bigger hyperplastic pituitaries. However, the endocrine phenotype as young adults is blunted since IGF-1 presented no differences at 16 weeks, and the pituitary tissue did not show differences in the RET pathway at that precise moment. It seems that puberty, earlier in females than in males, is a factor in the development of gigantism. Probably, this also happens in human patients since gigantism is more often diagnosed in men than in women, even in *AIP* mutated subjects, despite no differences in the incidence of acromegaly [12, 73], although other gender differences in pituitary physiology could also play a role.

15 month old *Aip*^*Flox/Flox*^;*Hesx1*^*Cre/+*^ mice have established pituitary adenomas that we have also studied. In relation to the RET-apoptotic pathway, phospho-p53 was reduced. Hyperactivation of the RET/GDNF-survival pathway was demonstrated with enhanced GDNF protein expression, and phospho-RET, phospho-AKT and phospho-mTOR activation. This pathway would induce proliferation over the basis of the absence of apoptosis. These results fit well with recent findings in a long series of human sporadic somatotrophinomas (non *AIP*-FIPA) that have demonstrated a six-time increase in *GDNF* expression over the normal pituitary, and activation of the RET/GDNF-survival pathway including AKT/mTOR activation and downregulation of p53/PARP activity [30]. In sporadic somatotroph adenomas, quantitative *ARF* mRNA expression indicating some remnants of a functional RET-apoptotic pathway, was a significant marker of good prognosis and response to first-line therapy (surgery, somatostatin agonists), while absence of *ARF* expression indicating exclusivity of RET/GDNF survival pathway was associated to resistance to first-line therapy [30]. In that work, small kinase inhibitors able to inhibit RET/GDNF-induced kinase activity were shown as good alternative new avenues for treating resistant sporadic adenomas. Our present results indicate that this approach with anti-RET kinase inhibitors could even be more successful for treating the FIPA somatotroph adenomas that are so aggressive and resistant to surgery and the usual medical therapy with agonists. Reduced levels of AIP protein have been associated with resistance to somatostatin analogues in sporadic acromegaly [74]. The human data we present here suggest that ARF expression levels are downregulated in *AIP*-FIPA tumours, indicating the reduced activity of the RET apoptosis pathway. Corresponding to our data on sporadic somatotroph tumours [30], this low *CDKN2A-ARF* RNA expression could also explain the well-described resistance of *AIP*-FIPA tumours to first-generation somatostatin analogues. Moreover, although GDNF was present in the tumours, its expression was heterogenous, revealing that a combination of lack of apoptosis (low ARF) and increased RET kinase activity (GDNF) is playing a dual role in AIP+ tumour samples.

In summary, we have found an explanation of the long-sought question regarding the tissue and cell-type specificity of AIP-related tumours. AIP plays an essential role in the initial steps of the RET-apoptotic pathway in PIT1 expressing cells. Lack of AIP or pathogenic mutations block this pathway promoting the RET-survival pathway characteristic of the pituitary somatotrophinomas. This connection between AIP and RET has implications for the aggressive pituitary adenomas observed in the majority of affected patients with *AIP* mutations and could be of future use in alternative therapeutic approaches.

## Material and methods

Extended material and methods are detailed in Supplementary information.

### In silico study of AIP variants

Human AIP variants were analysed using databases and predicting algorithms, classified by the given in situ threshold and the alternative threshold proposed in [75].

### In vitro experimental design, cell lines and rat adenohypophysis primary culture

The rat GH4C1 cell line and primary adenohypophysis cells were cultured as described [2, 3, 76]. Recombinant GDNF (Calbiochem) is obtained from Sf9 fly cell line, (>97%) and with very low endotoxin contain (LPS ≤ 1.0 EnU/μg).

### Apoptosis and Cell counting assays

Apoptosis detection was performed and counted 48 h after treatments as described [2, 3, 30, 31]. Earlier apoptosis was detected with caspase-3/7 CellEVENT (Thermo).

### Lysates, subcellular fractionation, western blot and immunoprecipitation

Experimental setup for lysates and immunoprecipitations is detailed in Supplementary Methods [2, 77]. Subcellular fractionation was performed as [78] with some modifications. For rat pituitary westerns we used Precast Criterion™ TGX™ (Biorad). Antibodies and dilutions are shown in Supplementary Information.

### Caspase-3 enzyme activity

Cells were lysed with 50 μL Caspase-Glo® 3/7 Assay kit (G8090, Promega) and measured with Multimode Mithras LB 940 (Berthold Technologies).

### Promoter activity

*Pit1* promoter activity was measured as described [2, 3, 76].

### RNA purification and qRT-PCR

It was performed as described [30]. Primers are listed in Supplementary Information.

### Proximity Ligation Assay (PLA)

We used the in situ technology Duolink® Proximity ligation Assay (PLA) [38, 39] (Sigma) followed by Fiji-Image J 1.51 [79] and CellProfiler 3.1.8 [80] software quantification.

### In silico exploratory model of protein interaction

An exploratory model of the interaction between AIP, RET_S_, PKCδ and caspase-3 based on the results of the PLA assay was carried out with two softwares SWISS-MODEL and UCSF Chimera [40, 41].

### In vivo rat model to functionally evaluate missense *AIP* variants in pituitary

Procedures were carried out under the Procedures Act n° 15003/14/005 (to CVA), granted by Galicia Regional Government, following NC3Rs’ ARRIVE guidelines. Pups were injected at P1. Hormones and phenotypic measures are detailed in Supplementary information.

### Adenohypophysis immunohistochemistry and immunofluorescence. Whole pituitary clarification with X-Clarity. Light-sheet microscopy

Immunohistochemistry in human pituitary adenomas was performed with EnVision as described [3]. Pituitary clearing was performed with X-CLARITY (Logos Biosystems) following by analysis with Imaris 9.3 software (Bitplane). Ethical approval for human somatotrophinomas stainings was obtained from Cambridgeshire 1 Research Ethics Committee, Cambridge, UK (06/Q0104/133).

### Pituitary-specific AIP knockout mouse model

Animal studies were performed following the NC3Rs’ ARRIVE guidelines under Animal Licence Permission No PPL 70/7665, supported by QMUL’s Ethics Committee. The two lines are derived from C57BL/6J (www.jax.org). [27, 54].

### Data collection and statistical analysis

Statistical analysis was performed using GraphPad Prism 8.0.1 (San Diego, USA), SPSS Statistics 20 (IBM, USA) and Corel Draw Graphic Suite 2017.

### Supplementary information

Supplementary Information

## Data Availability

All data generated or analysed during this study are included in this manuscript (and its supplementary information files).
