# Correction: RET signalling provides tumorigenic mechanism and tissue specificity for AIP-related somatotrophinomas

**DOI:** 10.1038/s41388-023-02862-9

**Published:** 2023-11-06

**Authors:** Angela R. Garcia-Rendueles, Miguel Chenlo, Fernando Oroz-Gonjar, Antonia Solomou, Anisha Mistry, Sayka Barry, Carles Gaston-Massuet, Montserrat Garcia-Lavandeira, Sihara Perez-Romero, Maria Suarez-Fariña, Alberto Pradilla-Dieste, Carlos Dieguez, Patrick Mehlen, Márta Korbonits, Clara V. Alvarez

**Affiliations:** 1 Neoplasia & Endocrine Differentiation P0L5, Centro de Investigación en Medicina Molecular y Enfermedades Crónicas (CIMUS), University of Santiago de Compostela (USC), Santiago de Compostela, Spain; 2 Department of Endocrinology, William Harvey Research Institute, Barts and London School of Medicine and Dentistry, Queen Mary University of London, London, UK; 3 Patrick Mehlen, Apoptosis, Cancer and Development Laboratory- Equipe labellisée ‘La Ligue’, LabEx DEVweCAN, Institut PLAsCAN, Centre de Recherche en Cancérologie de Lyon, INSERM U1052-CNRS UMR5286, Université de Lyon, Centre Léon Bérard, 69008 Lyon, France

Correction to: *Oncogene* 10.1038/s41388-021-02009-8, published online 29 September 2021

The authors have become aware of an error in Figure 6B, where the histological image assigned to R304Q was from the same animal as the image marked C238Y. The authors have provided the corrected figure below. This correction has no impact on the results or conclusions presented in this paper.
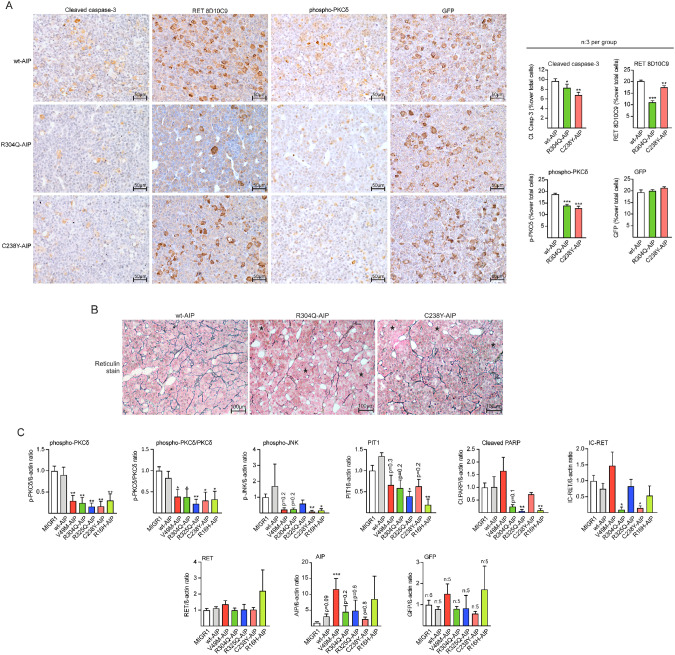


The authors apologize for the error and regret any inconvenience this may have caused for the readers.

The original article has been corrected.

