# Supplementary material for: RET signalling provides tumorigenic mechanism and tissue specificity for AIP-related somatotrophinomas
Source: Oncogene. 2021 Sep 29;40(45):6354–68. doi: 10.1038/s41388-021-02009-8 (PMC8585666; doi:10.1038/s41388-021-02009-8)
Supplement: Supplementary file 1 — Supplementary Information [file 41388_2021_2009_MOESM1_ESM.pdf]

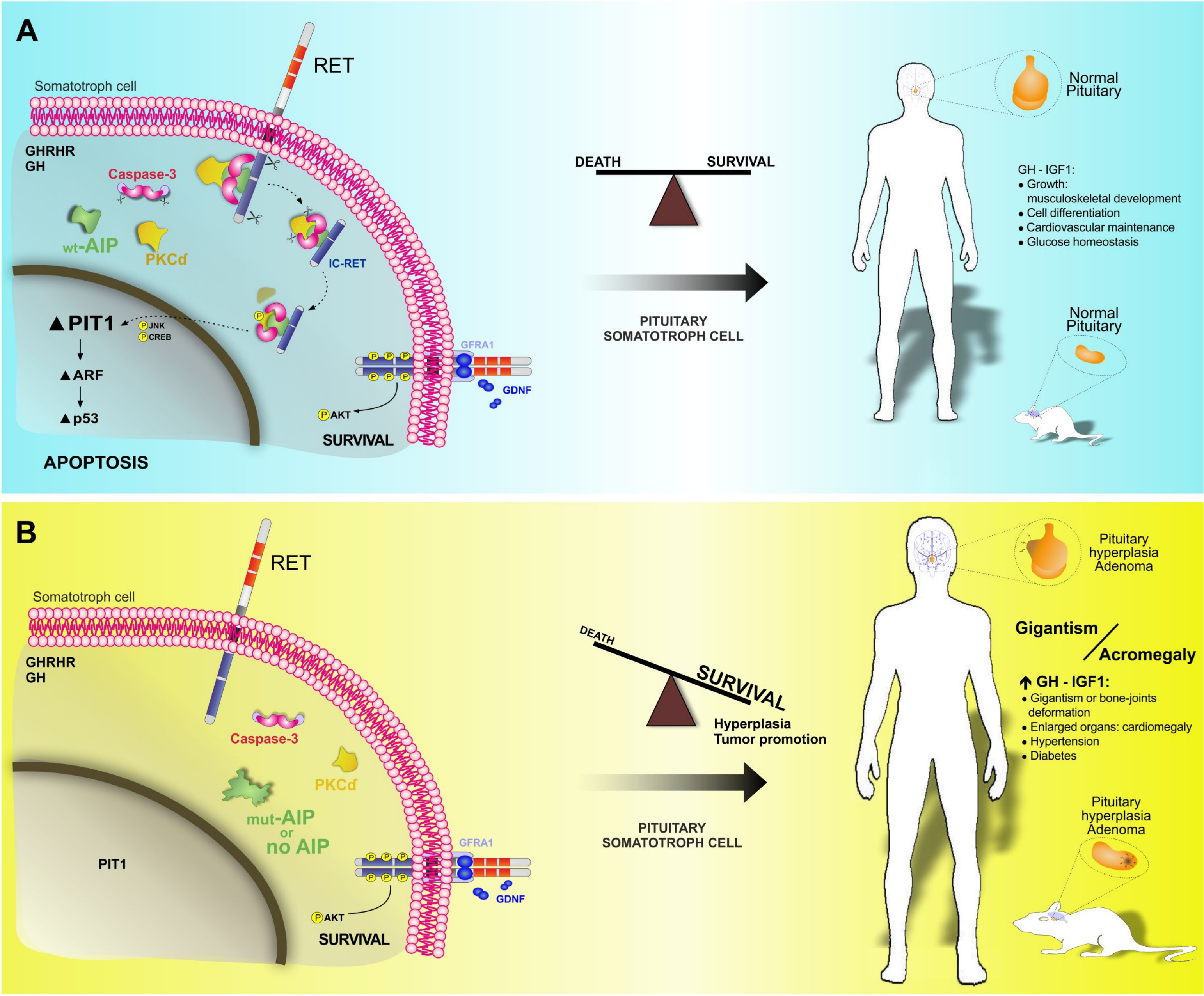

A
RET
Somatotroph cell
GHRHR
GH
Caspase-3
wt-AIP
PKCď
IC-RET
PIT1
JNK
CREB
ARF
p53
GFRA1
GDNF
AKT
SURVIVAL
APOPTOSIS
DEATH
SURVIVAL
PITUITARY
SOMATOTROPH CELL
Normal Pituitary
GH - IGF1:
• Growth: musculoskeletal development
• Cell differentiation
• Cardiovascular maintenance
• Glucose homeostasis
Normal Pituitary
B
RET
Somatotroph cell
GHRHR
GH
Caspase-3
PKCď
mut-AIP
or
no AIP
PIT1
GFRA1
GDNF
AKT
SURVIVAL
DEATH
SURVIVAL
Hyperplasia
Tumor promotion
PITUITARY
SOMATOTROPH CELL
Pituitary hyperplasia Adenoma
Gigantism / Acromegaly
↑ GH - IGF1:
• Gigantism or bone-joints deformation
• Enlarged organs: cardiomegaly
• Hypertension
• Diabetes
Pituitary hyperplasia Adenoma

Supplementary Information:
RET signalling provides tumorigenic mechanism and tissue specificity for AIP-related gigantism
Oncogene
Garcia-Rendueles et al

- Extended text related to *Functional classification of AIP mutations into two groups: protein present or absent* **page 3**
- Supplementary Tables 1 and 2 **page 5 - 6**
- Supplementary Figure 1 – 2 - 3 – 4 **page 7 – 13**
- Extended text related with *3D model and RET, Caspase-3, PKCd and AIP molecular interaction based on PLA results*, and Supplementary Figure 5 **page 14 - 15**
- Supplementary Figure 6 **page 16 - 18**
- Extended text related to *Phenotype in female rats injected at birth with wtAIP or AIP variants*, and Suppl Figure 7 **page 19 - 22**
- Extended text related to In silico study of RNA expression retrieved from ReGEO GSE63357, and Supplementary Figure 8 **page 23 - 24**
- Graphical Abstract **page 25**
- Supplementary. Methods: extended version **page 26**

Supplementary Methods Tables 1-2-3-4-5-6 **page 39**

References **page 53**

Extended Text related to:

Functional classification of *AIP* mutations into two groups: protein present or absent (Suppl Table 1 and Suppl Table 2, and Suppl. Fig 1)

We selected nine missense *AIP* variants identified in familial or simplex pituitary adenoma patients (Suppl. Table 1A) and one truncation hotspot variant. Most of these variants (R16H, V49M, C238Y, I257V, R271W, R304Q, R304*, R325Q) were identified in patients with young-onset acromegaly or prolactinoma, similar to patients with the truncation hotspot R304* (Beckers et al, Endocr. Rev. 2013; 34: 239; Hernandez-Ramirez LC, et al. J. Clin. Endocrinol. Metab. 2015; 100: E1242). Protein half-life of these variants was previously measured *in vitro* with cycloheximide chase experiments in HEK293 cells (Hernandez-Ramirez LC, et al. J. Clin. Endocrinol. Metab. 2016; 101: 3144; Yang JZ et al, Endocrine Abst. 2018; 59: P127) and their functional role has been studied in various functional assays, such as proliferation (Leontiou CA, et al. J. Clin. Endocrinol. Metab. 2008; 93: 2390), yeast two-hybrid assay for phosphodiesterase PDE4A5 interaction (Leontiou CA, et al. J. Clin. Endocrinol. Metab. 2008; 93: 2390; Igreja S, et al. Hum. Mutat. 2010; 31: 950; Bolger GB, et al. Endocr. Relat. Cancer 2016; 23: 419), a luciferase assay for cAMP-driven transcription (Formosa R, Vassallo J. Horm. Cancer. 2017; 8: 174), and rescuing the deleterious phenotype in drosophila larva *AIP*-orthologue knockout (Aflorei ED, et al. J. Med. Genet. 2018; 55: 522). But the pathogenicity of some of these variants is still questionable, since no assay has been fully validated for clinical use. On the other hand, two of these variants (R188Q, E245K) are associated with a slightly less typical phenotype of a macro or macroprolactinoma, lack of family history and previous experimental data not supporting pathogenicity (Suppl. Table 1). The I257V variant affects a key amino acid in the conserved tetratricopeptide structure of AIP.

In Supplementary Table 2, we performed *in silico* analysis of the 9 missense and R304* truncating variants using the most recent versions of i) multiple parameter assessing tools, combining clinical phenotype and genetic predictor algorithms (ClinVar, VarSome, CADD1.6, Ensembl Variant Effect Predictor VEP, REVEL, MetaIR), ii) protein-structure based algorithms (HoTMuSic, PoPMuSic, SNPMuSic, SIFT, FATHMM-XF, PolyPhen2, PROVEAN, MutationAssessor) and iii) assessed minor

allele frequencies (MAF) in the gnomADv3 (genomes) and v2.1 (exomes+genomes) and ExAC databases (now available inside gnomAD). For the integrative algorithms there is no consensus for many of the variants with conflicting interpretations for ClinVar, benignity for VarSome and deleteriousness for CADD v1.6. Some VUS alterations that are considered as neutral or benign in VarSome, are considered as deleterious or pathogenic in CADD1.6, PoPMusic or gnomAD. While there is consensus between the prediction programs for R271W and C238Y as pathogenic and E245K as benign, there are variable predictions for the other missense variants.

One of the ways AIP variants can be classified is based on their protein half-life (Fig. 1A right and Suppl. Table 1). We classified a group of missense variants with normal half-life, similar to wtAIP in this assay (R16H, V49M, R188Q, E245K, I257V, R304Q, R325Q), short half-life (C238Y and R271W) or very-short half-life (R304*) (Fig 1B), similar to our previous study in HEK293 cells (Hernandez-Ramirez LC, et al. J. Clin. Endocrinol. Metab. 2016; 101: 3144; Yang JZ et al, Endocrine Abst. 2018; 59: P127) except for V49M and I257V that in pituitary cells have normal half-life too. We transfected each individual *AIP* variant in the absence or presence of human RET (short isoform, RET_S) and this did not affect variant half-life (Fig 1C). To represent AIP deficiency, we transfected rat *Aip* (*rAip*) siRNA or scramble siRNA (Sc siRNA) as control. Endogenous AIP protein was successfully repressed in the absence or presence of RET (Fig 1D).

Our chosen model was the rat pituitary GH4C1 cell line, derived originally from a pituitary tumour of a female Wistar/Fu rat following cranial irradiation. GH4C1 cells present a clear somatotroph phenotype characterized by high RNA levels of the transcription factor *Pit1* and *Gh*, while expression of the two other PIT1-dependent hormones were low (*Prl*), or very low (*Tshb*) (Suppl. Fig 1B). The other hormones such as corticotropin (*Pomc*) and gonadotropins (*Lhb* and *Fshb*) also had a low expression together with non-somatotroph transcription factors *Sf1* or *Prop1*. GH4C1 cells expressed *Aip, Gdnf* and *Gfra1* but did not express *Ret*. We have previously been unable to generate a RET-expressing population of GH4C1, as stable high RET expression prevented cell growth in these cells (Canibano C, et al. EMBO J. 2007; 26: 2015).

Supplementary Table 1: ***AIP*** **variants used in this study: clinical data, genome data and review of the literature.**
N/A: Not apply / Not studied/ Not performed. References indicated as PMID.

Position	Variant name		Variant type	First patient described: Youngest known age								Number of Homozygotes (GnomAD v2.1.1)	Number of Homozygotes (GnomAD v3)	Pituitary tumour types of all described patients	Functional studies performed so far: Protein half-life in HEK293 cell: comparison with our data in pituitary cells (underlined if coincident with Fig. 1)			Coincidence with *AIP* -FIPA patient	Consensus clinic expert opinion so far (with some dissention in the literature)	References (PMID)		
	DNA level	Protein level		Onset	Diagnosis	Adenoma size (Macro)	Tumor type	Giant	LOH	Reference (PMID)	More than 1 patient affected in the family				PMID: 27253664 / #	Inability to rescue CG1847 knockout Fruit fly lethality (suggesting pathogenic)	Loss of binding to PDE4A5 (% of wild-type)			Cases	Functional studies	Other
67483205	c.47G> A	p.R16H	Missense	N/A	15	Yes	GH	N/A	No	17242703	Yes	2 (exomes)	0	GH, PRL, NFPA	<u>Normal</u>	No	Reduced (<30%)	Yes	Likely benign	17242703; 17244780; 17609395; 18484068; Yaneva, M et al. (2008)**; 19794292; 21753072; 22319033; Baciu et al., (2012)***; 22915287; Baciu et al, (2013)****; 25614825; 29074612	19366855; 20506337; 27267386; 27253664; 29632148	21348957; 22720333; 22915287
67487051	c.145G> A	p.V49M	Missense	N/A	28	N/A	GH	Yes	No	17371465	No	0	0	GH	Short (Normal in pituitary cells)	N/A	No, similar (>30%)	Yes	Likely pathogenic	17371465	20506337; 27267386; 27253664	21348957
67490132	c.563G>A	p.R188Q	Missense	24	24	No	PRL	N/A	N/A	22319033	No	N/A	0	PRL	<u>Normal</u>	N/A	N/A	No	Likely benign	22319033	---	23371967
67490383	c.713G > A	p.C238Y	Missense	18	19	Yes	GH	No	Yes	9920092	Yes	0	N/A	GH	<u>Very short</u>	Yes	Severe reduction (<20%)	Yes	Pathogenic	9920092; 26186299; 29440248	20506337; 18381572; 27267386; 27253664; 29632148	21348957; 22720333
67490403	c.733G>A	p.E245K	Missense	N/A	24	Yes	PRL	No	N/A	24050928	No	N/A	0	PRL	<u>Normal</u>	N/A	N/A	No	Likely benign	24050928	---	---
67490439	c.769A> G	p.I257V	Missense	N/A	39	Yes	TSH	N/A	N/A	Montanana, CF et al. (2009)*	No	N/A	N/A	TSH	Short (Normal in pituitary cells)	N/A	Reduced (<30%)	No	Pathogenic	Montanana, CF et al. (2009)* 20685857	20506337; 27267386; 27253664	21348957; 22720333
67490811	c. 811C> T	p.R271W	Missense	15	16	Yes	GH	Yes	N/A	17244780	No	0	N/A	GH, GH&PRL, PRL	<u>Very short</u>	N/A	Severe reduction (<20%)	Yes	Pathogenic	17244780; 19684062; 20506337; 21753072; 26186299; 29440248	18381572; 19366855; 20506337; 27267386; 27253664	21348957; 22720333
67490911	c.911G> A	p.R304Q	Missense	17	18	Yes	GH	Yes	Yes	17242703	Yes	2 (exomes)	0	GH, PRL, GH&PRL, ACTH	<u>Normal</u>	No	Reduced (<30%)	Yes	Likely benign	17242703; 17609395; 18381572; 19366855; 20530095; 20506337; 21753072; 22319033; 23321498; 23633209; 25614825; 26186299; 29074612	18381572; 19366855; 20506337; 27267386; 272536646; 29507682; 29632148	21348957; 22720333
67490974	c.974G> A	p.R325Q	Missense	16	18	Yes	PRL	N/A	Yes	22319033	Yes	0	0	PRL	<u>Normal</u>	No	N/A	Yes	Likely benign	22319033; 24078436	27253664; 29632148	---

(*) Montanana, CF et al. TSH-secreting pituitary adenoma in a male patient with a novel missense AIP mutation. *91st ENDO,* P1-668 (2009)

(**) Yaneva, M et al. Aryl hydrocarbon receptor interacting protein gene mutations in bulgarian FIPA and young sporadic pituitary adenoma patients. *91st ENDO,* P3-520 (2008)

(***) Baciu I et al. Screening of AIP mutations in young Romanian patients with sporadic pituitary adenomas Presented at Society for Endocrinology BES, Italy. *Endocrine Abstracts* **29,** P786 (2012)

(****) Baciu I et al. The p.R16H (C.47G>A) AIP gene variant in a case with invasive non-functioning pituitary macroadenoma and Screening of a Control Cohort. *Acta Endo* Buc **9** (1), 97-108 (2013)

Supplementary Table 2: ***In silico*** **study of human** ***AIP*** **variants.** Variants are listed following the order of the amino acid sequence. The last available version of each algorithm was used (column in the left under Update). We have made three groups: those algorithms integrating multiple clinical and genetic tools, those related to protein structure and functional domains, and a third group related to allele frequency in next generation sequencing studies in normal population. Thresholds for pathogenicity are indicated in each algorithm. Some algorithms have an alternative threshold proposed by Li et al (see reference at the bottom). This threshold is shown at the most left column; underlined values within variants indicate pathogenic values over this threshold. Red background: when values resulted over the threshold (Pathogenic/ Likely Pathogenic/ Likely Deleterious/ Likely disease causing/ Damaging/ Destabilizing/ Deleterious/ Probably damaging/ Medium); Carnation background: when values were near the threshold (Likely pathogenic/ Likely deleterious); Green background: when values resulted within the threshold for normality (Benign/ Likely benign/ Tolerated/ Stabilizing/ Neutral/ Low); White background (VUS/ Conflicting interpretations/ not included).

Alternative threshold (A)	Update		AIP Variants (NM_0038977.3)									
			c.47G>A (p.R16H)	c.145G>A (p. V49M)	c.563G>A (p.R188Q)	c.713G>A (p.C238Y)	c.733G>A (p.E245K)	c.769A>G (p.I257V)	c. 811C>T (p.R271W)	c.911G>A (p.R304Q)	c.910C>T (p.R304*)	c.974G>A (p.R325Q)
		**Reference**	rs145047094	rs1063385	rs866556486	rs267606569	rs150645662	rs267606575	rs267606579	rs104894190	rs104894195	rs754619109
		**Position**	67483205	67487051	67490132	67490383	67490403	67490439	67490811	67490911	67490910	67490974
		**Variant type**	Missense	Missense	Missense	Missense	Missense	Missense	Missense	Missense	Nonsense	Missense
		**Protein localization**	N-terminal (PPIase-like domain)	N-terminal (PPIase-like domain)	C-terminal (TPR1 motif)	C-terminal (TPR2 motif)	C-terminal (TPR2 motif)	C-terminal (TPR2 motif)	C-terminal (TPR3 motif)	C-terminal (alpha-helix)	C-terminal (alpha-helix)	C-terminal (alpha-helix)
		**Secondary Structure (B)**	C	E	H	H	H	H	H	H	H	H
		**Tools integrating multiple algorithms for comparing variants**										
----	2020	**ClinVar / dbSNP** Revision date indicated in each variant	Conflicting interpretations: Likely benign (2017; 2019 (2)) VUS (2012)	Conflicting interpretations: Likely benign (2017; 2019) VUS (2012; 2019(2))	VUS (2019; 2020)	Likely pathogenic (2012)	Conflicting interpretations: Likely benign (2017) VUS (2019)	Likely pathogenic (2012)	Pathogenic (2012)	Conflicting interpretations: Benign (2019) Likely benign (2017) Pathogenic (2017;2018) VUS (2016; 2018; 2019(2))	Pathogenic (2011; 2012; 2019)	VUS (2019)
----	2020	**VarSome** It combines rules established by the ACGM classifier with several databases, clinical evidence, allelic frequency and conservation	Benign	Benign	Likely benign	Likely pathogenic	Benign	Likely benign	Likely pathogenic	Benign	Pathogenic	Benign
----	2020	**CADD v1.6** Integrating conservation and functional information into one deleteriousness score. CADD score ranges from 1 to 99 (Higher score>Greater deleteriousness) PHRED; soft threshold >15; hard threshold >20	24.3 Likely deleterious	21.5 Likely deleterious	15.41 Likely benign	26.9 Likely deleterious	3.943 Likely benign	19.68 Likely deleterious	24.7 Likely deleterious	23.5 Likely deleterious	38 Likely deleterious	22.2 Likely deleterious
----	2018	**Ensembl Variant Effect Predictor VEP** Clinical significance; gnomAD	Likely benign/ VUS	Likely benign/ VUS	----	Likely pathogenic	----	Likely pathogenic	Pathogenic	Likely benign/ Pathogenic/ VUS	Pathogenic	VUS
≥0.4335	2015	**REVEL** Integrates scores from MutPred, FATHMM v2.3, VEST 3.0, PolyPhen-2, SIFT, PROVEAN, MutationAssessor, MutationTaster, LRT, GERP++, SiPhy, phyloP, and phastCons. Threshold ≥0.4	0.777 Likely disease causing	0.708 Likely disease causing	0.332 Likely benign	0.815 Likely disease causing	0.179 Likely benign	0.353 Likely benign	0.785 Likely disease causing	0.31 Likely benign	----	0.376 Likely benign
> 0.2645	2011	**MetaLR** Use logistic regression to integrate nine independent variant deleteriousness scores and allele frequency information. Threshold >0.5	0.661 Damaging	0.819 Damaging	0.14 Tolerated	0.545 Damaging	0.16 Tolerated	0.128 Tolerated	0.623 Damaging	0.22 Tolerated	----	0.415 Tolerated
		**Algorithms based in protein structure and function**										
----	2018; PDB 4AIF/ 2LKN 2012/ 2013	**PoPMuSiC** Effect on thermodynamic stability. Threshold ΔΔG>0 kcal/mol	0.02 Destabilizing	0.69 Destabilizing	0.22 Destabilizing	0.64 Destabilizing	-0.38 Stabilizing	1.23 Destabilizing	0.20 Destabilizing	-0.02 Stabilizing	----	----
		**HoTMuSiC** Effect on thermal stability. Threshold ΔTm<0K	-0.53 Destabilizing	-1.85 Destabilizing	-1.21 Destabilizing	-3.1 Destabilizing	0.31 Stabilizing	-3.87 Destabilizing	-1.32 Destabilizing	-0.89 Destabilizing	----	----
		**SNPMuSiC** Prediction of the effect on protein stability. Threshold >0	-0.44 Neutral	-0.37 Neutral	-0,54 Neutral	0.82 Deleterious	-0,64 Neutral	-0.15 Neutral	0.57 Deleterious	-0.37 Neutral	----	----
≤ 0.010	2018	**SIFT** Based on sequence homology and physicochemical similarity with the alternate amino acid. Threshold ≤0.05	0.025 Damaging	0.002 Damaging	0.31 Tolerated	0.001 Damaging	0.67 Tolerated	0.67 Tolerated	0 Damaging	0.01 Damaging	----	----
----	2018	**FATHMM-XF** Combination of sequence homology and conserved protein domains with hidden Markov models tolerance of the protein/domain to mutations. Threshold=0.5	0.368 Benign	0.304 Benign	0.249 Benign	0.923 Pathogenic	0.414 Benign	0.796 Pathogenic	0.9 Pathogenic	0.166 Benign	----	0.290 Benign
≥0.9305	2016	**PolyPhen2-Hum Div.** Based on sequence homology, phylogenetic and structural information. In particular, was compiled from all damaging alleles with known effects on the molecular function causing human Mendelian diseases, together with differences between human proteins and their closely related homologs. Threshold ≥0.453	0.969 Probably damaging	0.975 Probably damaging	0.003 Benign	0.994 Probably damaging	0.015 Benign	0.01 Benign	1.0 Probably damaging	0.047 Benign	----	0.01 Benign
≥0.662	2016	**PolyPhen2-Hum Var.** Based on the sequence homology, phylogenetic and structural information. In particular, consisted of all human disease-causing mutations together with common human nsSNPs MAF>1% which were treated as non-damaging. Threshold ≥0.447	0.416 Benign	0.763 Probably damaging	0 Benign	0.885 Probably damaging	0.007 Benign	0.008 Benign	1.0 Probably damaging	0.018 Benign	----	0.002 Benign
≤ -2.255	2015	**PROVEAN** Based on the amino acid residue at the position of interest and the quality of sequence alignment derived from the neighbourhood flanking sequences. Threshold ≤-2.5	-2.54 Deleterious	-2.30 Neutral	-1.245 Neutral	-7.45 Deleterious	- 0.973 Neutral	-0.39 Neutral	-7.38 Deleterious	-1.85 Neutral	-11.20 Deleterious	-0.67 Neutral
>2.2625	2014	**MutationAssessor** Based on evolutionary conservation of the affected amino acid in protein homologs. Threshold >1.9	1.78 Low	3.29 Medium	0.155 Neutral	2.945 Medium	1.27 Low	0.48 Neutral	3.57 High	1.725 Low	----	0.49 Neutral
		**Algorithms based on genomic MAF in the normal population**										
----	2019	**gnomAD v3** Genomes; GRCh38. Threshold <0.1%-0.01% (recessive or dominant allele)	0.001932	0.00005582	0.00002791	----	0.0001186	----	---	0.001130	0.00001397	0.0001116
	2018	**gnomAD v2.1.1** Genomes&exomes; GRCh37. Threshold <0.1%-0.01% (recessive or dominant allele)	0.002082	0.00005582	----	0.000004025	0.00004652	----	0.000004017	0.001568	0.00001436	0.00006138
----	2016	**Exome Aggregation Consortium** ExAC. Threshold <0.1%-0.01% (recessive or dominant allele)	0.001956	0.0002395	----	0.00000842	0.00005059	----	----	0.001458	0.00001715	0.0000585
			c.47G> A (p.R16H)	c.145G> A (p. V49M)	c.563G>A (p.R188Q)	c.713G> A (p.C238Y)	c.733G>A (p.E245K)	c.769A> G (p.I257V)	c. 811C> T (p.R271W)	c.911G> A (p.R304Q)	c.910C> T (p.R304*)	c.974G> A (p.R325Q)

(A) Modified by Li J.et al. "Performance evaluation of pathogenicity-computation methods for missense variants". *Nucleic Acids Res*, **46**(15), 7793-7804 (2018)
(B) Secondary structure: C (coiled), E (beta-strand) and H (alpha-helix).

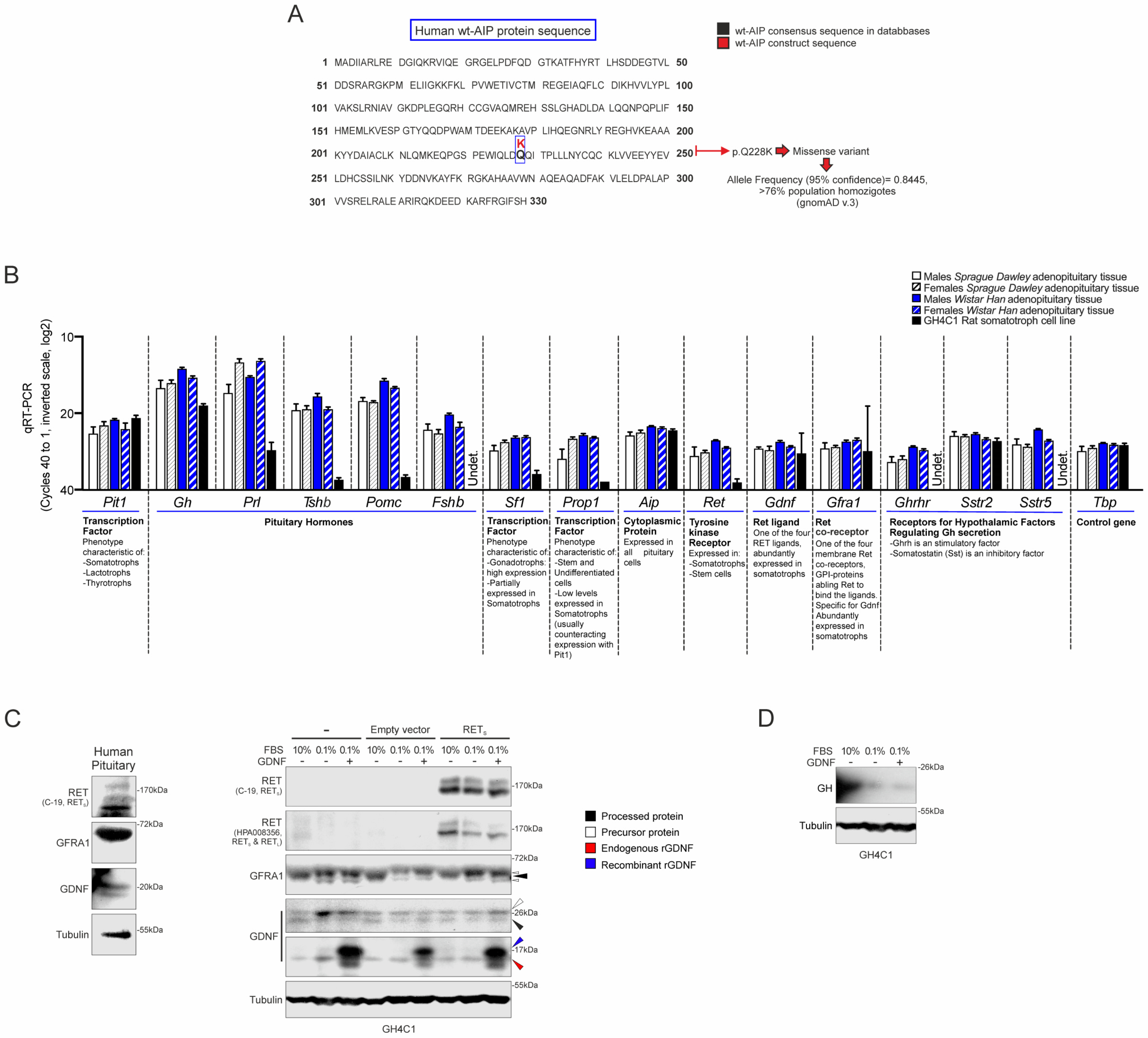

A
Human wt-AIP protein sequence
wt-AIP consensus sequence in databbases
wt-AIP construct sequence
1 MADIIARLRE DGIQKRVIQE GRGELPDFQD GTKATFHYRT LHSDDEGTVL 50
51 DDSRARGKPM ELIIGKKFKL PVWETIVCTM REGEIAQFLC DIKHVVLYPL 100
101 VAKSLRNIAV GKDPLEGQRH CCGVAQMREH SSLGHADLDA LQQNPQPLIF 150
151 HMEMLKVESP GTYQQDPWAM TDEEKAKAVP LIHQEGNRLY REGHVKEAAA 200
K
201 KYYDAIACLK NLQMKEQPGS PEWIQLDQQI TPLLLNYCQC KLVVEEYYEV 250
251 LDHCSSILNK YDDNVKAYFK RGKAHAAVWN AQEAQADFAK VLELDPALAP 300
301 VVSRELRALE ARIRQKDEED KARFRGIFSH 330
p.Q228K
Missense variant
Allele Frequency (95% confidence)= 0.8445,
>76% population homozigotes
(gnomAD v.3)
B
Males Sprague Dawley adenopituitary tissue
Females Sprague Dawley adenopituitary tissue
Males Wistar Han adenopituitary tissue
Females Wistar Han adenopituitary tissue
GH4C1 Rat somatotroph cell line
qRT-PCR
(Cycles 40 to 1, inverted scale, log2)
10
20
40
Undet.
Pit1
Gh
Prl
Tshb
Pomc
Fshb
Sf1
Prop1
Aip
Ret
Gdnf
Gfra1
Ghrhr
Sstr2
Sstr5
Tbp
Transcription Factor
Phenotype characteristic of:
-Somatotrophs
-Lactotrophs
-Thyrotrophs
Pituitary Hormones
Transcription Factor
Phenotype characteristic of:
-Gonadotrophs: high expression
-Partially expressed in Somatotrophs
Transcription Factor
Phenotype characteristic of:
-Stem and Undifferentiated cells
-Low levels expressed in Somatotrophs (usually counteracting expression with Pit1)
Cytoplasmic Protein
Expressed in all pituitary cells
Tyrosine kinase Receptor
Expressed in:
-Somatotrophs
-Stem cells
Ret ligand
One of the four RET ligands, abundantly expressed in somatotrophs
Ret co-receptor
One of the four membrane Ret co-receptors, GPI-proteins abling Ret to bind the ligands. Specific for Gdnf. Abundantly expressed in somatotrophs
Receptors for Hypothalamic Factors Regulating Gh secretion
-Ghrh is an stimulatory factor
-Somatostatin (Sst) is an inhibitory factor
Control gene
C
Human Pituitary
RET (C-19, RETS)
GFRA1
GDNF
Tubulin
-170kDa
-72kDa
-20kDa
-55kDa
-
Empty vector
RETS
FBS 10% 0.1% 0.1% 10% 0.1% 0.1% 10% 0.1% 0.1%
GDNF - - + - - + - - +
RET (C-19, RETS)
RET (HPA008356, RETS & RETL)
GFRA1
GDNF
Tubulin
-170kDa
-170kDa
-72kDa
-26kDa
-17kDa
-55kDa
GH4C1
Processed protein
Precursor protein
Endogenous rGDNF
Recombinant rGDNF
D
FBS 10% 0.1% 0.1%
GDNF - - +
GH
Tubulin
-26kDa
-55kDa
GH4C1

Supplementary Figure 1: Models used to explore AIP – RET molecular relationship in the pituitary.

A) Sequence of human wtAIP protein used for introduction of the variants studied. The protein sequence contains one amino acid change (Q228K) with respect to the accepted in databases (Ensembl, Uniprot). AIP K228 is present with a population allele frequency of 0.8445 in normal population genomes (gnomADv3), and more than 75% of the sequenced genomes are homozygous for this variant. We considered that wtAIP-K228 is a more realistic situation as wt allele than AIP-Q228.

B) The rat pituitary cell line GH4C1 presents a somatotroph phenotype with AIP but very low RET receptor mRNA and protein expression**.**

Quantitative mRNA expression detected by TaqMan RT-PCR assays. A log2 scale shows directly the PCR cycles used. GH4C1 (black bars) are compared with whole adenohypophysis (or adenopituitary, AP) tissue from young adult (90 days old) male and female rats of two strains, Sprague Dawley and Wistar Han. GH4C1 were originally derived from Wistar rats. However, Sprague Dawley is the strain currently used as an animal model for pituitary research. No major differences are seen between the two.

In general, it is considered that a whole anterior pituitary will have a major endocrine epithelial cell population and a smaller non-secretory mesenchymal population together with blood vessels. The epithelial endocrine secretory population is unequally distributed: somatotrophs (*Gh*): >45% in males, 40% in females; lactotrophs (*Prl*): <30% in males; 35% in females; gonadotrophs (*Fsh* and *Lh*),11%; corticotrophs (*Pomc*) 9%; thyrotrophs (*Tshb*) 5%. Phenotype of secretory cells is defined by hormone production and high expression of specific transcription factors and receptors for hypothalamic regulatory hormones (*Ghrhr*, *Sstr2*, *Sstr5*).

GH4C1 express high levels of *Pit1* and *Gh* and reduced levels of *Prl*. Minimal amount of other hormones and non-somatotroph transcription factors are detected. GH4C1 has *Aip, Gdnf* and *Gfra1* but minimal *Ret* expression. GH4C1 does not express *Ghrhr* but express *Sstr2* and *Sstr5* receptors.

(n=4 animals per group. Media ±SEM of cycles from the 4 animals in each group).

C) The RET / GDNF / GFRA1 expression at protein level in GH4C1 confirms the RNA expression data. Three datasets are compared: mock transfected cells, cells transfected with empty plasmid vector, and cells transfected with the RET_S receptor. All cells were seeded in the same day and maintained in parallel for 24 hours. After this, cells were washed with PBS three times, and replaced by fresh full medium (10% FBS), deprived medium (0.1% FBS) or deprived medium + 50 ng/mL GDNF. Cells were lysed following 1 hour incubation. Western blots are shown for the RET pathway proteins (right) in comparison with human total pituitary extracts (left).

The RET receptor was detected with two antibodies. C-19 is exclusively for RET_S detection: only a very faint band is detected after 24 hours exposure in untransfected extracts, while strong expression is seen in RET_S transfected cells. HPA008356 recognizes an epitope common to RET_L and RET_S isoforms: faint bands are detected in untransfected cells, while strong expression is detected in RET_S transfected cells.

GFRA1 protein is well expressed in GH4C1 cells. During its sorting to the plasma membrane through the ER and Golgi, GFRA1 is glycosylated in three residues and has 14 intrachain disulfide bonds; finally, GFRA1 must be processed by deleting its 36 aa C-term tail when bound to phosphatidylinositol at the luminal aspect of the ER membrane. This mature processed protein is detected in full medium (10% FBS; black arrow). After deprivation, a doublet of precursor proteins is detected (white arrows).

GDNF protein, a secreted fator, is expressed in GH4C1. After translation and entering the ER, the 211 aa full length GDNF protein loose the 19 aa signal peptide, and the remaining 192 aa with two glycosylation sites and two disulfide bonds is processed to a shorter mature glycosylated form of 134 aa. As seen in the western around the 26 kDa molecular weight marker, after deprivation GDNF is less processed; compare precursor protein (white arrow) with processed protein (black arrow). At the 17 kDa molecular weight marker, the final GDNF (red arrow, either to be secreted, or uptaken from outside through endocytosis) is seen. A strong band of recombinant rGDNF (blue arrow) is seen in the corresponding lanes.

D) GH4C1 express abundant GH protein. Western blot showing 20 kD GH expression in growing conditions (full medium, 10% FBS) or one hour after serum deprivation (0.1% FBS) alone or with 50 ng/mL GDNF. This abundant expression is expected from the *Gh* mRNA expression shown in B (black bar).

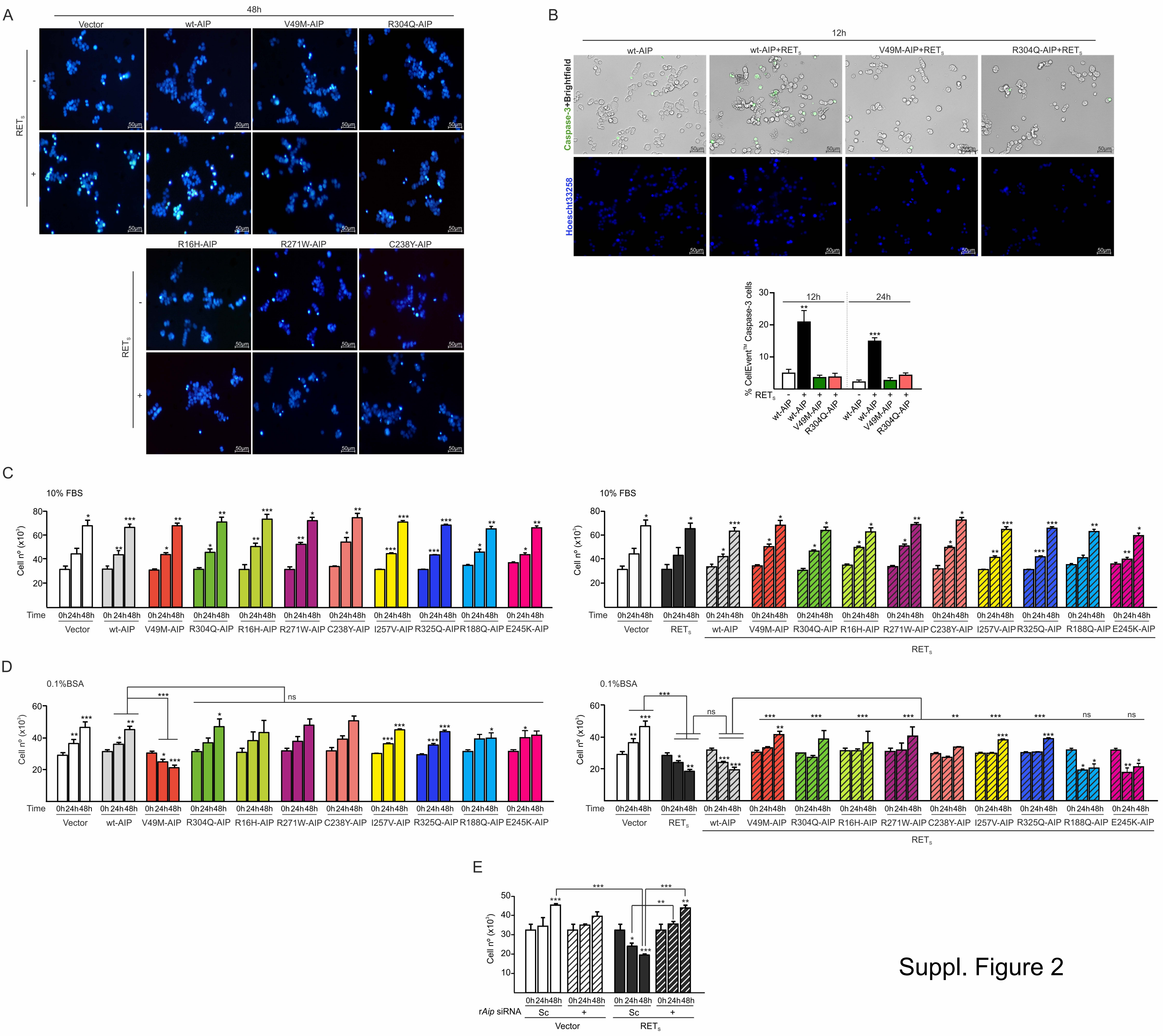

Suppl. Figure 2

Supplementary Figure 2: Variant *AIP* or endogenous *Aip* repression blocks the physiological RET/PIT1/*CDKN2A*-ARF/p53 apoptosis pathway: effects on apoptosis and cell growth.

A) Representative microphotographs for Hoechst 33258 staining 48 hours after deprivation of GDNF. **B)** CellEvent fluorescent cellular assay at 12h confirmed that RET + wtAIP caspase-dependent apoptosis was prevented by AIP variants V49M and R304Q. **C)** In the presence of full medium (10% FBS) neither wtAIP nor AIP variants affected cell growth in the absence (left) or the presence (right) of the RET_S receptor. **D) Left:** When deprived of GDNF (0.5% FBS), transfection of wt*AIP* or *AIP* variants do not affect cell growth, although in low serum cells grow more slowly. The V49M AIP variant is the only one having a deleterious effect inducing some cell death. This germline variant was found in heterozygous form (no loss-of-heterozygosity) in a patient's tumour sample. **Right:** Cells transfected with RET_S and later changed to low serum die in the presence of wtAIP. This is prevented by *AIP* variants that prevent apoptosis (V49M, R304Q, R16H, R271W, C238Y, I257V, R325Q) allowing cells to recover cell growth. R188Q and E245K, the two AIP variants that do not alter RET-induced apoptosis, reduce cell growth. **E)** r*Aip* or Scramble (Sc) siRNA do not affect cell growth in low serum. However, when transfected with RET_S cell death is induced and cells do not grow. Cell growth is recovered in RET_S-transfected cells with r*Aip* siRNA but not with scrambled (Sc) control siRNA.
(A-E: 4 independent experiments with 4 replicates in each. Two-ways ANOVA with Sidak's multiple comparison test correction, B; Two-ways with repeated measures ANOVA with Sidak's multiple comparison test correction C-D; Three-ways with repeated measures ANOVA with Tukey's multiple comparison test correction, E. *, $p<0.05$; **, $p<0.01$; ***, $p<0.001$; ns, non-significant)

Suppl. Figure 3

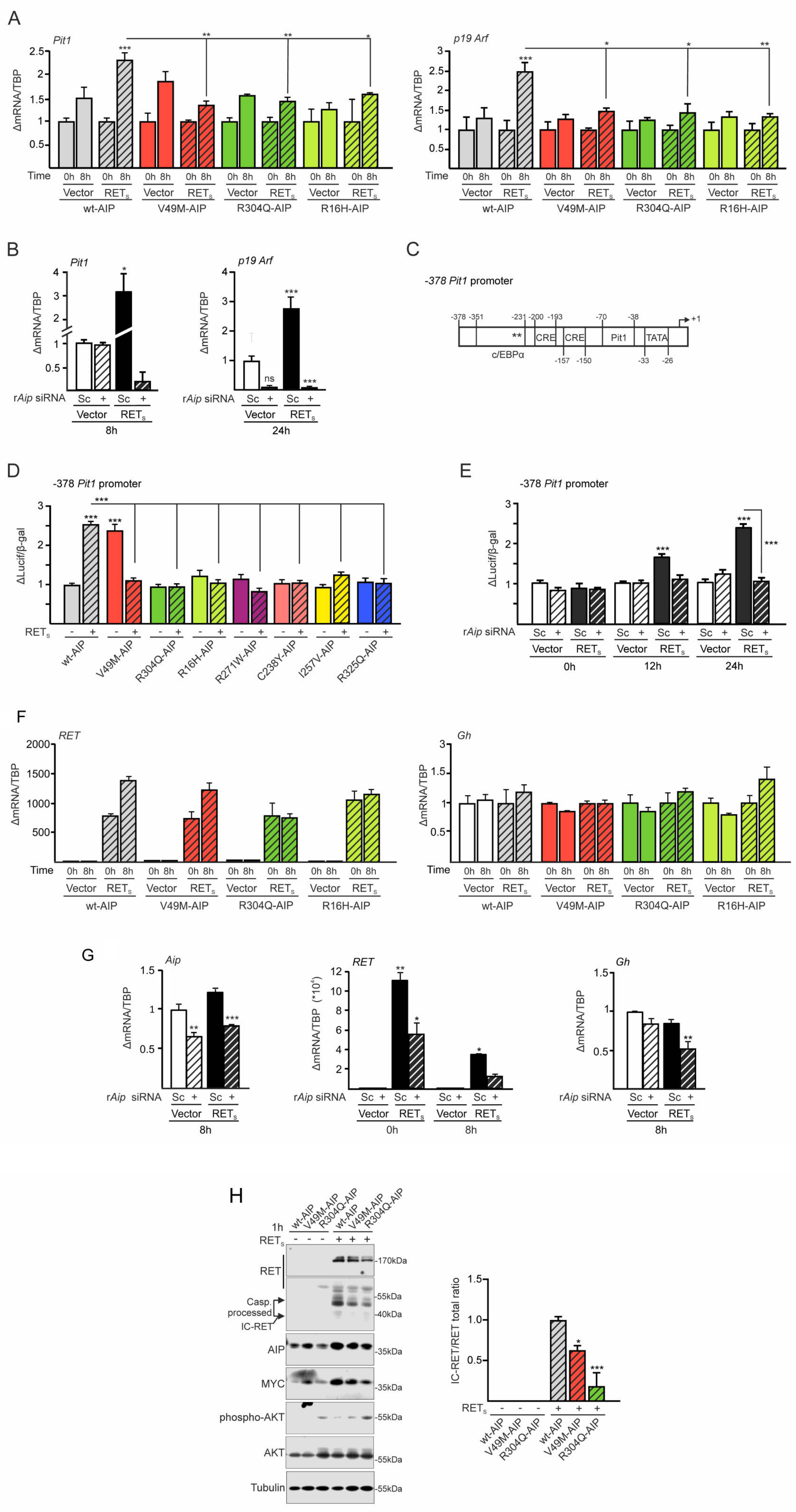

Supplementary Figure 3: Pathogenic *AIP* variants or *Aip* repression blocks Pit1 gene expression and promoter activation indicating an earlier effect on the pathway: prevention of early RET processing. *AIP* transfection do not directly affect *RET* or *Gh* expression. Endogenous *Aip* repression reduces *Gh* expression.

A) *AIP* variants repress RET-induced increase in *Pit1* mRNA expression after GDNF deprivation. Consequently, PIT1-induced *Arf* mRNA increase is also reduced. **B)** r*Aip* siRNA blocks RET-induced increase in *Pit1* mRNA expression. Consequently, PIT1-induced enhanced *Arf* expression is also reduced. **C)** Cartoon showing the main sites in the *Pit1* promoter conserved in rat, mouse and human genes. Both CRE sites and the c/EBPa binding site are essential for RET-dependent induction. **D)** *AIP* variants block RET-induced activation of the *Pit1* promoter. **E)** r*Aip* siRNA block RET-induced activation of the *Pit1* promoter. **F)** *AIP* variants do not affect exogenous *RET* or endogenous *Gh* mRNA. **G)** r*Aip* siRNA only slightly reduces exogenous *RET* but acutely decrease endogenous *Gh* mRNA expression as corresponding with reduced *Pit1* mRNA expression. **H)** A short-time experiment after 1-hour deprivation shows reduced RET processing by caspase-3 in the presence of *AIP* variants. Quantification of n=3 experiments is shown in the right. Variant *AIP* moderately increase p-AKT levels indicating survival.

(4 independent experiments with 4 replicates in each except H, as indicated. Three-ways with repeated measures ANOVA with Tukey's multiple comparison test correction, A-D-E-F-G in exogenous RET; Two-ways ANOVA with Sidak's multiple comparison test correction, B-G in siRNA-only experiments and H). *, $p<0.05$; **, $p<0.01$; ***, $p<0.001$)

Suppl Figure 4

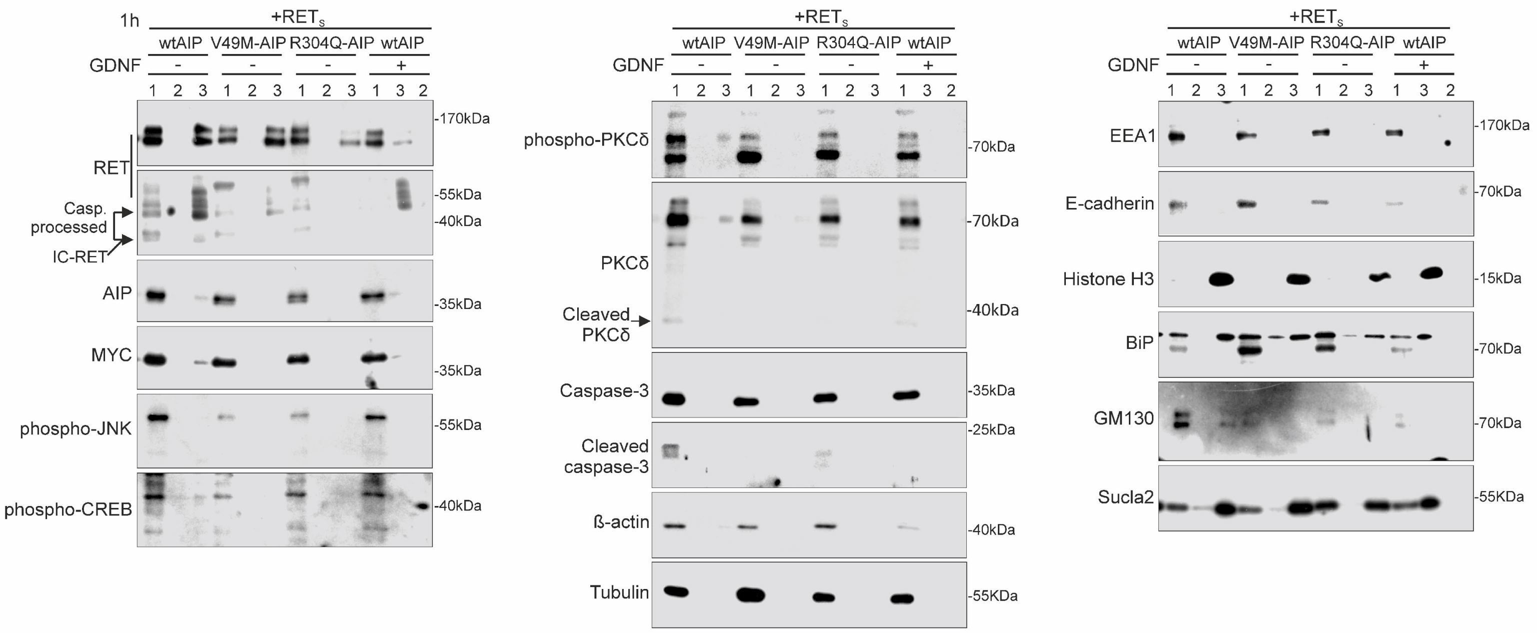

Supplementary Figure 4: Subcellular fractioning extracts: migration of the processed p-PKCδ/AIP complex to the nucleus. Standardization of subcellular fractioning in GH4C1 cells transfected with RET and wt or a pathogenic AIP variant and deprived for one hour in the absence (-) or presence (+) of GDNF. Three fractions were obtained per condition: plasma membrane/cytoplasm (1), wash out (2) and nuclear/perinuclear endoplasmic reticulum (ER) (3).
A detectable band of IC-RET, p-CREB, phospho-PKCδ / cleaved PKCδ and cleaved caspase-3 is seen in fraction 1 when wt*AIP* is transfected but markedly reduced or absent when V49M or R304Q *AIP* is transfected, or wt*AIP* condition is treated with ligand GDNF inducing RET-dimerization at the membrane. Total levels of AIP, MYC, caspase-3 and PKCδ are shown indicating equal transfection and loading.
In fraction 3 a minor band of AIP, MYC, phospho-PKCδ , PKCδ is observed in wt*AIP* but not in the *AIP* variants or in wtAIP treated with GDNF.
Protein controls for subcellular fractions: membrane/cytoskeleton (β-actin, Tubulin, 1); submembrane (EEA1, early endosome antigen 1); plasma membrane (E-cadherin, 1); nucleus (Histone H3, 3); lumen ER (BiP, Binding immunoglobulin protein, GRP-78 or heat shock 70 kDa protein 5, HSPA5; 1 and 3); Golgi (GM130, Golgi matrix protein, 1); Sucla 2 (Succinate--CoA ligase [ADP-forming] subunit beta, mitochondrial protein, mainly in 3, weak in 1).

Fraction 1 was enriched for the plasma membrane (E-cadherin). The submembrane fraction- cytoskeleton (β-actin and α-tubulin) proteins, early endosome (EEA1), ER (BiP) and partial Golgi vesicles (GM130) and mitochondria (Sucla 2). Fraction 2 was a wash step. Fraction 3 was enriched in nuclear (Histone H3), perinuclear ER (heavy form of BiP) and mitochondria (Sucla 2) proteins.

Extended Text related to the three dimensional (3D) interaction model (Suppl. Figure 5)

AIP is proposed to present a conformation either as multimeric or homotrimer (Hollingshead BD et al, J Biol Chem 2004; 279: 45652) and homology to FK506bp4 (model 1qz2.1), a homodimer (Hollingshead BD et al, J Biol Chem 2004; 279: 45652) or a monomer (Morgan RM et al, 2012; 7: e53339). We explored an exploratory *in silico* model based on the known X-ray diffraction structures for the four implicated proteins (caspase-3, PKCδ, IC-RET and AIP) that fit with results of the PLA assay using the Swiss-model and Chimera softwares (Pettersen EF et al, J Comput Chem 2004, 25:1605; Goddard TD et al, Protein Sci 2018, 27:14). A simulation model with AIP being a multimer or a dimer did not obtain any conformation explaining results of the PLA assay (data not shown). A model based on a monomer explained our PLA assay results (Suppl. Fig 5). In this exploratory model, AIP binds RET through R325, caspase-3 through V49 and PKCδ through R16 (N-terminal, near caspase-3), I257 (at the PKCδ catalytic subunit), and R304 (C-terminal, near RET); caspase-3 binds with a high affinity to the 707 consensus site of RET and binds PKCδ through the so-called regulatory region (subunit) where the caspase-3 consensus site is located. Based on these data, lack of AIP or presence of our pathogenic AIP variants may disrupt the RET/caspase-3/PKCδ/AIP complex resulting in reduced JNK activation and reduced apoptosis.

Suppl. Figure 5

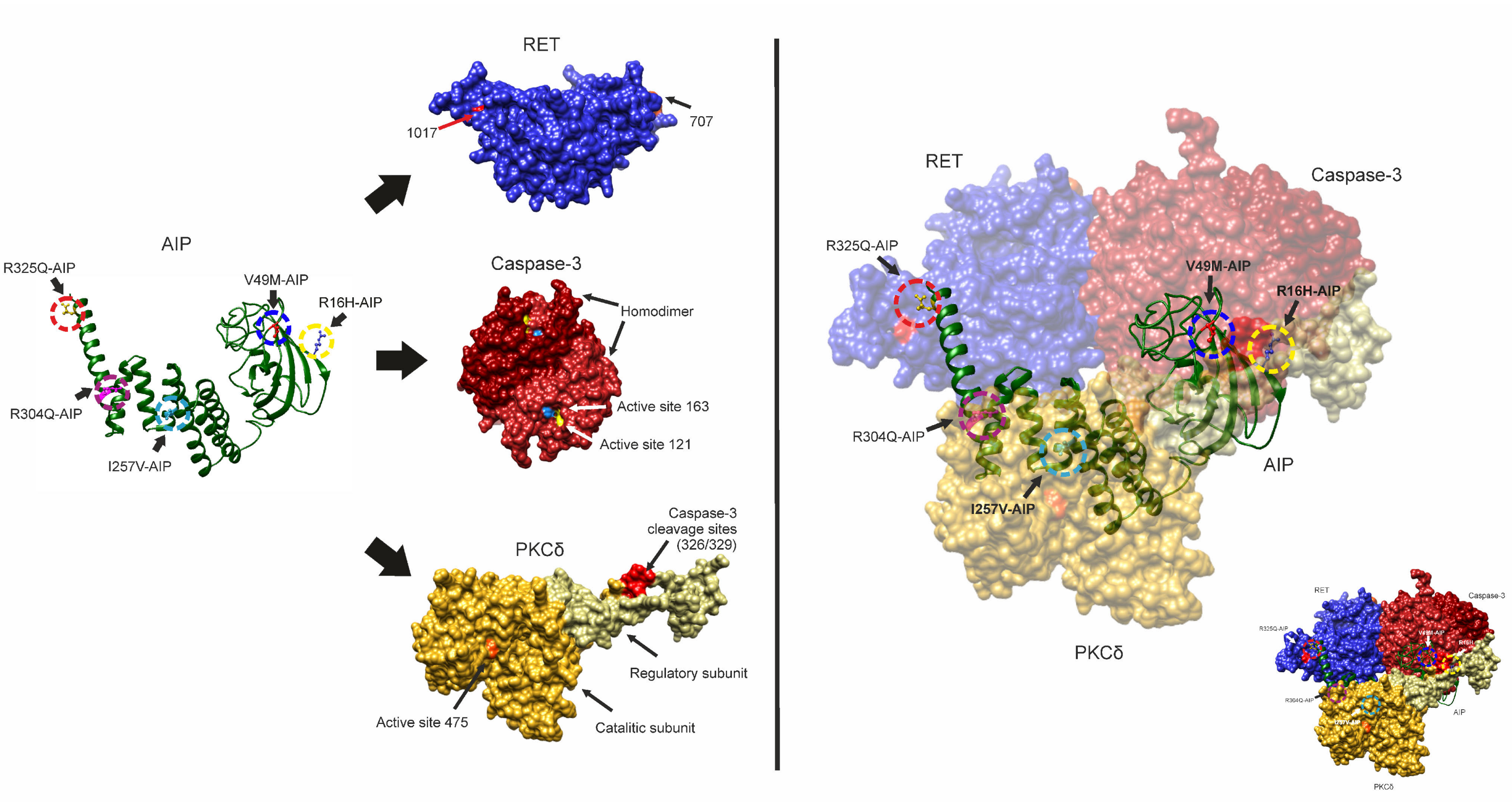

Supplementary Figure 5: Exploratory *in silico* model for RET/ Caspase-3/ PKCδ/ AIP interactions. The model was prepared with Chimera with the available 3D X-ray diffraction protein structures for caspase-3 (full-structure, homodimer), PKCδ, AIP (as a trimer, dimer or as a monomer) and RET. Many simulations were performed until the best fit was found matching results from Figure 5. **Left**: individual proteins or IC-RET fragment (705-1025 amino acids). **Right**: Transparent model (up) or solid model (down, corner).

Caspase-3 is a confirmed homodimer (structure 3 5i9b, X Ray Diffraction 1.8Å, amino acids.29-277). PKCδ has no individual structure but a model was generated based on the template PDB for PKC beta (3pfq, 230-672 amino acids, X Ray Diffraction, 4Å) and PKCδ aa sequence. RET has only homodimeric structures (4cki, X Ray Diffraction 2.12Å, amino acids.705-1013; in Chimera we eliminated one unit of the dimer, resulting in a monomer and applied to the sequence amino acids.705-1025, containing both caspase-3 consensus sites at 707 and 1017. Human AIP model was obtained from template 1qz2 (PDB) in SWISS-MODEL and the AIP sequence 8-329 amino acids. The generated model is an homotrimer based on homology proteins. We eliminated two units resulting in the AIP monomer. In our hands the exploratory model favors AIP as a monomer. RET interacts directly with Caspase-3 through the Caspase cleavage consensus sites at 707 and 1017 (stronger the 707 interaction). PKCδ interacts with Caspase-3 through the N-terminal regulatory portion (so-called regulatory subunit) where the consensus caspase site is located. AIP interacts with Caspase-3 through V49, and this interaction is lost with V49M.

AIP interacts with RET through the R325; when this is changed in R325Q, both RET and Caspase-3 are lost since RET+ Caspase-3 interaction is strong.

AIP interacts with PKCδ through three residues. R16 at the N-terminal portion (regulatory subunit) where Caspase-3 is near; this AIP-PKCδ interaction is affected in R16H; a second AIP-PKCδ interaction at the C-terminal portion of PKCd is through R304, that is lost with R304Q. The AIP-PKCδ interaction is not significantly altered in I257V, although its tendency is to be increased, while the association to RET + Caspase-3 is reduced suggesting a conformational shift in AIP that prevents RET-Caspase-3 binding.

Suppl. Figure 6 A-B-C-D

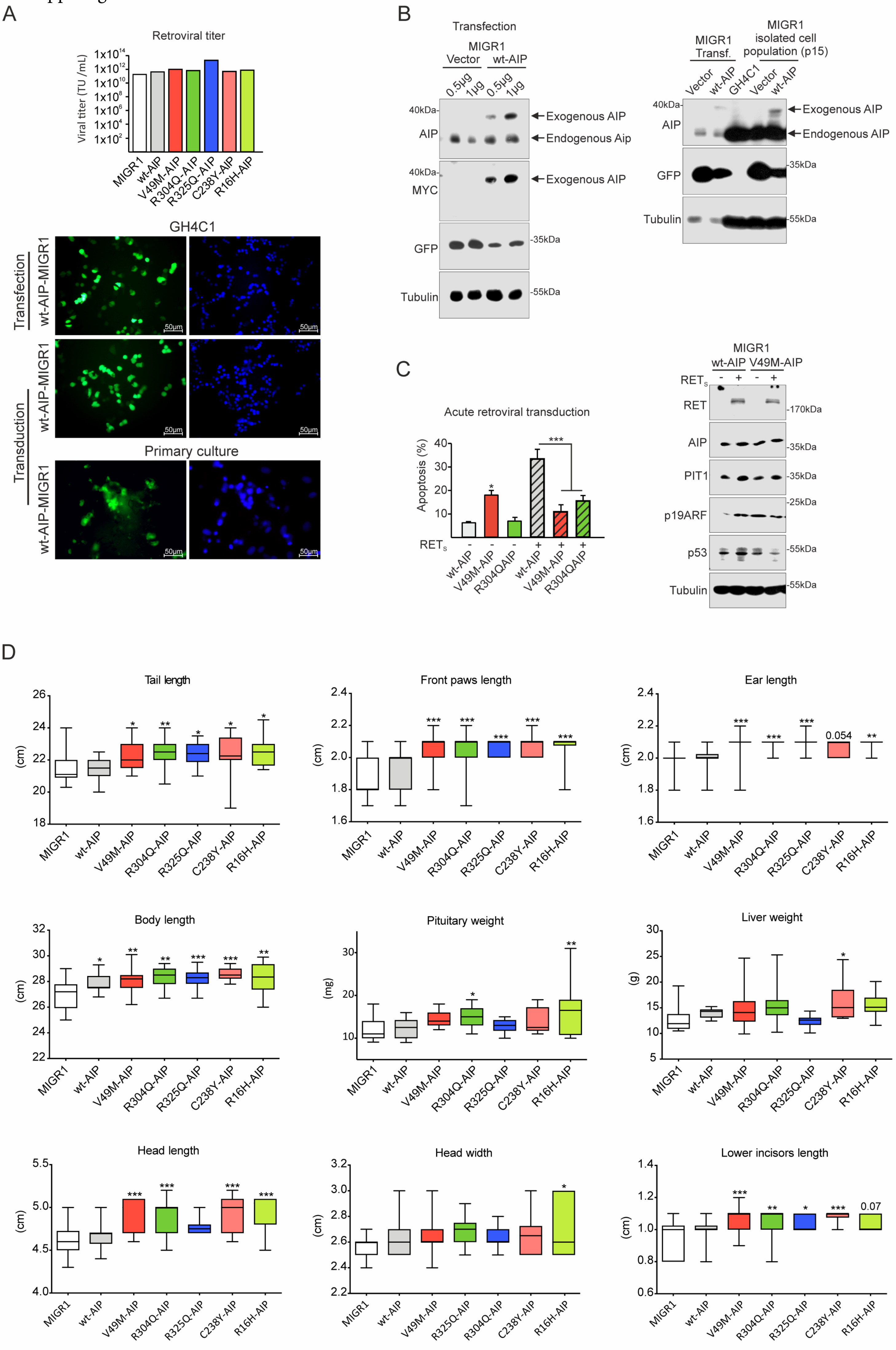

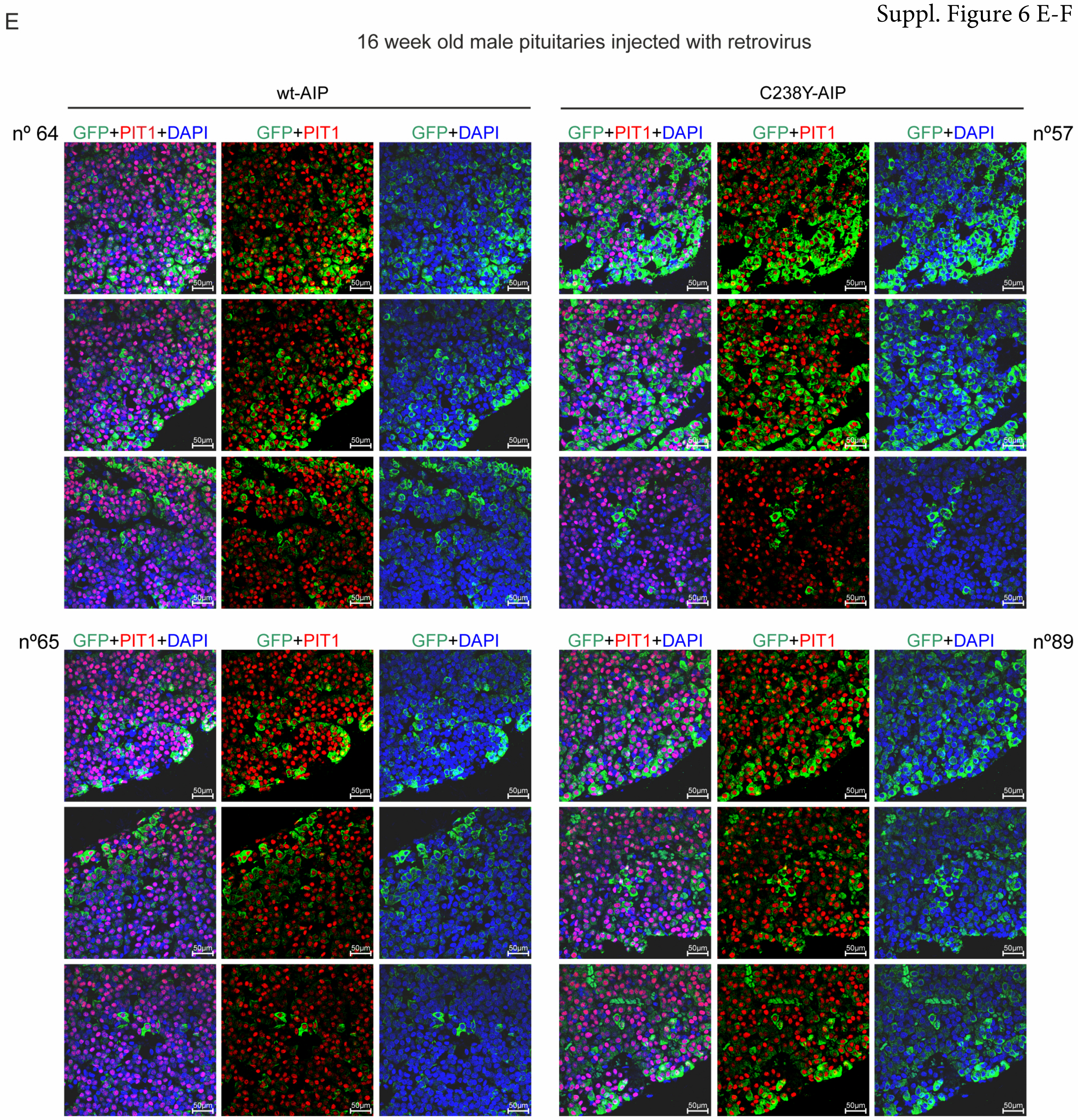
E
16 week old male pituitaries injected with retrovirus
wt-AIP
C238Y-AIP
nº 64
nº57
nº65
nº89
GFP+PIT1+DAPI
GFP+PIT1
GFP+DAPI
50μm

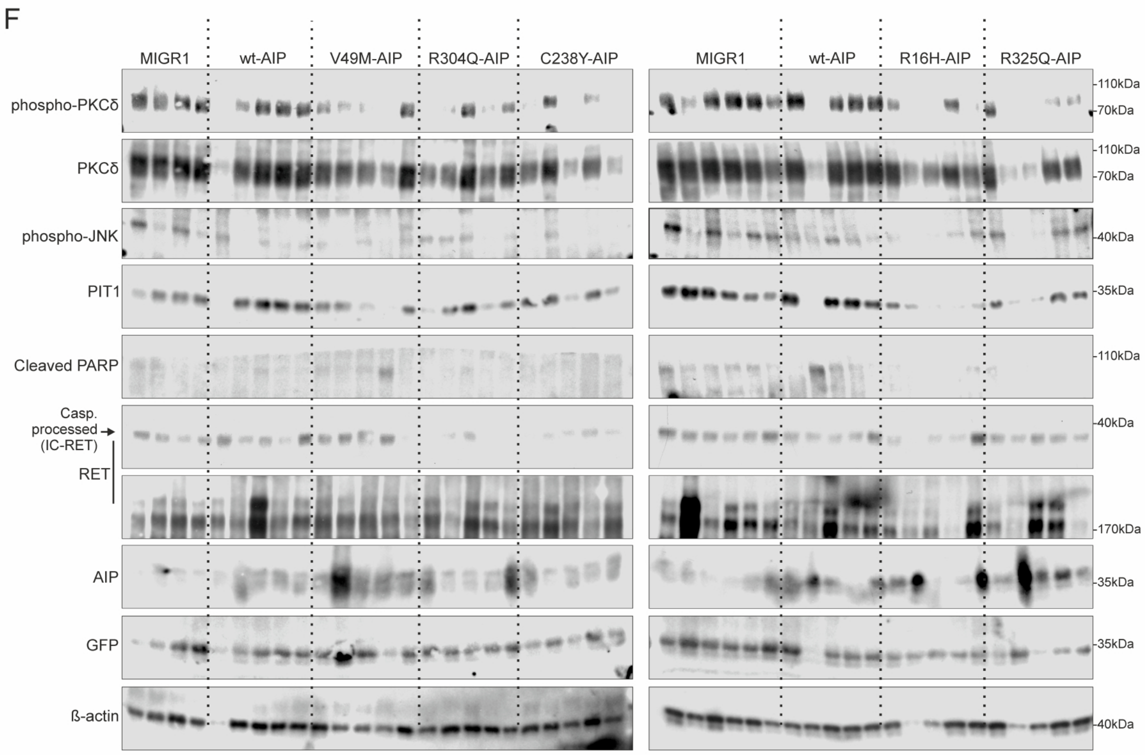
F
MIGR1
wt-AIP
V49M-AIP
R304Q-AIP
C238Y-AIP
R16H-AIP
R325Q-AIP
phospho-PKCδ
PKCδ
phospho-JNK
PIT1
Cleaved PARP
Casp. processed (IC-RET)
RET
AIP
GFP
ß-actin
110kDa
70kDa
40kDa
35kDa
170kDa

Supplementary Figure 6: Characterization of the new model of pituitary gigantism through alteration of RET-AIP interaction using AIP mutants injected at birth

A-C) Retrovirus quality controls. A) Quantitative titration using qPCR of the different retrovirus bearing vector IRES-EGFP (MIGR1) or *AIP* (wt*AIP* or variants). This was followed by a functional titration comparing plasmid transfection with retroviral transduction in HEK293T (not shown) and GH4C1. EGFP + cells (green) were quantitated over total cells (Hoechst 33258, blue). **B)** Transfection of the plasmid in specific conditions to separate endogenous rAip from exogenous myc-tagged AIP. Long-term populations of wt*AIP* or vector infected cells after 15 passages still expressed exogenous AIP. **C)** Acute transduction with wt*AIP*, V49M or R304Q followed by RETs transfection in GH4C1 cells. Variant *AIP* blocked RET-induced apoptosis and the RET/Pit1/ARF/p53 pathway.
D-F) Phenotypic characterization of male rats. Analysis of somatotroph specificity through GFP cellular co-localization in male rat pituitaries. Analysis of protein levels through western blot of whole pituitary extracts. D) Other measurements were also significantly increased in male rats infected with variant *AIP* in the pituitary, such as body length, head length -but not width and lower incisors. Regarding other organs, pituitary weight was only significantly different in two variants (R304Q and R16H) and there were no consistent differences in liver weights. **E)** In 16 weeks old male rat pituitaries, GFP (for EGFP detection) was expressed at the periphery of the adenopituitary lobes (somatotroph areas) in cells co-expressing nuclear PIT1. Different sections of two wt*AIP* (rat nº 64 and 65) and two C238Y AIP variant (rats 57 and 89) are shown. **F)** Western blot of groups (n=4) of pituitary extracts from vector (MIGR1), wtAIP or variant AIP. High throughput gels for 22 samples were used to run extracts in parallel. Quantification of the data and statistics are shown in the main article.
(C: n=4, with at least 4 replicates measurements in each. D: n is indicated in main Figure 6. E: three pituitaries were studied; shown images from two.
Two-ways ANOVA with Tukey's multiple comparison test correction, C; One-way ANOVA with Dunnett's multiple comparison test correction D. *, $p<0.05$; **, $p<0.01$; ***, $p<0.001$).

Extended Text related to the phenotype in female rats injected at birt with AIP variants (Suppl. Figure 7)

Results in female rats showed an attenuated phenotype. Although individual female animals injected with variant *AIP* presented a clear growth phenotype, when grouped, 16 week-old female rats injected at birth with any of the *AIP* variants did not present significant differences in serum levels of IGF-1, nor in body composition, nor differences in % of cells positive for cleaved caspase-3, IC-RET or p-PKCδ, although there was a tendency for reduced cleaved caspase-3 in variant *AIP* (Suppl. Fig 7A-G). Females injected with three *AIP* variants (R304Q, R325Q and C238Y) had a significantly longer body. Significant differences in GFP positivity were found in variant *AIP*-injected groups compared to wt*AIP* (Suppl. Fig 7G). However, pituitary sections and adenohypoyses areas were consistently bigger and hyperplastic in variant AIP injected rats in both sexes (Suppl. Fig 7H).

We performed correlation of all the above variables in relation to serum IGF-1 levels in male and female rats (Suppl. Fig 7I). Significant positive correlations between IGF-1 levels and phenotypic measurements were found in males and in females when selecting those female rats with IGF-1 levels >2SD over median of MIGR1 controls. There were two exceptions: in females, heart weight was not correlated with IGF-1, and fat /fat mass (MRI) were significantly inversely correlated with IGF-1 when in males, it was lean mass positively correlating with IGF-1. These results are in keeping with a sexual dimorphism in pituitary GH secretion and GH-IGF-1 effects in peripheral tissues.

We evaluated the weight curves after birth till puberty taken as a measure, albeit not precise, of growth. Just before puberty, in both sexes, weights of variant *AIP*-injected 4 week old animals were significantly larger than wt*AIP*- or MIGR1-injected animals in the majority of groups (Suppl. Fig 7J). Alive animals with AIP variant injections seemed larger than animals injected with wt*AIP* (Suppl. Fig 7J, left).

Suppl. Figure 7 A-B-C-D-E

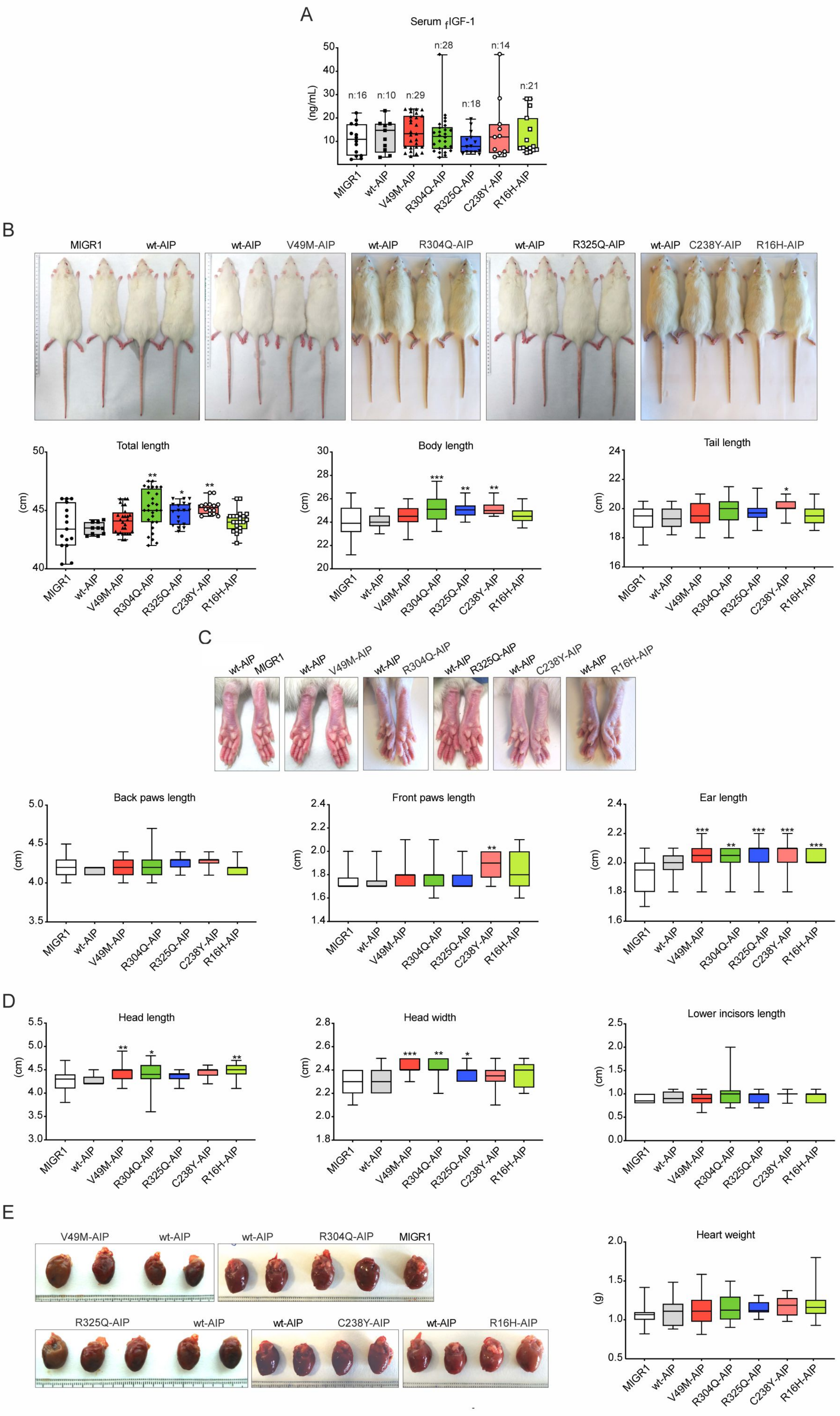

Suppl. Figure 7 F-G-H-I-J

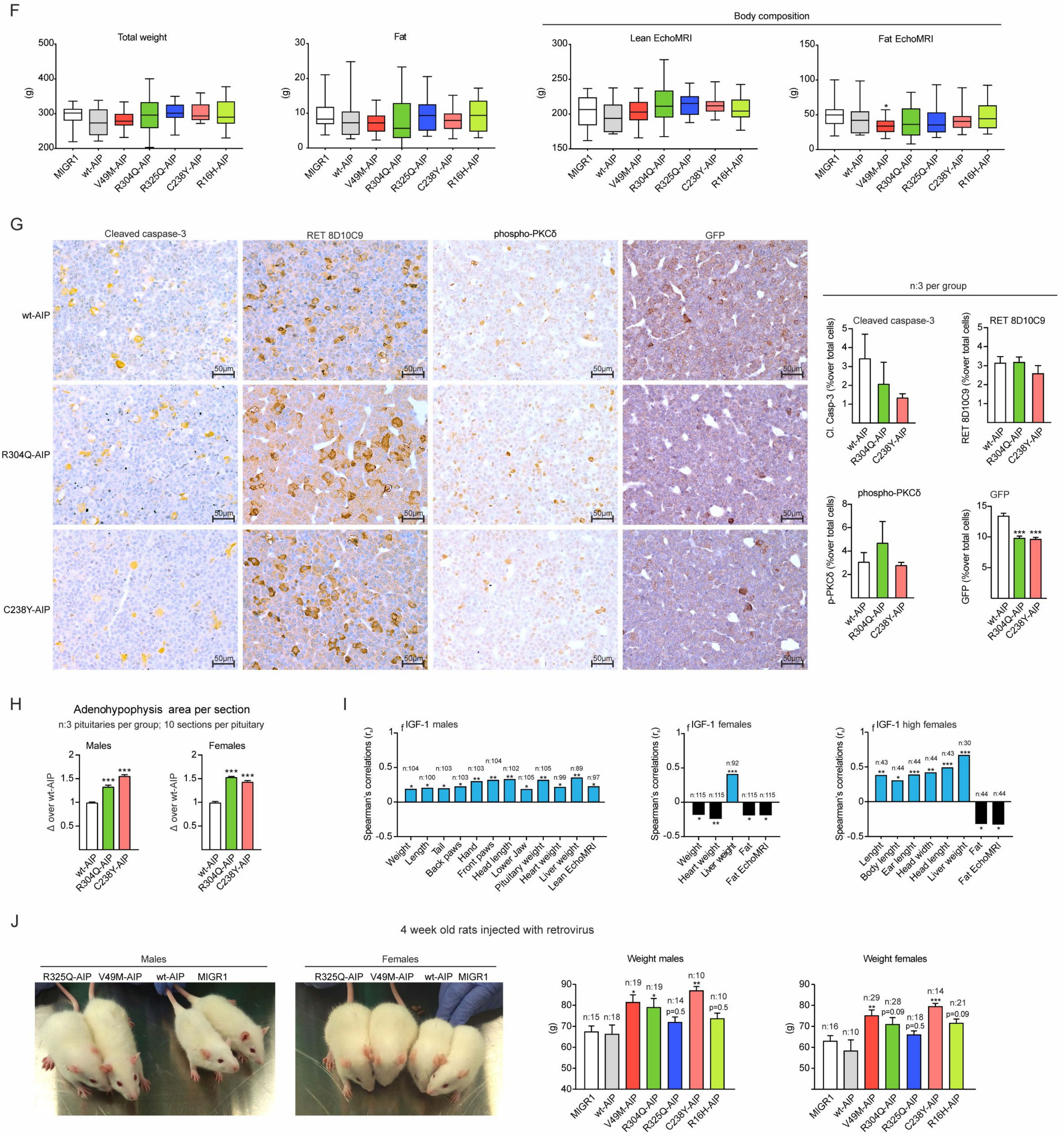

Supplementary Figure 7: *AIP* variants injected in the pituitary at birth induced an attenuated phenotype in female rats. A) Although some values in variant *AIP*-injected rats were clearly very high, free IGF-1 serum levels did not reach significance between wt or variant *AIP*-injected 16 weeks old female rats. **B)** Significant differences in total and body length were found for R304Q, R325Q and C238Y AIP variants. No significant differences were found in tail length with the exception of C238Y. **C)** In soft tissues, only ears were significantly bigger in variant *AIP* in the female rats, without significant differences in back and front paws with the exception of C238Y front paws. **D)** Heads were significantly bigger in only some *AIP* variants. **E)** Heart weights were not different. **F)** Weight and body composition was not altered in female rats. **G)** In immunohistochemistry, we found a tendency for reduction of % of cleaved caspase-3 in variant *AIP* pituitaries (R304Q, C238Y) respect to wt*AIP* pituitaries, that did not reach significance. IC-RET and p-PKCδ stained cells did not present differences. The % of GFP cells was half of those found in males. Moreover, there were slight but significant reduced GFP in the variant pituitaries. **H)** The mean adenohypophysis area per section was significantly increased in both males and females injected at birth with R304Q and C238Y *AIP* variants compared to wt*AIP,* indicating bigger pituitaries in both sexes with altered AIP. **I)** Significant correlations between IGF-1 levels and phenotypic measurements in all male (left) and female (center) rats show differences. When selecting those female rats with >2SD IGF-1 levels over median MIGR1 controls (right), similar positive correlations as in males were found with two exceptions: Heart weight was not correlated with IGF-1; and in females Fat was inversely correlated with IGF-1 when in males Lean mass was directly correlated with IGF-1. **J)** Apparent size seems bigger in male and female prepubertal 4 week old rats injected with variant AIP compared to wtAIP or MIGR1. Body weights at this age were also significant.

(One-way ANOVA with Dunnett's multiple comparison test correction A-B-C-D-E-F-H-J; Kruskal-Wallis with Dunn's multiple comparison test correction G; Spearman r correlations I. *, p<0.05; **, p<0.01; ***, p<0.01; numbers, when p is non-significant but approximates to significant levels).

Extended Text related to the bioinformatics performed on the data retrieved from ReGEO GSE63357 (Suppl Figure 8)

We performed bioinformatics on the data retrieved from the ReGEO database, code GSE63357 (Hernandez-Ramirez,L.C. et al, Oncotarget, 2018). An RNA expression profiling array comparing normal pituitary (n=5), AIP-FIPA somatotroph adenomas (n=6), non-AIP-FIPA somatotroph adenomas (n=3), and sporadic somatotroph adenomas (n=4). We retrieved log2 and significance for *RET, GDNF, GFRA1 , PIT1, CDKN2A* and *TP53* expression data in the four groups of samples. Volcano plots comparing *AIP*-FIPA (AIPpos), *non-AIP*-FIPA (AIPneg), or sporadic (SP GH), somatotrophinomas to Normal Pituitary (NP) revealed that neither of the five genes (*RET, GDNF, GFRA1, PIT1A* or *TP53*) appeared in the significant range (Suppl. Fig 8A-D). LogFC and p-values are shown in Suppl. Fig 8E. There were no differences between the different types of somatotrophinomas for those five genes (Suppl. Fig 8E).

The *CDKN2A* gene has two different isoforms, *p14ARF* and *p16*, translated into two different proteins (respectively, *p14ARF* is Q8N726 and *p16* is P42771) due to a different translational frame since each has a specific Exon 1. In the Affymetrix array *CDKN2A* probe set is represented by three probes, each having 11 oligonucleotides grouped (Suppl. Fig 8F). Only 209644_x_at probe contains oligos for the specific *CDKN2A-ARF* Exon 1. LogFC and p-values for the *CDKN2A* probe set are shown in Suppl. Fig 8E. Only the 209644_x_at probe reach shows reduced expression in *AIP*-FIPA compared to sporadic adenomas.

A B C D

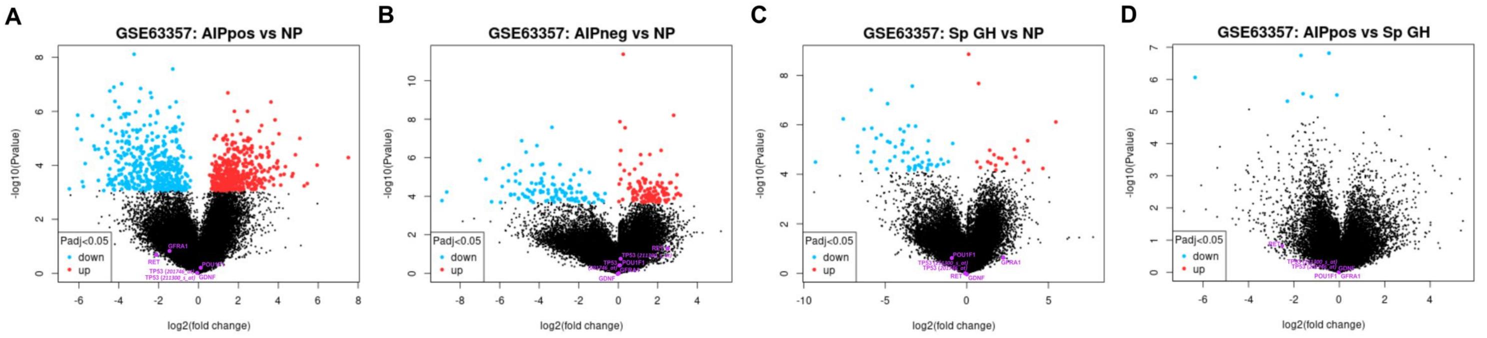

E

	RET		*GFRA1*		*GDNF*		*POU1F1*		*TP53* 211300_s_at		*TP53* 201746_at	
	logFC	*pvalue*	*logFC*	*pvalue*	*logFC*	*pvalue*	*logFC*	*pvalue*	*logFC*	*pvalue*	*logFC*	*pvalue*
**AIPneg *vs.* NP**	2.81	0.36	0.892	0.71	0.000768	1	1.01	0.60	-0.019	0.21	0.029	0.61
**AIPpos *vs.* NP**	-2.44	0.32	-1.56	0.20	-0.000768	1	1.32	0.35	0.007	0.37	0.19	0.11
**Sp GH *vs.* NP**	-0.963	0.44	2.2	0.31	0.000768	0.83	-1.14	0.16	-0.01	0.21	0.02	0.61
**AIPpos *vs.* Sp GH**	-2.42	0.054	0.63	0.09	0.0	1	-0.183	0.66	-0.012	0.51	0.022	0.14
**AIPneg *vs.* AIPpos**	0.0831	0.82	-0.473	0.82	0.00873	0.82	-0.229	0.51	-0.007	0.49	-0.11	0.44

F

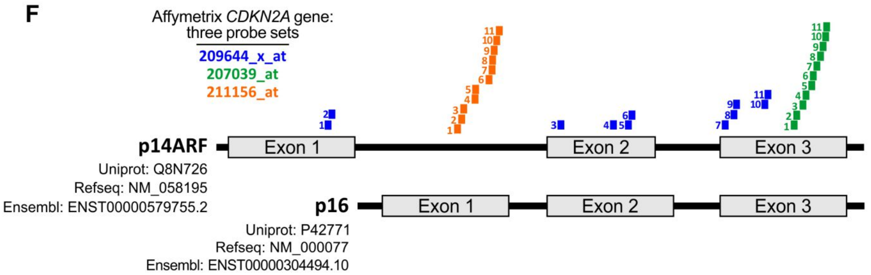

G

	CDKN2A 209644_x_at		*CDKN2A* 207039_at		*CDKN2A* 211156_at	
	logFC	*pvalue*	*logFC*	*pvalue*	*logFC*	*pvalue*
**Sp GH *vs.* NP**	-0.30	0.66	1.01	0.19	0.0	1.0
**AIPpos *vs.* Sp GH**	-2.03	0.08	-1.62	0.12	0.06	0.46

Supplementary Figure 8: Quantitative mRNA expression of RET, GDNF and GFRA1 do not differ between normal adenopituitary (NP), sporadic somatotroph adenomas (Sp GH), *AIP*-FIPA somatotroph adenomas (AIPpos) and *non-AIP*-FIPA somatotroph adenomas (AIPneg).

Data from ReGEO GSE63357 were retrieved and analysed (http://www.regeo.org/details.jsp?gseId=GSE63357). The experiment, published in Barry et al., Oncotarget 2018, compared RNA expression profiling in 5 normal pituitaries, 4 sporadic somatotroph adenomas, 6 AIP-FIPA somatotroph adenomas and 3 non-AIP FIPA somatotroph adenomas using the Affymetrix human Gene Chip HG-U133 Plus 2.0 array.

A-C) Volcano plots showing expression results for each individual adenoma group (AIPpos, AIPneg, Sp GH) compared to the NP. *RET*, *GDNF*, *GFRA1*, *PIT1* and *TP53* gene dots are marked in each plot (violet). **D)** Volcano plots comparing the AIP-FIPA (AIPpos) and Sporadic (Sp GH) adenomas groups among themselves. *RET*, *GDNF*, *GFRA1*, *PIT1* and *TP53* gene dots are marked in each plot (violet). **E)** Quantitative data showing no significant differences among the groups for the genes in the pathway *RET, GDNF, GFRA1, PIT1* and *Tp53*.

F) The *CDKN2A* probe sets in the array is comprised of three probes. Scheme indicating the localization of the hybridization regions for each of the three spots/ probes, each containing 11 probes within the same region, representing the CDKN2A gene contained in the Affimetrix human Gene Chip HG-U133 Plus 2.0 array: 209644_x_at, 21156_at and 207039_at. Only 209644_x_at contains probes specific for the specific Exon 1 of *CDKN2A-p14ARF*, corresponding to the protein Q8N726, although it. The probe 21156_at is addressed to the alternative Exon 1 specific for the *CDKN2A-p16* isoform, corresponding to the protein P42771. The probe 207039_at corresponds to the common Exon 3. Proteins Q8N726 and P42771 are translated with a different frame, and have no common sequence. **G)** Quantitative data showing a difference of -2.03 log2 in FC for probe 209644_x_at with p=0.088, while none of the other two probes nor presented FC values over 2 nor reach a p value lower than 0.1.

Supplementary Figure 9 or Graphical Abstract.

The role of AIP in the survival and apoptotic RET pathways in normal conditions (A) and when AIP is lacking (B)

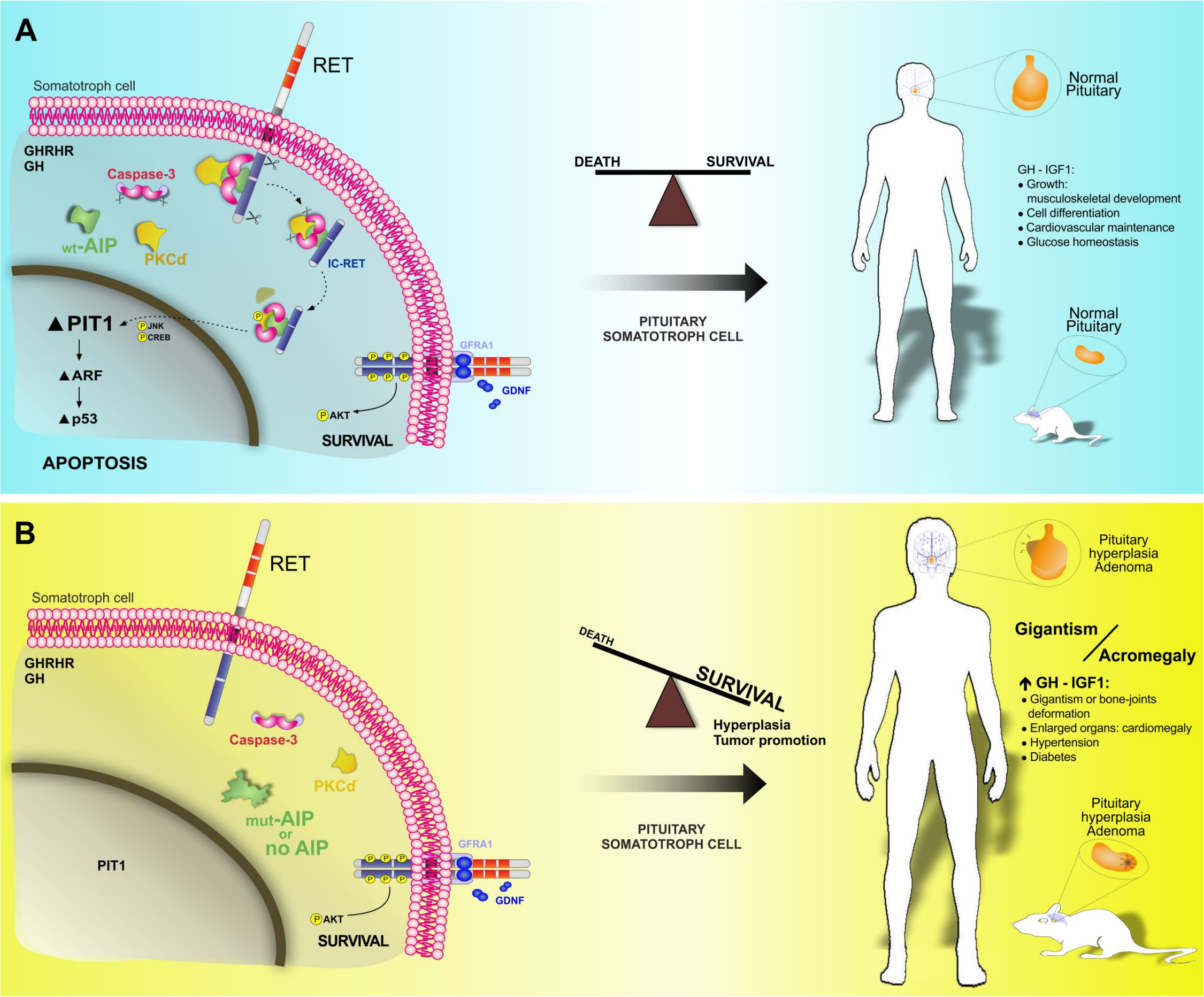

Supplementary Information:

Extended Material and Methods **page 27**

Suppl. Methods Tables 1-6 **page 39**

References **page 53**

In silico study of AIP variants

The different types of human AIP variants were analysed using Ensembl [1]. The clinical significance of the variants was analysed using VarSome [2], ClinVar [3], dbSNP [4] and Ensembl variant effect predictor (VEP) [5].

To evaluate the potential pathogenicity of the AIP variants we use free software with specific prediction algorithms related to gene sequence, protein sequence and domains, or allele frequency at population level. The first group of programs include more than one criterion such as the CADD2 v1.6 [6] that merges evolutionary conservation information with functional information; REVEL[7] that integrates thirteen scores; and MetaIR [8] which combines deleterious variants with the frequency of the alleles in the population. The second group of programs based on the structure and function of the protein were: PoPMuSiC [9], HoTMuSiC [10], SNPMuSiC [11], SIFT [12], FATHMM-XF [13], PolyPhen2-HDIV [14], PolyPhen2-HVAR [14], PROVEAN [15] and MutationAssessor [16]. The third group of programs based on minor allele frequency (MAF) to distinguish between common polymorphisms and rare variants (MAF <1%) are: GnomAD [17] and ExAC [18].

Thresholds for normality were given by each program. Recently a second threshold has been proposed for some of the above algorithms [19]. Variants were revised by both threshold criteria as shown in Suppl. Table 2: red or carnation colour code when pathogenic/ likely pathogenic respect to threshold; underlined when pathogenic/ likely pathogenic respect to alternative threshold.

Cell lines and rat adenohypophysis primary culture

The rat pituitary cell line GH4C1 was cultured in DMEM (D6046, Sigma) plus 10% FBS (10270-106, Gibco) and 1% of Penicillin-Streptomycin solution (P0781, Sigma). HEK293FT cell line was cultured in DMEM (D5796, Sigma) plus 10% FBS, 1% of Penicillin-Streptomycin solution, 5X L-glutamine (25030-024, Gibco), 5X MEM NEAA (11140-050, Gibco) and 5X Sodium Pyruvate (P5280, Sigma). All the cells were maintained at 37ºC and 5% of CO_2.

Identification of cell lines was performed following ICLAC protocols (https://iclac.org/). In rat GH4C1 phenotype was used as the main tool (See Supl. Fig. 1). HEK293T were identified through STRs (Applied Biosystem Identifier Plus Panel v1). Our profile was >95% identical to the one on the Cellosaurus database corresponding to DSMZ from where they were obtained some years ago:

Applied Biosystem Identifier Plus Panel v1	HEK293T (at our institute CIMUS)	HEK293T Cellosaurus DSMZ Profile
D8S1179	12,14	12,14
D21S11	28,30.2	28,30.2
D7S820	11	11
CSF1PO	7,12	11,12
D3S1358	17	15,16,17
TH01	7,9.3	7,9.3
D13S317	12,14	12,14

D16S539	9	9
D2S1338	19	19
D19S433	15,18	15,18
vWA	16,19	16,19
TPOX	11	11
D18S51	17,18	17,18
AMEL	X	X
D5S818	8,9	8,9
FGA	23	23

Adenohypophysis cells were cultured as described [20, 21, 22]. Two-week-old (30-50g) *Sprague-Dawley* male rats were decapitated, and the pituitary gland removed. In a petri dish under a culture hood, adenohypophysis was dissected and kept in DMEM. When all the pituitaries were dissected, DMEM was discarded and adenopituitaries minced with a surgical blade. Minced glands were digested with a solution of 0.4% (w/v) type-IV collagenase (C5138, Sigma), 0.2% (w/v) dispase II (165859, Roche), 0.1% (w/v) hyaluronidase (H3506, Sigma), 0.01% (w/v) DNAse I (DN-25, Sigma), and 10% (v/v) FBS in DMEM, at 37°C for 20 min with mechanical dispersion at 5 min intervals. The resulting cell suspension was centrifuged, and the pellet resuspended in 1.5 mL of trypsin-EDTA 1X (T4174, Sigma) for 2-5 minutes. DMEM+10% FBS in was then added, and the cells were centrifuged and washed once with DMEM. The cells were finally re-suspended in Semisynthetic Medium (MSS) modified from Soto et al[23]. MSS is composed by Ham's F12/DMEM medium (F12:DMEM 6.5:3.5 v/v) containing 2 g/L BSA (A7906, Sigma), 2.4 g/L HEPES (H3375, Sigma), 143 µg/L hydrocortisone (H0888, Sigma), 0.4 µg/L T3 (T6397, Sigma), 10 mg/L Apotransferrin (T1147, Sigma), 10 ng/L glucagon-like peptide (G9416, Sigma), 0.1 µg/L epidermal growth factor (E9644, Sigma), 0.2 µg/L fibroblast growth factor (F3133, Sigma), 10^{-9}M ([D-Ala2]-Growth Hormone Releasing Factor 1-29 amide human (SCP0161, Sigma) 10% FBS, 1% Penicillin-Streptomycin, and 100 µM FURDR (F0503, Sigma) to prevent fibroblast growth. Cells were plated in Corning culture dishes coated with Poly-L-Lysine (P4707, Sigma), then incubated for 2 days.

In vitro experimental design

Transient transfections in cell lines were performed as described [20, 21] using Nucleofector™ IIb (Amaxa AAB-1001, LONZA) with A-020 protocol, kit L, VCA-1005, (Lonza) for GH4C1, and A-023, kit V, VCA-1003, (Lonza) for HEK293FT. Cells must be actively dividing for an efficient transfection with this method, so the cells have to be trypsinized 2 days before starting the procedure. wtAIP or AIP variants were cloned in pcDNA3.1(+) (Thermo) with an N-terminal Myc tag.

RETS, RETL and IC-RET have been published in [20]. All plasmids and siRNAs are listed in Suppl. Methods Table 1. Plasmids are available upon request.

Plasmid mix was prepared in 5 µl TE (quantity was established after standardization by western blot, respectively. 3.5 µg RET, 0.25 µg AIP, 3.5 µg pcDNA3.1, 3 µg IC-RET, 0.5 µg PKCd, 0.8 µg siRNA). After trypsinization, 1.2 10^6 cells were pelleted and resuspended in 100 µl of the kit solution and mix with plasmid solution into the cuvette. After electroporation, cells were resuspended in 6 mL complete medium and seeded in 6-well plates at 10^5 cells/well, 24-well plates at 50.000 cells/well or 48-well plates at 35.000 cells/well.

For randomization, cells were seeded in multiwells by lines, and transfections or treatments were performed by columns, or vice-versa.

Alternatively, cells were transfected using TurboFect Transfection Reagent (R0531 Thermo Fisher) for GH4C1, HEK293FT and rat pituitary primary cell cultures. For 24-well plates 1 µg total DNA (AIP variants or AIP siRNA) was mixed with 2 µl Turbofect, and 100 µl of the mix were added to each well. For 48-well plates 0.5 µg total DNA (AIP variants or AIP siRNA) was mixed with 1 µl Turbofect, and 50 µl of the mix were added to each well. For 96-well plates 0.4 µg total DNA (AIP variants) was mixed with 0.7 µl Turbofect, and 40 µl of the mix were added to each well. For 6-well plates, 4 µg total DNA (RET, AIP variants in IRES construct) was mixed with 6 µl Turbofect, and 400 µl of the mix were added to each well.

After transfection cells were kept in full medium for 36 hours. Experiments were performed in serum *deprived* conditions using DMEM + 0.1% BSA (A7906, Sigma) for GH4C1 and DMEM + 0.5% FBS for HEK293FT or MSS + 0.5% FBS for adenohypophysis primary cultures after washing the cells one time with PBS. Under deprived conditions cells were treated with vehicle or rat GDNF (PF039, Calbiochem) 50 ng/mL for GH4C1 or 100 ng/mL for primary cultures. This recombinant GDNF is obtained from Sf9 fly cell line, (>97%) and with very low endotoxin contain (LPS ≤1.0 EnU/µg).

Plasmid preparation, Apoptosis assays and cell counting

Apoptosis detection was performed and counted 48 hours after treatments as described [20, 21, 24, 25] In brief, 1 µL/mL of Hoechst H33258 (bisbenzimide, B2883 Sigma, stock 50 µM in distilled water) prediluted 1:10 in culture medium was added to the cells. After 45 minutes of incubation, the number of apoptotic cells versus the number of total cells is counted after photographing six fields/ well in the fluorescence microscope (Olympus IX70/IX51, Objective CPlanN 10x) with CellSensv1,16 software. Hoechst H33258 binds to DNA staining all living nuclei in a dark blue colour in cells. Apoptotic cells present condensed chromatin with a bright light-blue colour. For each well at least 6 different fields totalling >400 cells were counted, with 4 replicates per condition. At least three independent experiments for each AIP variant -/+ RET condition were performed.

As an alternative method to detect earlier apoptosis, Caspase-3/7 CellEVENT (Thermo) was performed by adding 8 µl/ 100 µl of medium. Hoechst 33258 was also added. After 30 min in the incubator (dark) fluorescence was registered with the above fluorescence microscope.

For cell counts, parallel experiments were performed but, following a time-course, cells were trypsinized and counted in a cytometer. At least three independent experiments for each AIP variant -/+ RET condition were performed with 4 replicates per condition.

Lysates, subcellular fractionation, western blot and immunoprecipitation

Experimental setup regarding plasmid combination at transfection and protocol for lysates is summarized in Suppl. Methods Table 2.

Total cell extracts, including nuclear proteins, at long term (48 hours) were obtained with SDS-Lysis Buffer, by adding 15 µL of boiling 1% SDS per 6-well plate as described [25, 26 ;27, 28]. After scraping, lysates were incubated at 95ºC for 5 min in a thermoblocker, and diluted 1:4 with Lysis Buffer (50 mM Hepes pH 7.4 (H3375, Sigma), 1% Triton X-100 (T8787, Sigma), 10% Glycerol (G9012, Sigma), 150 mM NaCl (S9888,Sigma), 5 mM EGTA (E5134, Sigma), 1.5 mM $MgCl_2$ (M8266, Sigma), 20 mM Na-pyrophosphate (S9515, Sigma), 2 µg/mL Aprotinin (A6279, Sigma), 4 mM PMSF (P7626, Sigma) and 92 µg/mL Na_3VO_4 (S6408, Sigma). For cytosolic short-term extracts (30 minutes, 1 hour) we used 60 µL Lysis Buffer directly (without SDS) per 6-well-plate [20, 29].

Subcellular fractionation was performed in 6-well plate, following the protocol described by P.Holden and W.A. Horton [30] with some modifications. After intensive washing with cold PBS, we add 100 µL buffer 1 (Digitonin buffer) directly to the well. This volume was maintained for buffer 2, 3 and 4.

For immunoprecipitation 100-150 µg of protein (cytosol extracts and subcellular fractionation extracts 1 and 3) were mixed with 0.4 µg of Myc or PKCδ antibodies. For the negative controls, the same amount of protein extract was used without antibody. 30 µL of G-Sepharose Gamma Bind (17-0885-01, GE Healthcare) was added to each sample and incubated for 1 hour and 30 minutes at 4°C in an end-to-end mixer. Samples were washed 6 times with cold HNTG buffer (20 mM Hepes pH7.5, 15 mM NaCl, 10% Glycerol, 1% Triton X-100) and centrifuged at 12000 rpm for 2 minutes at 4°C.

Lysates from rat adenohypophysis were prepared in 0.5 mL/ 10 mg tissue of Tissue Lysis Buffer (50 mM Hepes pH 7.4, 10 mM NaCl, 1% Triton™ X-100, 10 mM EDTA pH 8 (E9884, Sigma), 10 mM NaF (S7920, Sigma), Cocktail *complete*-EDTA-free (11873580001, Roche), 2 µg/mL Aprotinine, 4 mM PMSF, 92 µg/mL Na_3VO_4 and 10 mM Na-Pyrophosphate). Tissue was homogenized by mechanical action in a TissueLyser II (85300, Qiagen) at 30 rpm for 3 minutes followed by incubation on wet ice for 15 minutes and centrifuged at 12000 rpm for 30 minutes at 4°C.

To quantify total proteins in each lysate we used Pierce Protein Assay™ 660nm (22660, Thermo Fisher Scientific) and ICDR (Ionic Detergent Compatibility Reagent, 22663, Thermo Fisher Scientific) following manufacture protocols. This last method allows measuring in lysates containing ionic detergents and reducing agents, which can interfere in the quantification for the Pierce method.

50 µg cell lysates were loaded per lane in 6, 10,12 or 13% SDS-PAGE in GE Healthcare miniVE Vertical Electrophoresis System (80-6418-77, Fisher Scientific) with Page Ruler Prestained Protein Ladder (26616, Thermo Fisher Scientific). Transfer to the membrane (Immun-Blot PVDF 0,2 µm, 162-0177, Biorad) was performed using semi-dry blotter system (Z34 050-2, Sigma).

20 µg adenohypophysis extracts were run in parallel using Precast Criterion™ TGX™ (Tris-Glycine eXtended, 56710-25/35/45, Biorad) in Criterion™ Vertical Electrophoresis Cell (1656001, Biorad).

Antibodies and dilutions are showed in Suppl. Methods Table 3. Enhanced chemiluminescence (ECL) assay (Pierce) was used for detection (32106, Thermo Scientific). Membranes were exposed to photographic films (Super RX-N 47410 19289, Fujifilm) and exposed for 2.5, 10, 30 minutes or for hours. For densitometry in western blots we use Quantity One 1-D Analysis Software (Biorad).

Caspase-3 enzyme activity assay in cell lysates

Transfected cells were seeded at 20.000 cells per well in a 96 well-plate, grown for 36 hours, deprived and treated with 50 µL vehicle or 20 µM of Caspase-3 inhibitor Ac DEV-CHO (A0835, Sigma) for 2 hours. After this, cells were lysed with 50 µL Caspase-Glo® 3/7 Assay kit (G8090, Promega) for 45 minutes in an orbital shaker at low mode. Lysates were centrifuged for 5 min at 1000 rpm. Luminescence was measured in the supernatants in a white plate using the Multimode Mithras LB 940 (Berthold Technologies). At least three independent experiments for each AIP variant -/+ RET condition were performed with 4 replicates per condition.

Promoter activity

Pit1 promoter activity was measured using -378 sequence of the rat Pit1 promoter (Suppl. Methods Table 1) together with pSV-β-galactosidase in GH4C1 cell as described in [20, 21, 22] by the Amaxa method.

0.5 µg of p-378Pit1-luc and 0.25 µg of pSV-β-galactosidase were co-transfected with 3.5 µg of RET_S (pcDNA3- RET_S) or the empty vector; and with 0.25 µg of wt-AIP or *AIP* variants; or 0.8 µg of rat Aip siRNA or non-target Scramble siRNA (Sc); Suppl. Methods Table 1) by Amaxa. 50.000 cells/well were seeded after transfection in a 24-well plate.

After 36 hours, cells were deprived during the appropriate time. Wells were washed with cold PBS and lysates obtained in 80 µL 5X Passive Lysis Buffer (E194A, Promega). Luciferase and β-galactosidase activities were assayed in 40 µL of lysate as described in [20, 21, 26] using Multimode Mithras LB 940 (Berthold Technologies). Results are expressed as relative increment over control. At least three independent experiments for each AIP variant -/+ RET condition were performed with 4 replicates per condition.

RNA extraction and qRT-PCR

RNA was extracted from cells and tissues using TRIzol™ reagent (Invitrogen, 15596026) and following the indications described by the supplier. To characterize the GH4C1 line, the adenohypophysis of 10 *Sprague Dawley* rats (5 males and 5 females, USC Animal Center) and 10 *Wistar Han* rats (5 males and 5 females, Janvier Labs) as well as five different passages of GH4C1 cells were extracted. RNA was QC and quantified using Nanodrop 2000 spectrophotometer (ThermoFisher).

1 µg of total RNA was treated with 1IU RNAse free DNAse (EN0521, Thermo Fisher), 5 µL of 10X Buffer and water to a final volume of 50 µL at 37ºC for 30 minutes [31]. To stop the reaction, 5 µL of 50 mM EDTA were added and incubated for 10 min 65º C. cDNA was then obtained by RT-PCR using 1.5 µL de 300 UI MMLV (28025-013, Invitrogen, USA), 6 µl 5X First-Strand, 3 µL 0.1M DTT, 1 µL RNaseOUT™ Recombinant Ribonuclease Inhibitor (40 U/µL). 1.5 µL 10mM dNTPs, 0.1 µL random primers and distilled water to a final volume of 30 µL [31]. RT-PCR protocol: 25ºC for 10 minutes, 37ºC for 50 minutes, 42ºC for 30 minutes and 95ºC for 5 minutes.

qPCR was performed using 1 µL of cDNA with 6 µL of 2X TaqMan Gene Expression MasterMix (4369016 Applied Biosystems) and 4 µL of diluted primers (Suppl. Methods Table 4) in a 7500 Real-Time PCR System (4351105, Applied Biosystems).

Each 96-well-plate was used for a single reaction that includes samples (2 replicates) together with positive and qPCR negative controls (all reagents except for MMLV) and blanks (all reagents except cDNA)[31]. As control of expression for quantitative assays, TBP is used, since it shows stable expression pattern in different tissues and cell lines [32] including endocrine tissues [25, 31]. For expression we used ΔCt (comparative GH4C1 cells to adenohypophysis tissue) or ΔΔCt [25, 31].

Proximity Ligation Assay (PLA)

The *in situ* technology Duolink® Proximity ligation Assay (PLA) from Sigma was used for the evaluation of the interaction between two proteins in their native state and for the quantification of this interaction by the number of dots (positive interaction) per cell. RET_S was equally co-transfected in all required experiments while Caspase-3 was endogenous. To avoid misinterpretation of the data due to differences in the expression efficiency of *AIP* variants, bicistronic MIGR1-myc-AIP plasmids that co-express EGFP were transfected (see below). This allows us to quantify the positive interaction of different AIPs with other proteins in relation to the intensity of EGFP expressed by each transfected cell.

20.000 cells per well were seeded in a black glass bottom 96 well-plate (655892, Greiner bio-one). After 24 hours the cells were transfected using 0.7 µL/well Turbofect (Thermo) and 0.2 µg MIGR1-myc-*AIP* construct, 0.2 µg pcDNA3- RET_S or pcDNA3.1, in a final volume of 40 µL of DMEM. 36 hours later, wells were serum-deprived for 1 hour in 0,1% BSA+DMEM and were fixed with 100 µL of 10% Neutral Buffered Formalin (05-K-01-009 Bio-Optica) for 10 minutes at room temperature. The wells were aspirated carefully and 100 µL of MetOH at -20°C was added for 7 minutes. Each well was washed twice with cold PBS and 100 µL of 0.1% Triton-X100 was added for 1 hour on wet ice. The wells were washed 1 time with cold PBS and the residual volume was completely discarded. To each well 50 µL of Blocking solution (DUO82007, Sigma) was added for 30 minutes at 37°C. During this time, pairs of primary antibodies (combination of rabbit- and mouse-primary antibodies, Suppl. Methods Table 5) were added in 50 µL per well of Antibody Diluent (DUO82008, Sigma) and incubated overnight at 4ºC.

The procedures of washing, hybridization with the anti-Rabbit PLUS (DUO92002, Sigma) and the anti-mouse MINUS (DUO92004, Sigma) and the ligation and signal amplification with the Duolink® *In Situ* Detection Reagents Red (DUO92008, Sigma) were carried out according to the procedure described by the supplier. In the final step, 50 µL of Fluoro-Gel, (17985-10, Electron microscopy Sciences) with 2 mg/mL of DAPI (D9542, Sigma) were added to each well and incubated for 10 minutes at room temperature in darkness. Carefully 200 µL of 80% glycerol was added to each well and the multiwell plate was stored at -20ºC until analysis.

Wells were photographed with Leica TC SP5-AOBS broadband confocal microscope equipped with a white laser (470-670 nm) using 63X oil objective (NA=1.5) and the LAS AF software (Leica Application Suite Advanced Fluorescence). Maximum projections were generated from serial plans of 0.7 µm including 3 different channels: red (dots, 594 nm excitation, 624 nm emission), green (EGFP, excitation 489 nm, emission 509 nm), and blue (DAPI, excitation 358 nm, emission 461 nm). At least 6 to 10 fields of each replicate were made and 3 independent replicates for condition with a median number of >100 cells/replicate analysed.

Once the images were obtained, Fiji-Image J 1.51 [33] software was used to group the different channels of the images in a suitable format for analysis. CellProfiler 3.1.8 [34] software was used to quantify the images using our own designed pipeline (Pipeline_Garcia_Redueles_et_al_2020) Results on total number PLA dots, the integrated intensity of EGFP per cell, total number of cells, number of PLA dots in each cell were obtained and represented as the number of PLA dots in relation to integrated intensity of EGFP in each cell.

I*n silico* exploratory model for protein interaction

An exploratory model of the interaction between AIP, RET_S, PKCd and Caspase-3 based on the results of the PLA assay was carried out with two software's SWISS-MODEL [35] and UCSF Chimera [36].

SWISS-MODEL obtains different 3D structure models of a protein using as template the protein sequence and data collected from known crystallographic structures, either from this protein or from homologous proteins, of the Protein information repository Data Bank (PDB). These generated models represent the complete protein or partial regions, as well as their most likely oligomeric state. Afterwards, UCSF Chimera software was used to modify the sequence (*AIP* variants), the oligomeric state or to mark concrete residues within the 3D structure of a protein.

- For Caspase-3: we used the model based on the PDB 5i9b (obtained by X-ray diffraction, 1.8Å) of the human Caspase-3 (Uniprot # P42574) that corresponds to a homodimer (residues 29 to 277) in SWISS-MODEL. In this case, UCSF Chimera was used to modify the surface texture and for identification of residues corresponding to the active site.
- For RET: we used a model whose sequence covers residues 705-1025 generated from the PDB 4cki (residues 705-1013, crystallographic structure obtained by X-ray diffraction, 2.12Å) and the human RET sequence (Uniprot # P07949) in SWISS-MODEL. As this RET model was a homodimer, using UCSF Chimera a unit was removed from the complex, giving rise to the 3D model of the monomeric RET human protein of 705-1025 aminoacids.
- For AIP: we used a model sequence covering residues 8-329 generated from the PDB 1qz2 (obtained by X-ray diffraction, 3Å) and the AIP sequence (Uniprot # O00170). This model generated in SWISS-MODEL was a homotrimer. Using UCSF Chimera one or two units were eliminated from the trimer, giving rise to the 3D model of the dimeric or monomeric human AIP protein of 8-329 amino acids.
- For PKCδ: we used human PKCδ sequence (Uniprot # Q05655) and since there were no available PDBs of PKCδ, by homology as recommended we used the PDB of PKCβ 3pfq (residues 230-672, obtained by X-ray diffraction, 4Å, homology 58.10%). To this, the complete human PKCδ sequence was incorporated in SWISS-MODEL; the model generated was a monomer. The UCSF Chimera program was used to modify the surface texture and to identify specific residues of the sequence.

Retrovirus production and functional validation

Human myc-wt*AIP* or variant constructs cloned in pCDNA3.1 were recloned in in the bicistronic retroviral vector MIGR1, contained a multicloning site followed by an IRES and a second coding sequence corresponding to EGFP (Addgene) (Suppl. Methods Table 1) using pre-designed primers for the pCDNA3.1 multicloning site forward 5'GCATGACTCGAGTTCACCATGGAACAAAAGTTGATTTCTGAAGAA 3' and reverse 5' TAAGCAGTTAACTGAATGGGAGAAGATCCCCCGGAA 3' (Eurofins Genomics, Thermo Fisher Scientific). These primers contain the restriction enzymes XhoI (5' end) and HpaI (3' end) for myc-AIP direct cloning into the multicloning site of MIGR1.

100 ng of a pcDNA3.1-myc-AIP construct were mixed with 0.1 µL Platinum™ Taq DNA Polymerase High Fidelity (11304011, Thermo Scientific), 2.5 µL 10X TaqmanPCR Buffer, 1 µL of 50 mM MgCl2, 0.5 µL dNTPs 10 mM, 0.5 µL of 10 µM Forward primer and 0.5 µL of 10 µM Reverse primer and distilled water to a final volume of 24 µL. The PCR was performed in T100™ Thermal Cycler (1861096, Biorad): 1cycle 94ºC for 2 minutes and 40 cycles of 94ºC for 15 seconds, 59ºC for 30 seconds, 68ºC for 1 minute and a final step of 68ºC for 5 minutes.

Amplicons and MIGR1 vector were cut with XhoI and HpaI (R0146S, R0105S, New England Biolabs) followed by vector dephosphorylation (0.5 IU CIP, M0290S, New England Biolabs). Purification steps for amplicons directly after PCR and MIGR1 restricted vector from agarose gel were performed with GeneJET (K0832, Thermo Fisher Scientific). Ligations were performed using Quick Ligation (M2200S, New England Biolabs).

After transformation and replication on Subcloning Efficiency™ DH5α™ competent cells (18265-017, Invitrogen), plasmids were purified using NucleoSpin® Plasmid (740588.50, Macherey-Nagel) for minipreps, and "EndoFree TM" (12362, Qiagen) for maxipreps.

Retroviral final constructs (myc-AIP-IRES-EGFP-MIGR1, so called myc-AIP-MIGR1) were submitted to diagnostic restriction with enzymes, Sanger sequenced, and transfected in GH4C1 cells for *in vitro* EGFP expression and cell lysates to perform western blot for GFP and myc-AIP expression.

The myc-AIP-MIGR1 constructs were used to obtain retroviruses. Production of the retroviral particles was carried out by transfection in HEK293FT ($2x10^6$ cells) using 20 µg of MIGR1 or MIGR1-myc-AIP, 15 µg of pHelper virus and 6 µg of pVSV-G using the 2X Hanks saline solution (HBSS)-$CaCl_2$ method. Between 48 -72 hours post transfection medium was collected every day. Retroviral particles were concentrated from the medium by ultracentrifugation.

A quantitative viral titer was calculated by qPCR. For the calibration curve we used the wt-AIP-MIGR1 retroviral plasmid with serial dilution. For the extraction of retroviral DNA, we used the PureLink™ Viral RNA/DNA Mini Kit (12280-050, Invitrogen). 1 µL of DNA samples were mix with 9 µL of 10 µM Forward primer 5'ATAGACTGCGTCGCCCGG3' (0.4 µL/sample), 10 µM Reverse primer 5'TCCCAAGGATCAGCGAGTC3' (0.4 µL/sample) for the LTR region were used, together with Brilliant III Ultra-Fast SYBR MasterMix (Agilent Technologies) 2X (5 µL/sample), and distilled water (3.2 µL/sample). The qPCR protocol was step 1 (95ºC 10 minutes); step 2 (95ºC for 15 seconds and 60ºC for 30 seconds) x40 times and a final step (72ºC for 30 seconds). Data were analysed and copy number of the samples extrapolated from the standard curve.

On the other hand, a functional viral titer (TU/mL) was obtained by transduction of serial dilutions of viral particles (25 µL) in cells (HEK293FT and GH4C1) seeded in 24-well plates and

counting the number of positive colonies (expressing EGFP) x dilution factor x40 (to achieve 1 mL). This gave us the MOIs of each retrovirus.

Finally, viral particles were evaluated by transduction of wtAIP-MIGR1, V49M-AIP-MIGR1 and R304Q-AIP-MIGR1 in GH4C1 and rat pituitary primary cell cultures (without serum and antibiotics) using 5 MOI of retroviral particles and 8.9 µg/mL Polybrene for 6 hours after which complete medium with serum and antibiotics were added and incubated for 48 hours. For this, 50.000 GH4C1 cells/well or one dispersed pituitary/well were seeded in a 24-well plate for 48 hours before transduction. This was followed by RET transfection with Turbofect. After deprivation, apoptosis was evaluated at 48 hours and lysates were tested for expression of RET, Pit-1, p53, GFP, AIP and myc-tag by western blot.

In vivo **rat model to functionally evaluate missense AIP variants in pituitary**

Procedures were carried out under the Procedures Act nº 15003/14/005 PI_Prof. Clara Álvarez Villamarín granted by Galicia Regional Government. Experiments were approved by the Bioethical Committee of the University of Santiago de Compostela following the current regulations: 2007/526/CE, De296/2008, Directive 2010/53/EU, RD 53/2013 and ECC/566/2015.

The experiment compared injection of seven retrovirus: MIGR1 (control), wtAIP, and five of our pathogenic AIP variants (R16H, V49M, C238Y, R304Q and R325Q). Each animal was considered an experimental unit being followed from injection after birth till week 16. Sample size was based on total length (cm) since it was a known physiological parameter for adult rats with a median of 47cm and a SD of 5 cm for males. Expecting an increase in length >7%, with an alpha p value of <0.05 and 90% power the minimal sample size of animals per condition was n=15 (Prof. Weyne Lamorte Calculator, Harvard).

Retrovirus infect actively proliferating cells [37]. We had experience with stereotaxic injection of retrovirus in a model of induced hyperplasia in adult rat pituitary [20]. However, this model had problems on its own derived from the absence of mouse/rat stereotaxic maps including the pituitary, the need to wait until adulthood not being able to inject during the infancy and pubertal growth, and the possibility to damage key hypothalamic nuclei regulating the adenohypophysis, such as the arcuate or periventricular nuclei containing hypothalamic regulatory factors GHRH and Somatostatin. A physiological model for proliferation in the adenohypophysis is just after birth, where either stem cells, progenitors or differentiated cells are actively proliferating [38, 39].

The usual surgical pathway for rodent hypophysectomy is through the ear, with a needle inserted at 45º following the ear canal [40]. In a newborn rodent at P1, the pituitary is located in very close proximity to the external ear separated only by a thin tissue layer. We tested our system in P1 rat pups injecting a small volume of saline with ink. These animals were alive and healthy at long term, with the ink localized at the pituitary after the injection.

Female *Sprague Dawley* rats at *terminus* were stabulated under normal conditions (12h light / 12h dark) at 22 ± 2 ° C with food and water *ad libitum*. We used the minimal number of female rats to achieve a minimal sample size of 10 individuals per gender per construct retrovirus. 100 µL of 10^8 TU of a retrovirus (7 different retroviruses: empty vector (MIGR1) and 6 AIP retroviruses: myc-wtAIP-MIGR1, myc-R16HAIP-MIGR1, myc-V49MAIP-MIGR1, myc-C238YAIP-MIGR1, myc-R304QAIP-MIGR1, myc-R325QAIP-MIGR1) were mixed with 10^{-8} M of Ghrelin (stock 10^{-6} M, G8903), 25 ng/mL of GDNF (stock 10 µg/mL. PF039, Calbiochem) and 10^{-7} M of GHRH (stock 10^{-8} M, G6771). The P1 pup was anesthetized by hypothermia for 5 minutes. Using a Hamilton syringe of 10 µL with parrot-beak tip (701N 1011869, Hamilton) at 90º and 6 mm of depth, 6 µL of the mixture were injected into the pituitary gland through the right ear canal. After 30 seconds, the

needle was gently removed. It is important that no blood was observed after removing the needle. Quickly, the pup was placed on a warm blanket to recover body temperature and returned with their progenitor until after weaning. Litters were adjusted to a similar number per mother rat (approximately 10 pups/litter). At day 20, rats were identified with a code and divided according to their sex. Animals from different litters injected with different retrovirus were randomized and stabulated under 12-hour light and dark cycles, 22 ± 2ºC and with water and food *ad libitum*. Rat number was adjusted to 6-8 per cage.

Body weight was measured once per week in a balance (Dune DCT2000, Adam Equipment). The experiment end at week 16 (112 days), where different measurements, such as the determination of body composition (Whole body Composition Analyzer; Echo MRI™) or phenotypic measurements, and blood extraction were carried out before sacrificing the rats. Serum was obtained from blood and $_f$IGF1 measured with ELISA Free Rat/Mouse IGF-1 (AL-136, Anshlabs).

Phenotypic measurements were selected from the literature as bona fide parameters altered by GH/ IGF-1 excess in rodents such as increase in body weight [41], in craniofacial size [42] or in femur, tibia or tail length [43, 44]. In addition, human patients with excess GH present growth of soft tissues including cartilage and thus have bigger nose, ears, as well as the acral extremities (hands and feet).

Physical measurements made in anaesthetized (ketamine/xylazine) rats were:

- length of the body, tail and total length: rats were held vertically by the upper incisors and lengths measured on graph paper;
- abdominal perimeter: measured with tape;
- length of the tibia and femur: the sole of the foot is supported on a flat surface, allowing the joint to bend and facilitating its measurement with a calliper;
- length of the hand, foot, ear and jaw, head length and width and upper and lower incisors length: measured with a calliper.

Deeply anaesthetized rats were perfused only with 100-200 μL of 5% glucosaline solution to remove blood from the tissues, and fresh organs dissected and weighed (pituitary, heart, liver, muscle and all white body fat). Half of the pituitary glands were frozen to be lysated for protein extracts and western blot (stored at -80ºC), and the other half were fixed by immersion in 10% neutral buffered formalin for 24 hours.

Some younger 1 to 4 weeks old rats were fully perfused with glucosaline and 10% of formalin to perform colocalization by immunofluorescence (see below).

Adenohypophysis immunocytochemistry and Immunofluorescence. Area calculation.

Deeply anesthetized animals were perfused with 5% glucosaline and pituitaries immersed in 10% neutral buffered formalin. After 24 hours fixed rat pituitaries were incubated in 70% ethanol for another 24 hours. Samples were further processed in alcohol gradients and xylol using the Leica TP1020 Semi-Enclosed Benchtop Tissue Processor system and finally included in paraffin blocks. To prevent technical biases, 3 μm paraffin sections from three pituitaries of wtAIP injected rats were

mounted together with another three from R304Q and three from C238Y (in total 9 sections per slide) (and sometimes another MIGR1 section for negative control). In this way, all sections received the same antibody and rinses during the immunohistochemistry. Reticulin staining was performed with commercial solution (Dako) followed by Fast Red counterstaining.

For immunohistochemistry (IHC) antigen retrieval was performed in a PT Link (Dako) with High/Low pH buffer (Dako). Reactives and secondary antibody from EnVision (Dako) system were used. Quantification was performed using Fiji-Image J 1.51.

For immunofluorescence retrieval was performed in the PT Link with High pH buffer. Triple immunofluorescences were performed using primary and secondary antibodies in PBS and mounted with Fluoro-Gel (17985-10, Electron microscopy Sciences) including 2mg/mL of DAPI (D9542, Sigma). Colocalization was studied with a Leica TC SP5-AOBS confocal microscope equipped with a white laser (470-670nm) using a 63X oil objective (NA=1.5) and the LAS AF software (Leica Application Suite Advanced Fluorescence).

Antibodies' conditions and dilutions are in Supplementary Information.

To calculate the adenohypophysis area, 10 sections of each pituitary were photographed with a 2x objective (Olympus) calibrated for a linear scale. To prevent bias due to orientation of the paraffin block, 5 contiguous sections were added to other 5 sections discarding the 5 sections in the middle. Adenohypophysis areas were calculated with Image J based on the scale given by the microphotograph.

Whole pituitary clarification with X-Clarity. Light-sheet microscopy

7 days after injection in P1 rats were anesthetized with ketamine-xylazine and perfused with glucosaline followed by 10% neutral formalin. The pituitary gland was dissected and post-fixed for 24 hours in 10% formalin.

Pituitary clearing was performed with X-CLARITY (Logos Biosystems) following manufacturer instructions and its reagents (Hydrogel C13103, Polymerization Initiator C13104, Electrophoretic Tissue Clearing Solution C13001, Mounting Solution C13101). Images were taken using Light-Sheet Ultramicroscope II (LaVision Bio Tech) in a configuration of an 2X objective (Olympus MVPLAPO) with single-zoom optical path (Olympus MVX-10 Zoom) and with a CMOS camera (Andor Neo NEOCM). Pituitary samples were recorded at 40% of laser power, 0.035 numerical aperture, 60 blade width and a maximum intensity of 50.000. Subsequent analysis of the images was carried out with Imaris 9.2 software (Bitplane). Videos were made with and without pituitary marked volume.

Pituitary-specific AIP knockout mouse model

Animal studies were performed following the NC3Rs' ARRIVE guidelines. Procedures were carried out under the Animal Licence Permission No PPL 70/7665, supported by QMUL's Ethics Committee. The two lines are derived from C57BL/6J (www.jax.org). Pituitaries (n=3 per group) from 15-week-old pituitary-specific *Aip*-knockout mice $Aip^{Flox/Flox}$;$Hesx1^{Cre/+}$ who develop GH-secreting pituitary tumours or $Aip^{Flox/Flox}$;$Hesx1^{+/+}$ littermates [45, 46] were frozen after dissection or fixed in formalin as above.

Immunohistochemistry of patients somatotrophinomas

Ethical approval of the study was obtained from Cambridgeshire 1 Research Ethics Committee, Cambridge, UK (06/Q0104/133). Patients gave written informed consent. 3 μm sections were obtained from paraffin blocks. Antigen retrieval was performed in PTlink with High pH buffer (Dako), followed by all steps and commercial solutions of the EnVision protocol (Dako). Primary antibody incubations were overnight at 4º. Dilutions are indicated in Supp Information. Quantification was performed by independent double observers in the whole set.

Data collection and statistical analysis

Sample size calculation was obtained the calculator of Prof. Weyne W LaMorte (Boston University, https://sphweb.bumc.bu.edu/otlt/MPH-Modules/Excel/Excel6.html) using previous known data. For in vitro experiments median and SD apoptosis data for RET transfection, GDNF survival and the hypothesis of an apoptosis reduction of at least 35% with 95% power at p value 0.01 the minimal number was 3 independent experiments. For *in vivo* experiments we based our calculations in our previous weight curves or data from literature because initially the standard deviation of variables of measured variables of rats during growth was unknown; again for a change of at least 35% with 90% power and p value 0.05 minimal sample size was 12 animals. We tried to adhere to this number except for unforeseen events like unequal gender-distribution within injected litters.

In vitro experiments (microscopy counting, western blots, caspase and luciferase activity) were performed at least in 3 independent experiments with 4 replicates for each condition in every experiment. Data are represented as media±SEM.

In vivo injections were performed in three independent groups of animals being the 6 AIP variants and MIGR1 control represented in every group. The minimum sample size/gender for any *AIP* variant was n=10. Data are represented in boxplots, where each box spans the interquartile range Q1-Q3 (25%-75%), the line in each box represents the median and the whiskers represent the maximum and the minimum value of each group data.

Statistical analysis was performed using GraphPad Prism 8.0.1 (San Diego, USA), SPSS Statistics 20 (IBM, USA) and Corel Draw Graphic Suite 2017.

To compare between three or more groups, we performed One-way, Two-ways or Three-ways ANOVA for samples with normal distribution (parametric test) or Kruskal-Wallis test for non-normal distribution (non-parametric test). Dunnett's (compare all the groups with one specific group), Tukey's or Holm-Sidak's (compare all the groups between them) corrections test were applied for One-way ANOVA, and Dunn's or Holm-Sidak's correction test (compare all the groups between them) were applied for Kruskal-Wallis test. Sidak's or Tukey's correction test were applied for Two-ways or Three-ways ANOVA.

Correlations between groups were assessed with Spearman's Rho (rs) coefficient and with Chi^2 test.

In all the analysis performed, the difference between groups had statistical significance when the probability achieved $p \leq 0.05$ (*$p \leq 0.05$. **$p \leq 0.01$ and ***$p \leq 0.001$).

Suppl. Methods Table1. Vectors, plasmids and siRNA

Vectors, plasmids and siRNA	Description	Origin
pcDNA3.1	Empty vector. Size: 5248bp	V790-20, Invitrogen
pcDNA3.1-myc-wtAIP	Full length sequence of human AIP. Tag: N-terminal Myc-tag. Size: 6436 bp.	M. Korbonits
pcDNA3.1-myc-R16HAIP		
pcDNA3.1-myc-V49MAIP		
pcDNA3.1-myc-I257VAIP		
pcDNA3.1-myc-C238YAIP		
pcDNA3.1-myc-R271WAIP		
pcDNA3.1-myc-R304QAIP		
pcDNA3.1-myc-R304XAIP		
pcDNA3.1-myc-R325QAIP		
pcDNA3-RET$_S$	Short isoform of human RET	M.G.Borrello [47]
pcDNA3-RET$_L$	Long isoform of human RET	M.G.Borrello [47]
pcDNA3-ICRETD707-1017	Intracellular fragment (707-1017) of RET	P. Mehlen [48]
PKC delta WT	Mouse PKC delta cloned in a pHACE vector. Tag: HA. Size: 7500 bp.	B. Weinstein (Addgene plasmid # 16386) [49]
PKC delta DN	Mouse PKC delta with a kinase inactive mutation (K376R) cloned in a pHACE vector. Tag: HA. Size: 7500 bp.	B. Weinstein (Addgene plasmid # 16389)[49]

p-378 Pit1-luc	-378 sequence of the Pit1 promoter cloned in a pGL3basic vector that expressed luciferase.<br><br>Size: 5196 bp.	A.García [22]
pSV-β-Galactosidase	β-Galactosidase expression plasmid.<br><br>Size: 6820 bp.	E1081, Promega
pmaxGFP® Vector	GFP expression plasmid.<br><br>Size: 3486 bp	Kit VCA-1005, Lonza
pHelper virus	Contains Gag and Pol genes.	Canibano [20]
pCMV-VSV-G	Contains viral envelope genes. Size: 6383 bp.	B. Weinberg (Addgene plasmid # 8454) [50]
MIGR1	IRES-EGFP retroviral vector.<br><br>Size: 6441pb.	W. Pear (Addgene plasmid # 27490) [51]
myc-wtAIP-MIGR1	Human wt-AIP and AIP mutants (obtained from pCDNA3.1-myc-AIP) cloned in MIGR1 (IRES-EGFP) retroviral vector. Tag: N-terminal Myc-tag.<br><br>Size:7477 bp.	In house
myc-R16HAIP-MIGR1		
myc-V49MAIP MIGR1		
myc-I257VAIP-MIGR1		
myc-C238YAIP-MIGR1-		
myc-R271WAIP-MIGR1		
myc-R304QAIP-MIGR1		
myc-R325QAIP-MIGR1		
MISSION®siRNA Universal Negative Control	Add 100 μL of miliQ water.	SIC001, Sigma

	0.8 µg per transfection	
Rat AIP-MISSION®<br><br>Pre-designed siRNA-2 OD (PDSIRNA2D)<br><br>• siRNA1: SASI_Rn01_00111569<br><br>• siRNA2: SASI_Rn01_00111571	Add 100 µL of miliQ water. Concentration of use: combination of 0.4 µg of each siRNA (Total=0.8 µg).	PDSIRNA2D, Sigma

Suppl. Methods Table 2. Experimental conditions for matched transfections and protein extractions

Plasmids	Transfection method	Protein extraction buffer: Figures
0.25 μg pcDNA3.1-myc-AIP<br><br>or 0.8 μg Aip siRNA or siRNA non target<br><br>3.5 μg pcDNA3-RET$_S$ or pcDNA3-RET$_L$ or 3 μg IC-RET or empty<br><br>vector (pcDNA3.1) or 0.5 μg PKCδwt or PKCδDN	Amaxa	SDS-Lysis Buffer:<br><br>FIG1(B/C/D); FIG2 (A/B/C/E/F/G/J); SUP2 (A/B/C); FIG3 (A/B); SUP3 (A/B); FIG4 (D)
		Lysis Buffer:<br><br>FIG3 (F); FIG5 (A)
		Subcellular fractionation:<br><br>SUP4; FIG4 (A/B/C)
1 μg pcDNA3.1-myc-AIP or 1 μg Aip siRNA	Turbofect	SDS-Lysis Buffer:<br><br>FIG2 (H/I)
0.5 or 1 μg myc-AIP-MIGR1 retroviral plasmids		Lysis Buffer:<br><br>SUP6 (B)
3.5 μg pcDNA3-RET$_S$		Lysis Buffer (1 hour) and SDS-Lysis Buffer (48 hours):<br><br>SUP6 (C)

Suppl. Methods Table 3. Antibodies for Western blot

Primary antibodies	Reference	Epitope	Concentration	Solution	Incubation	Origin
**XAP2 (35-2)**	sc-59730, Sta Cruz Biotech (SCBT)	Full length	1:1000 (1:500)	I-Block™ (*)	2hours (h) RT	M
**ARA9 (35-2)**	NB100-127, Novus Biologicals	Total	1:1000	I-Block™	2h RT	M
**c-Myc (9E10)**	sc-40, SCBT	408-439aa (C-terminal)	1:750	I-Block™	2h RT	M
**Ret (C31B4)**	3223, Cell Signalling Tech (CST)	1017-1063aa	1:1000 + 0,1% Tween20® (1:500)	I-Block™	2h RT or o/n 4ºC plus 1h RT	R
**Ret (C-3)**	sc-365943, SCBT	31-330aa (N-terminal)	1: 500 (1:250)	I-Block™	o/n 4ºC plus 1h RT	M
**Ret (C19)**	sc-167, SCBT	C-terminal (9aa isoform)	1:500	I-Block™	2h RT or o/n 4ºC plus 1h RT	R
**Ret (C20)**	sc-1290, SCBT	C-terminal (51aa isoform)	1:500	I-Block™	2h RT or o/n 4ºC plus 1h RT	G
**Ret (8D10C9)**	sc-101422, SCBT	896-1063aa	1:1500	I-Block™	2h RT	M
**Ret (HPA008356)**	HPA008356, Sigma	C-terminal (9aa and 51aa isoforms)	WB:1:1000 IHQ: 1:100	WB:I-Block™ IHQ:Envision	2h RT or o/n 4ºC plus 1h RT (WB or IHQ)	R
**Caspase-3 (H277)**	sc-7148, SCBT	1-277aa	1:1000	I-Block™	2h RT or o/n 4ºC plus 1h RT	R

**Cleaved Caspase-3 (Asp175) (5AE)**	9664, CST	Residues near Asp175<br><br>(N-terminal)	1: 500	I-Block™	o/n 4ºC plus 1h RT	R
**Pit-1(X-7)**	sc-442, SCBT	Full length	1:1000	I-Block™	o/n 4ºC plus 1h RT	R
**Pit-1 (D-7)**	sc-393943, SCBT	5-32aa<br><br>(N-terminal)	1:1000<br><br>(1:500)	I-Block™	2h RT or o/n 4ºC plus 1h RT	m-IgGκ
**CDKN2A/<br><br>p19ARF**	ab80, Abcam	50-150aa	1:500	I-Block™	o/n 4ºC plus 1h RT	R
**p19 ARF<br><br>(5-C3-1)**	sc-32748, SCBT	62-75aa	1:500	I-Block™	o/n 4ºC plus 1h RT	m-IgGκ
**p14 ARF (C-18)**	sc-8613, SCBT	C-terminal	1:500	I-Block™	o/n 4ºC plus 1h RT	G
**p53 (Ab-1)**	OP03, Calbiochem	376-378aa	1:500	BSA (**)	o/n 4ºC plus 1h RT	M
**p53 (A-1)**	sc-393031, SCBT	353-391aa	1:500	I-Block™	o/n 4ºC plus 1h RT	M
**GDNF (D-20)**	sc-328, SCBT	C-terminal	1:500<br>IHQ:1:100	I-Block™<br>IHQ:Envision	2h RT or o/n 4ºC plus 1h RT(WB or IHQ)	R
**GFRA1 (C20)**	sc-6157,<br><br>SCBT	C-terminal	1:500<br>IHQ: 1:100	I-Block™<br>IHQ: Envision	o/n 4ºC plus 1h RT	G
**PKCδ (C-17)**	sc-213, SCBT	C-terminal	1:1000<br><br>(1:1000)	I-Block™	2h RT	R
**phospho-PKCδ/θ<br><br>(Ser 643/676)**	9376, CST	p-Ser 643/676<br><br>(C-terminal)	1:1000+0,1% Tween20®<br><br>(1:500)	I-Block™	2h RT or o/n 4ºC plus 1h RT	R

**phospho-SAPK/JNK (Thr183/Tyr185)**	9251, CST	p-Thr183 /Tyr185 de p46 y p54	1:750+0,1% Tween20® (1:500)	BSA	o/n 4ºC plus 1h RT	R
**phospho-CREB (Ser133)(1B6)**	9196, CST	p-Ser133	1:500+0,1% Tween20®	I-Block™	o/n 4ºC plus 1h RT	M
**GH**	AFP11981, NIHH	Full length	1:1000	I-Block™	o/n 4ºC plus 1h RT	R
**GFP (EPR14104)**	ab183734, Abcam	Full length	1:1000 (1:500)	I-Block™	o/n 4ºC plus 1h RT	R
**p-AKt (Ser473)**	9271S, CST	Synthetic phosphopeptide corresponding to residues surrounding Ser473 of mouse Akt. Detects endogenous levels of Akt1 only when phosphorylated at Ser473. This antibody also recognizes Akt2 and Akt3 when phosphorylated at the corresponding residues. It does not recognize Akt phosphorylated at other sites, nor does it recognize phosphorylated forms of related kinases such as PKC or p70 S6 kinase.	1:1000	I-Block	o/n 4ºC plus 1h RT	R
**Akt**	9272, CST	Akt1/2/3	1:1000	I-Block™	o/n 4ºC plus 1h RT	R
**α-Tubulin**	T5168, Sigma	C-terminal	1:2500	I-Block™	1h RT	M
**ß-Actin (AC-74)**	A5316, Sigma	Full length	1:1000	I-Block™	1h RT	M
**Cleaved PARP (Asp214) (D64E10)**	5625, CST	Residues near Asp214	(1:500)	BSA	o/n 4ºC plus 1h RT	R
**E-Cadherin (NCH-38)**	GA05961-2, Agilent DAKO	Full length	1:1000	I-Block™	o/n 4ºC plus 1h RT	m-IgGκ
**SUCLA-2**	ab97868, Abcam	138-386aa	1:500	I-Block™	o/n 4ºC plus 1h RT	R

**BiP**	3183, CST	Full length	1:000	I-Block™	8h 4ºC and 1h RT	R
**GM130**	2296R, CST	Full length	1:1000	I-Block™	o/n 4ºC plus 1h RT	R
**EEA1**	Ab2900, Abcam	C-terminal	1:500	I-Block™	o/n 4ºC plus 1h RT	R
**Histone H3**	9715, CST	Full length	1:1000	I-Block™	o/n 4ºC plus 1h RT	R
**Secondary Antibodies**		**Reference**	**Concentration**	**Solution**	**Incubation**	
**Polyclonal Goat Anti-Rabbit IgG-HRP**		A0545, Sigma	1:10000	I-Block™	1hour RT	
**Polyclonal Rabbit Anti-Mouse Inmunoglobulins-HRP**		P0260, Dako	1:5000	I-Block™	1hour RT	
**Mouse-IgGκ BP-HRP**		sc-516102, SCBT	1:500	I-Block™	1hour RT	
**Donkey anti-Goat IgG-HRP**		sc-2020, SCBT	1:5000	I-Block™	1hour RT	

M=mouse/R=rabbit/G=goat/D=donkey. (*) 2 g/L *I-Block™ (*T2015, Thermo Fisher Scientific) *in 1X TBS*. (**) 5% BSA (A7906, Sigma) in 1X TBS

Suppl. Methods Table 4. Primers for qRT-PCR

Gene	Sequence	Amplicon	Hybridization temperature
**Tbp**	Fw: 5'-CTTCGTGCCAGAAATGCTGAA-3'	79bp	60ºC
	Rv: 5'-CAGTTGTTCGTGGCTCTCTTATTCTC-3'		
	Pb: 5'-AATCCCAAGCGGTTTGCTGCAGTCA-3'		
**Aip**	Fw: 5'-GGAGAGTTGCCGGAATTTCA-3'	74bp	60ºC
	Rv: 5'-CCCTCCGGGTCACTATGCA-3'		
	Pb: 5'-ATGGCACTAAGGCCACCTTCCAC TTCC-3'		
**Ret**	Fw: 5'-GGCTGTCCCGAGATGTTTATGA-3'	84bp	60ºC
	Rv: 5'-ACTCGATTGCCATCCATTTGAC-3'		
	Pb:5'-TGTGAAGAAAAGCAAGGGCCGGATTC-3'		
**RET human**	Hs01120030_m1, Applied Biosystems	83bp	60ºC
**Gdnf**	Fw: 5'-CCCGAAGATTATCCTGACCA-3'	242bp	60ºC
	Rv: 5'-TAGCCCAAACCCAAGTCAGT-3'		
**Gfrα1**	Fw: 5'-GGCGGCACCATGTTCCTA-3'	116bp	60ºC
	Rv: 5'-CACTGATCACTGGCTTTCACACA-3'		
	Pb: 5'-CCTGGATTTGCTGATGTCGGCCG-3'		
**Gh**	Fw: 5'-TGGCTGCTGACACCTACAAAGAG-3'	78bp	60ºC

	Rv: 5'-CCTGGGCATTCTGAATGGAA-3'		
	Pb: 5'-TCGAGCGTGCCTACATTCCCGAGG-3'		
**Ghrhr**	Fw: 5'-CACAAGGCGGCTTACACACA-3'	80bp	60ºC
	Rv: 5'-AATTCCAAACAGCGGGATAAGG-3'		
	Pb: 5'-TCAGTACTGGCGGCTTTCCAAATC AACACT-3'		
**Prl**	Fw: 5'-CCCTAGCTACTCCTGAAGACAAGGA-3'	85bp	60ºC
	Rv: 5'-GAGTGCACCAAACTGAGGATCA-3'		
	Pb: 5'-AAGCCCAGAAAGTCCCTCCGGAA GTTC-3'		
**Sf1**	Rn00584298_m1, Applied Biosystems:	65bp	60ºC
**Pomc**	Fw: 5'-CGTCCTCAGAGAGCTGCCTTT	98bp	60ºC
	Rv: 5'-TGTAGCAGAATCTCGGCATCTTC		
	Pb: 5'-CGCGACAGAGCCTCAGCCACC		
**Fshβ**	Fw: 5'-GATAGCCAACTGCACAGGACATAG	68bp	60ºC
	Rv: 5'-ATGCAAAGCTGGATCGACTTC		
**Prop1**	Fw: 5'-GCCTTTGGGAGGAACCAGTATC	140bp	60ºC
	Rv: 5'-TGGAGTAGTGACCGCTCTTGCT		
	Pb: 5'-TCAGCGAAGCCAGAATCCAGGTCT		
**Tshβ**	Fw: 5'-TCTGCGCTGGGTATTGTATGAC-3'	79bp	60ºC

	Rv: 5’-CAGACATCCTGAGAGAGTGCGTAC-3’		
	Pb: 5’-CGGGATATCAATGGCAAACTGTTTC TTCCC-3’		
**Sstr2**	FW:5’-TGGAAAAGCAAGATGTCACG-3’	168bp	60ºC
	RV: 5’-CGTTGAGGTCAAAGGGAGAA-3’		
**Sstr5**	FW:5’- CTGTCCTGCACAGAGACACG-3’	136bp	60ºC
	RV: 5’-GCCCACCAGTGACCAGTTA-3’		
**Pit-1**	Fw: 5’-GCCTCTGAGAATGCACCACAAT-3’	130bp	60ºC
	Rv: 5’-GGTGGATGGCTGGTTTCCA-3’		
	Pb: 5’-CCACCAACGTGATGTCCACAGCGA-3’		
**p19Arf (Cdkn2a)**	Fw: 5’-CAGGCATAACTTCTGCTCAAGCA-3’	91bp	60ºC
	Rv: 5’-AAAGGAGGGCTGAGGCCTAA-3’		
	Pb: 5’-CCAGGTGCCTAGGACT-3’		

Suppl. Methods Table 5. Antibodies for PLA Duolink

	Conditions	1st Antibodies	Concentration	Antibody diluent (final volume)
Samples	PLA α-mouse-mycAIP+α-rabbit-RET	c-Myc (9E10)	1:400	50 µL
		RET (C19)	1:200	
	PLA α-mouse-mycAIP+α-rabbit-Caspase-3	c-Myc (9E10)	1:400	50 µL
		Caspase-3 (H277)	1:300	
	PLA α-mouse-mycAIP+α-rabbit-PKCδ	c-Myc (9E10)	1:400	50 µL
		PKCδ (C-17)	1:400	
Negative controls	PLA α-mouse mycAIP+α-rabbit-GFP	c-Myc (9E10)	1:400	50 µL
		GFP (EPR14104)	1:1000	
	PLA α-mouse-mycAIP	c-Myc (9E10)	1:400	50 µL
		---	---	
	PLA without primary antibodies	---	---	50 µL
		---	---	

Suppl. Methods Table 6. Conditions for tissue IHQ and IF

Immunohistochemistry						
ICC	PT Link Retrieval	Antibody	Origin	Concentration	Incubation	Reference
Primary	Dako pH High	GFP	Rabbit	1:4000	ON 4ºC	ab183734, Abcam
	TE	Ret(8D10C9)	Mouse	1:100	30 minutes RT	sc-101422, SCBT
	Dako pH Low	phospho-PKCδ/θ (Ser643/676)	Rabbit	1:100	ON 4ºC	9376, CST
	CB	Cleaved Caspase-3 (Asp175) (5AE)	Rabbit	1:200	ON 4ºC	9664, CST
	Dako pH High	RET (HPA008356)	Rabbit	1:100	ON 4ºC	HPA008356, Sigma
	Dako pH High	GFRA1(C-20)	Mouse	1:100	ON 4ºC	sc-271546, SCBT
	Dako pH High	GDNF(D-20)	Rabbit	1:100	ON 4ºC	sc-328, SCBT
Secondary	----	EnVision©+ Dual Link System-HRP	----	Ready to use	30 minutes RT	EnVision, DAKO
**Immunofluorescence**						
IF	PT Link Retrieval	Antibody	Origin	Concentration	Incubation	Reference
Primary	TE	GFP	Chicken	1:600	ON 4ºC	1020, Aves labs

	TE	GFP	Rabbit	1:1000	ON 4ºC	ab183734, Abcam
	TE	Pit1 (D7)	Mouse	1:300	ON 4ºC	sc393943, SCBT
	TE	Gh	Rabbit	1:1000	ON 4ºC	AFPC11981A, NIDDK
Secondary		Anti-Mouse IgG Alexa Fluor 647	Donkey	1:1000	1hour RT	714-606-151 Jackson I.
		Anti-Chicken IgG Alexa Fluor 488	Donkey	1:1000	1hour RT	703-546-155, Jackson I.
		Anti-Rabbit IgG Cy3	Donkey	1:1000	1hour RT	711-166-152, Jackson I.

References

1. Zerbino, D. R. et al. Ensembl 2018. *Nucleic Acids Res*. **46,** D754-D761 (2018).
2. Kopanos, C. et al. VarSome: the human genomic variant search engine. *Bioinformatics* **35,** 1978-1980 (2019).
3. Landrum, M. J. et al. ClinVar: improving access to variant interpretations and supporting evidence. *Nucleic Acids Res*. **46,** D1062-D1067 (2018).
4. Sherry, S. T., Ward, M. H., Kholodov, M., Baker, J., Phan, L., Smigielski, E. M. & Sirotkin, K. dbSNP: the NCBI database of genetic variation. *Nucleic Acids Res*. **29,** 308-11 (2001).
5. McLaren, W. et al. The Ensembl Variant Effect Predictor. *Genome Biol*. **17,** 122. (2016) .
6. Rentzsch, P., Witten, D., Cooper, G. M., Shendure, J. & Kircher, M. CADD: predicting the deleteriousness of variants throughout the human genome. *Nucleic Acids Res*. **4**7, D886-D894 (2019).
7. Ioannidis, N.M. et al. REVEL: An Ensemble Method for Predicting the Pathogenicity of Rare Missense Variants. *Am J Hum Genet*. **99,** 877-885 (2016).
8. Dong, C. et al. Comparison and integration of deleteriousness prediction methods for nonsynonymous SNVs in whole exome sequencing studies. *Hum Mol Genet*. **24,** 2125-2137 (2015).
9. Dehouck, Y., Kwasigroch, J.M., Gilis, D. & Rooman, M. PoPMuSiC 2.1: a web server for the estimation of protein stability changes upon mutation and sequence optimality. *BMC Bioinformatics*. **12,** 151 (2011) .
10. Lundin, E., Tang, P. C., Guy, L., Näsvall, J. & Andersson, D. I. Experimental Determination and Prediction of the Fitness Effects of Random Point Mutations in the Biosynthetic Enzyme HisA. *Mol Biol Evol*. **35,** 704-718 (2018).
11. Ancien, F., Pucci, F., Godfroid, M. & Rooman, M. Prediction and interpretation of deleterious coding variants in terms of protein structural stability. *Sci Rep*. **8,** 4480. (2018).
12. Vaser, R., Adusumalli, S., Leng, S. N., Sikic, M. & Ng, P. C. SIFT missense predictions for genomes. *Nat Protoc*. **11,** 1-9 (2016).
13. Rogers, M. F., Shihab, H. A., Mort, M., Cooper, D. N., Gaunt, T. R. & Campbell, C. FATHMM-XF: accurate prediction of pathogenic point mutations via extended features. *Bioinformatics*. **34,** 511-513 (2018).
14. Adzhubei, I. A. et al. A method and server for predicting damaging missense mutations. *Nat Methods*. **7,** 248-249 (2010).
15. Choi, Y. & Chan, A. P. PROVEAN web server: a tool to predict the functional effect of amino acid substitutions and indels. *Bioinformatics*. **31,** 2745-2747 (2015).
16. Reva, B., Antipin, Y. & Sander, C. Predicting the functional impact of protein mutations: application to cancer genomics. *Nucleic Acids Res*. **39,** e118 (2011).
17. Karczewski, K. J. et al. The mutational constraint spectrum quantified from variation in 141,456 humans. *Nature*. **581,** 434-443 (2020).
18. Lek, M. et al. Analysis of protein-coding genetic variation in 60,706 humans. *Nature*. **536,** 285-291 (2016).
19. Li, J. et al. Performance evaluation of pathogenicity-computation methods for missense variants. *Nucleic Acids Res*. **46,** 7793-7804 (2018).
20. Canibano, C. *et al*. The dependence receptor Ret induces apoptosis in somatotrophs through a Pit-1/p53 pathway, preventing tumor growth. *EMBO J.* **26**, 2015-2028 (2007).

21. Diaz-Rodriguez, E. *et al*. Direct promoter induction of p19Arf by Pit-1 explains the dependence receptor RET/Pit-1/p53-induced apoptosis in the pituitary somatotroph cells. *Oncogene* **31**, 2824-2835 (2012).

22. Garcia, A., Alvarez, C. V., Smith, R. G. & Dieguez, C. Regulation of Pit-1 expression by ghrelin and GHRP-6 through the GH secretagogue receptor. *Mol. Endocrinol.* **15**, 1484-1495 (2001).

23. Soto, J. L., Castrillo, J. L., Dominguez, F. & Dieguez, C. Regulation of the pituitary-specific transcription factor GHF-1/Pit-1 messenger ribonucleic acid levels by growth hormone-secretagogues in rat anterior pituitary cells in monolayer culture. *Endocrinology*. **136,** 3863-3870 (1995).

24. Diaz-Rodriguez, E. *et al*. Somatotropinomas, But Not Nonfunctioning Pituitary Adenomas, Maintain a Functional Apoptotic RET/Pit1/ARF/p53 Pathway That Is Blocked by Excess GDNF. *Endocrinology* **155**, 4329-4340 (2014).

25. Garcia-Rendueles, A. *et al*. Rewiring of the apoptotic TGF-beta-SMAD/NFkappaB pathway through an oncogenic function of p27 in human papillary thyroid cancer. *Oncogene* **36,** 652-666 (2017).

26. Bravo, S. B. et al. Expression of exogenous proteins and short hairpin RNAs in human primary thyrocytes. *Anal Biochem***.** **400,** 219-228 (2010).

27. Bravo, S. B. et al. TGF-beta-induced apoptosis in human thyrocytes is mediated by p27kip1 reduction and is overridden in neoplastic thyrocytes by NF-kappaB activation. *Oncogene*. **22,** 7819-7830 (2003).

28. Bravo, S. B. et al. Humanized medium (h7H) allows long-term primary follicular thyroid cultures from human normal thyroid, benign neoplasm, and cancer. *J Clin Endocrinol Metab*. **98,** 2431-2441 (2013).

29. Carneiro, C., Alvarez, C. V., Zalvide, J., Vidal, A., Domínguez, F. TGF-beta1 actions on FRTL-5 cells provide a model for the physiological regulation of thyroid growth. *Oncogene*. **16,** 1455-1465 (1998).

30. Holden, P. & Horton, W. A. Crude subcellular fractionation of cultured mammalian cell lines. *BMC Res. Notes* **2**, 243 (2009).

31. Chenlo, M. *et al*. Unmasking a new prognostic marker and therapeutic target from the GDNF-RET/PIT1/p14ARF/p53 pathway in acromegaly. *EBioMedicine* **43,** 537-552 (2019).

32. Radonić, A., Thulke, S., Mackay, I. M., Landt, O., Siegert, W. & Nitsche, A. Guideline to reference gene selection for quantitative real-time PCR. *Biochem Biophys Res Commun*. **313,** 856-862 (2004).

33. Schindelin, J. et al. Fiji: an open-source platform for biological-image analysis. *Nat Methods*. **9,** 676-682 (2012).

34. Jones, T. R. et al. CellProfiler Analyst: data exploration and analysis software for complex image-based screens. *BMC Bioinformatics*. **9,** 482 (2008).

35. Waterhouse, A. et al. SWISS-MODEL: homology modelling of protein structures and complexes. *Nucleic Acids Res*. **46,** W296-W303 (2018).

36. Pettersen, E. F. et al. UCSF Chimera a visualization system for exploratory research and analysis. *J Comput Chem*. **25,** 1605-1612 (2004).

37. Yamashita, M. & Emerman, M. Retroviral infection of non-dividing cells: old and new perspectives. *Virology*. **344,** 88-93 (2006).

38. Garcia-Lavandeira, M. *et al*. A GRFa2/Prop1/stem (GPS) cell niche in the pituitary. *PLoS ONE***.** **4,** e4815 (2009).

39. Gremeaux, L., Fu, Q., Chen, J. & Vankelecom, H. Activated phenotype of the pituitary stem/progenitor cell compartment during the early-postnatal maturation phase of the gland. *Stem Cells Dev*. **21,** 801-813 (2012).

40. Koyama, R. A simple method of hypophysectomy in rats (Koyama's external auditory canal method). *Endocrinol Jpn*. **9,** 321-323 (1962).

41. Martin-Rodriguez, J. F. *et al*. Molecular Characterization of Growth Hormone-producing Tumors in the GC Rat Model of Acromegaly. *Sci. Rep.* **5**, 16298 (2015).

42. Ramirez-Yanez, G. O., Smid, J. R., Young, W. G. & Waters, M. J. Influence of growth hormone on the craniofacial complex of transgenic mice. *Eur. J. Orthod.* **27,** 494-500 (2005).

43. Melin, A. D., Bergmann, P. J. & Russell, A. P. Mammalian Postnatal Growth Estimates: The Influence of Weaning on the Choice of a Comparative Metric. *J Mammal*. **86,** 1042-1049 (2005).

44. Wang, J., Zhou, J., Cheng, C. M., Kopchick, J. J. & Bondy, C. A. Evidence supporting dual, IGF-I-independent and IGF-I-dependent, roles for GH in promoting longitudinal bone growth. *J Endocrinol.* **180,** 247-255 (2004).

45. Barry, S. *et al*. Tumor microenvironment defines the invasive phenotype of AIP-mutation-positive pituitary tumors. *Oncogene* **38,** 5381-5395 (2019).

46. Gaston-Massuet, C. *et al*. Genetic interaction between the homeobox transcription factors HESX1 and SIX3 is required for normal pituitary development. *Dev. Biol.* **324,** 322-333 (2008).

47. Arighi, E. et al. Identification of Shc docking site on Ret tyrosine kinase. *Oncogene*. **14,** 773-782 (1997).

48. Bordeaux, M. C. *et al*. The RET proto-oncogene induces apoptosis: a novel mechanism for Hirschsprung disease. *EMBO J.* **19,** 4056-4063 (2000).

49. Soh, J. W. & Weinstein, I. B. Roles of specific isoforms of protein kinase C in the transcriptional control of cyclin D1 and related genes. *J Biol Chem*. **278,** 34709-34716 (2003).

50. Stewart, S. A. et al. Lentivirus-delivered stable gene silencing by RNAi in primary cells. *RNA*. **9,** 493-501 (2003).

51. Pear, W. S. et al. Efficient and rapid induction of a chronic myelogenous leukemia-like myeloproliferative disease in mice receiving P210 bcr/abl-transduced bone marrow. *Blood*. **92,** 3780-3792 (1998).
